# Rejuveinix Shows a Favorable Clinical Safety Profile in Human Subjects and Exhibits Potent Preclinical Protective Activity in the Lipopolysaccharide-Galactosamine Mouse Model of Acute Respiratory Distress Syndrome and Multi‐Organ Failure

**DOI:** 10.3389/fphar.2020.594321

**Published:** 2020-11-10

**Authors:** Fatih M. Uckun, James Carlson, Cemal Orhan, Joy Powell, Natalie M. Pizzimenti, Hendrik van Wyk, Ibrahim H. Ozercan, Michael Volk, Kazim Sahin

**Affiliations:** ^1^ Drug Discovery Program, Reven Pharmaceuticals, Golden, CO, United States; ^2^ Department of Developmental Therapeutics, Immunology, and Integrative Medicine, Ares Pharmaceuticals, St. Paul, MN, United States; ^3^ Department of Animal Nutrition, Faculty of Veterinary, Firat University, Elazig, Turkey; ^4^ Department of Pathology Faculty of Medicine, Firat University, Elazig, Turkey

**Keywords:** cancer, sepsis, pneumonia, acute lung injury, multi-organ dysfunction, cytokine release syndrome, COVID-19

## Abstract

**Background:** New treatment platforms that can prevent acute respiratory distress syndrome (ARDS) or reduce its mortality rate in high-risk coronavirus disease 2019 (COVID-19) patients, such as those with an underlying cancer, are urgently needed. Rejuveinix (RJX) is an intravenous formulation of anti-oxidants and anti-inflammatory agents. Its active ingredients include ascorbic acid, cyanocobalamin, thiamine hydrochloride, riboflavin 5′ phosphate, niacinamide, pyridoxine hydrochloride, and calcium D-pantothenate. RJX is being developed as an anti-inflammatory and anti-oxidant treatment platform for patients with sepsis, including COVID-19 patients with viral sepsis and ARDS. Here, we report its clinical safety profile in a phase 1 clinical study (ClinicalTrials.gov Identifier: NCT03680105) and its potent protective activity in the lipopolysaccharide galactosamine (LPS-GalN) mouse model of ARDS.

**Methods:** A phase 1, double-blind, placebo-controlled, randomized, two-part, ascending dose-escalation study was performed in participating 76 healthy volunteer human subjects in compliance with the ICH (E6) good clinical practice guidelines to evaluate the safety, tolerability, pharmacokinetics, and pharmacodynamics of RJX (Protocol No. RPI003; ClinicalTrials.gov Identifier: NCT03680105). The ability of RJX to prevent fatal shock, ARDS, and multi-organ failure was examined in the well-established LPS-GalN mouse model of sepsis and ARDS. Standard methods were employed for the statistical analysis of data in both studies.

**Findings:** In the phase 1 clinical study, no participant developed serious adverse events (SAEs) or Grade 3-Grade 4 adverse events (AEs) or prematurely discontinued participation in the study. In the non-clinical study, RJX exhibited potent and dose-dependent protective activity, decreased the inflammatory cytokine responses (interleukin-6, tumor necrosis factor alpha, transforming growth factor beta), and improved survival in the LPS-GalN mouse model of sepsis and ARDS. Histopathological examinations showed that RJX attenuated the LPS-GalN induced acute lung injury (ALI) and pulmonary edema as well as liver damage.

**Conclusion: **RJX showed a very favorable safety profile and tolerability in human subjects. It shows potential to favorably affect the clinical course of high-risk COVID-19 by preventing ARDS and its complications.

## Introduction

A significant minority of patients infected with the new coronavirus, SARS-CoV-2, the causative agent of coronavirus disease 2019 (COVID-19), develop viral pneumonia that causes an acute lung injury (ALI) capable of rapid progression to viral sepsis and acute respiratory distress syndrome (ARDS) with a high fatality rate especially if they are older and have comorbidities (Carver and Jones, 2020; Kuderer et al., 2020; Lian et al., 2020; Uckun, 2020a; Uckun, 2020b; Wu et al., 2020; Zheng et al., 2020; Zhou et al., 2020). A systemic inflammatory response syndrome, also referred to as cytokine storm or cytokine release syndrome (CRS), contributes to the development of ARDS and often irreversible multi-organ dysfunction syndrome (MODS) associated with the severe-critical forms of COVID-19 (Uckun, 2020b). Approximately 20% of COVID-19 patients with mild-moderate disease progress to severe-critical disease, and this percentage increases to 40% for the high-risk subgroup ≥65 years of age with comorbidities or laboratory parameters indicative of systemic inflammation, such as high levels of C-reactive protein (CRP), lactate dehydrogenase (LDH), and ferritin or dysfunction of the coagulation system, as evidenced by elevated D-dimer levels (Uckun, 2020a). High-risk patients not only have a higher incidence of ARDS owing to severe viral sepsis caused by SARS-CoV-2, but they also progress faster and have a significantly higher case mortality rate (Carver and Jones, 2020; Kuderer et al., 2020; Lian et al., 2020; Uckun, 2020a; Uckun, 2020b; Wu et al., 2020; Zheng et al., 2020; Zhou et al., 2020). COVID-19 patients with an underlying cancer, especially if they are undergoing chemotherapy, are at an augmented risk for developing potentially fatal ARDS and multi-organ failure (Kuderer et al., 2020; Uckun, 2020a). Therefore, treatment platforms capable of preventing the disease progression and/or reducing the case mortality rate in such high-risk COVID-19 patients are urgently needed (Kuderer et al., 2020; Uckun, 2020a).

Rejuveinix (RJX) (Van Wyk et al.) is a formulation of several vitamins, including ascorbic acid (Vitamin C), cyanocobalamin (Vitamin B12), thiamine hydrochloride (Vitamin B1), riboflavin 5′ phosphate (Vitamin B2), niacinamide (Vitamin B3), pyridoxine hydrochloride (Vitamin B6), calcium D-pantothenate, and magnesium sulfate, representing components that have been studied in animal models of septic shock and ARDS as well as clinical studies in septic patients (Lev et al., 2007; Wang et al., 2014; Li, 2018; Woolum et al., 2018; Hager et al., 2019; Iglesias et al., 2020; Pan et al., 2019; Putzu et al., 2019; Noormandi et al., 2020; Obi et al., 2020; Shan et al., 2020; Uckun et al., 2020). Here we are reporting for the first time the primary safety and pharmacokinetics (PK) data from a phase 1 clinical study of RJX and the results of a nonclinical pharmacodynamics study that evaluated its activity in a mouse model of sepsis, ARDS, and multi-organ failure. The main objective of the clinical study was to evaluate the clinical tolerability of RJX in a double-blind, placebo-controlled phase 1 clinical trial in healthy volunteer subjects (ClinicalTrials.gov Identifier: NCT03680105). We show that RJX has a very favorable safety profile in human subjects. In the nonclinical pharmacodynamic study we sought to determine if RJX can improve the survival outcome of mice challenged with an otherwise invariably fatal dose of lipopolysaccharide (LPS)-galactosamine (GalN) in a model of sepsis, ARDS and multi-organ failure. RJX exhibited potent protective anti-CRS and anti-ARDS activity in the LPS-GalN model at clinically safe low dose levels.

## Materials and Methods

### Rejuveinix

RJX is a proprietary composition of naturally occurring anti-oxidants and anti-inflammatory agents which, in combination, provide potent and immediate tissue protection. Its ingredients include ascorbic acid, magnesium sulfate heptahydrate, cyanocobalamin, thiamine hydrochloride, riboflavin 5′ phosphate, niacinamide, pyridoxine hydrochloride, and calcium D-pantothenate. RJX is a two-vial system, and A and B are each of the two vials. Vial A contains the active ingredients and minerals, whereas Vial B contains the buffer, sodium bicarbonate as the Vial A content is acidic. The components of each of the vials are shown in Supplementary Table S1. RJX is being developed as an anti-inflammatory and anti-oxidant treatment platform for patients with sepsis, including COVID-19 patients with viral sepsis and ARDS (Van Wyk et al.).

### Ethics Statement, Approval, and Conduct of the Clinical Phase 1 Study

The safety profile, PK, and pharmacodynamics of RJX were evaluated in a placebo-controlled, double-blind phase 1 dose-escalation study in healthy volunteers (ClinicalTrials.gov Identifier: NCT03680105). This was a two part study with part 1 single asending doses (n = 52) and part 2 multiple ascending doses (MADs) (n = 24) (Supplementary Table S2). The study was performed at the clinical facility of the ICON Early phase Services, LLC, 8307 Gault Lane, San Antonio, TX 78209 USA, in compliance with the ICH (E6) good clinical practice guidelines, in adherence to the ethical principles based on the Declaration of Helsinki, and the applicable national and local laws and regulatory requirements. Date of first enrollment: 8/27/2018; Date of last completed: 2/16/2019. The study was approved by the IntegReview IRB (3815S. Capital of Texas Hwy, Suite 320, Austin, TX 78704, IRB Registration Numbers: IRB IRB00008463, IRB00003657, IRB00004920, IRB00001035, IRB00006075). Each subject provided a written informed consent. A safety review committee reviewed the safety data for each cohort prior to escalation to the next cohort. The details of the subject demographics, safety assessments, PK analyses, and cardio-dynamic studies are detailed in the Supplemental Material.

The starting dose was based on the results of animal toxicity studies. The no observed adverse effect level (NOAEL) was >5 ml/kg in both Sprague Dawley rats (when administered via intra-arterial route daily for 21 days) and Beagle dogs (when administered via intra-arterial route daily × 28 days). The HED of the >5 ml/kg NOAEL in rats is 0.8 ml/kg and the HED of the ≥5 ml/kg NOAEL in dogs is 2.7 ml/kg. The starting dose of 0.024 ml/kg in the phase 1 study represents the HED in the most sensitive species divided by a safety factor of 30 (i.e., 33-times lower than the NOAEL in rats and >112.5-fold lower than the NOAEL in dogs).

Part 1 was a single ascending dose (SAD) escalation study in 52 participants, six cohorts in total. Cohorts 1 to 4 included eight participants per cohort (6 RJX: 2 placebo) aged 18–50 years, inclusive (Supplementary Table S3). Cohort 6 investigated an older population of 12 participants (9 RJX: 3 placebo) aged 51–70 years, inclusive (Supplementary Table S3). Part 2 of the study was a MAD escalation study in 24 participants, three cohorts of eight participants (6 RJX: 2 placebo).The MAD arm of the study commenced in parallel with Part 1, Cohort 6, following completion and review of the safety and PK findings for Part 1, Cohorts 1 through 5. Each patient in the SAD cohort received a single intravenous infusion of either placebo or RJX at escalating doses. The MAD cohort subjects were randomly assigned to receive RJX at one of three dose levels or placebo (6  RJX: 2  placebo) every day for 7 days. The assigned RJX dose was administered intravenously in a total volume of 100 ml normal saline (NS) over 45 min (±5 min).

Placebo was used in both Part 1 (SAD study) and Part 2 (MAD study). The placebo was NS (i.e., an aqueous solution of 0.9% NaCl). In the phase 1 Clinical trial, RJX was given intravenously at ascending doses for SAD of (cohort 1) 0.024 ml/kg, (cohort 2) 0.076 ml/kg, (cohort 3) 0.240 ml/kg, (cohort 4) 0.500 ml/kg, (cohort 5) 0.759 ml/kg, and the elderly cohort (cohort 6) 0.500 ml/kg RJX. In the MAD group, RJX was given intravenously at doses of 0.240 ml/kg, 0.500 ml/kg, and 0.759 ml/kg (Supplementary Table S2). Each patient in the SAD cohort received a single intravenous infusion of either placebo or RJX at escalating doses. The MAD cohort subjects were randomly assigned to receive RJX at one of three dose levels or placebo (6  RJX: 2  placebo) every day for 7 days. The appropriate RJX dose was calculated based on ml/kg, and it was prepared for intravenous administration by adding the appropriate dose of RJX to NS in a total volume of 100 ml. 100 ml NS served as the Placebo. Placebo was administered as 0.9% IV saline in a 100 ml total volume over 45 min (±5 min). In Part 1 (SAD), RJX was administered as a 100 ml IV infusion over 45 min (±5 min) on a single occasion on Day 1. In Part 2 (MAD), RJX was administered as a 100 ml IV infusion over 45 min (±5 min), as in Part 1, every day for 7 days.

The pharmacokinetic blood sampling in Part 1 (SAD study) was done at a total of 15 time points, including three time points prior to the RJX infusion (i.e., within 90 ± 2 min prior to, within 60 ± 2 min prior to and 30 ± 2 min prior to RJX), plus three time points after start of the RJX infusion (i.e., 15 ± 2 min post start, 30 ± 2 min post start, and 45 ± 2 min post start), plus nine time points after the end of the RJX infusion (30 ± 2 min post-end, 1 h ± 2 min post-end, 1.5 h ± 2 min post-end, 2 h ± 2 min post-end, 4 h ± 2 min post-end, 8 h ± 5 min post-end, 10 h ± 5 min post-end, 12 h ± 10 min post-end, and 24 h ± 10 min post-end). In Part 2 (MAD study), the same 15 time points were used on day 1 and day 7. In addition, blood samples were also obtained on days 4 and 6 within 30 ± 2 min prior to the RJX infusion.

### Statistical Analyses for Clinical Study

Statistical analysis of data was performed using standard methods. Mean, median and standard deviation were reported along with n’s of categorical data (see Supplemental Material). Clinical laboratory safety parameters, vital sign measurements, and 12-lead safety ECG parameters were listed and summarized using descriptive statistics for each treatment, timepoint, and changes from baseline. The bioanalytical studies were performed at Altasciences Company Inc. in Laval, Quebec, Canada. Concentration-time profiles of ascorbic acid, cyanocobalamin, niacinamide and thiamine in plasma and magnesium in serum were analyzed by noncompartmental methods using Phoenix^®^ WinNonlin^®^ (version 8.0) (Cetara), model 200-202 for intravenous injection. PK parameters calculated for each active ingredient (except riboflavin 5′ phosphate, pyridoxine hydrochloride, calcium D-pantothenate) included t_max_, Cmax, AUC_0-last_, and t_1/2_.

### Lipopolysaccharide-Galactosamine Model of Fatal Cytokine Storm and Acute Respiratory Distress Syndrome

The ability of RJX to prevent fatal shock, ARDS, and multi-organ failure was examined in the well-established LPS-GalN model, as described in detail in the Supplemental Material. In brief, LPS was combined with GalN, which further sensitizes mice to LPS-induced systemic inflammatory syndrome and multi-organ failure (Uckun et al., 2008). The care and treatment of the animals were in accordance with the *Guide for the Care and Use of Laboratory Animals*. They were approved by the Animal Care and Use Committee of Firat University (Project No. 04052020-391-046). BALB/c mice were randomly divided into six treatment groups with 10 mice in each group. All mice were genetically identical, of the same age, and the LPS-GalN challenged mice in Groups 2–6 were injected with the same amount of LPS-GalN. This statistical equivalency of mice allowed the use of a pseudo-randomization convenience allocation to assign mice to identified cages. For random treatment allocation, cages were randomly selected to receive one of the specified treatments. We applied concealment of treatment allocation and blind outcome assessment to reduce the risk of bias in our conclusions. Health care assessments were performed by animal care technicians not involved in the treatment assignments or treatments. Investigators did not participate in individual health status or outcome assessments.

In the LPS-GalN mouse model, mice were treated via intraperitoneal injections of 6-fold diluted (vehicle = NS) RJX (0.5, 1.05, 2.1, 4.2 ml/kg, 0.5 ml/mouse) or NS 2 h before and 2 h post-injection of LPS-GalN (see also Supplemental Material). Untreated normal control mice (Group 1) did not receive any treatments. Vehicle control mice (Group 2) were treated with 500 µl NS, i.e., an aqueous solution of 0.9% NaCl with no RJX. NS was administered intraperitoneally (*i.p,*) 2 h before and 2 h after the *i.p.* injection of LPS-GalN. Test mice (Groups 3–6) received the designated RJX dose (500 µl/mouse) at the indicated dose levels (0.5, 1.05, 2.1, 4.2 ml/kg of a 6-fold diluted RJX solution) 2 h before and 2 h after the *i.p.* injection of LPS-GalN (see also Supplemental Material). Safety pharmacology labs, serum and tissue cytokine levels, tissue activity levels of superoxide dismutase (SOD), catalase (CAT) and glutathione peroxidase (GSH-Px), tissue ascorbic acid levels, as well as tissue concentrations of malondialdehyde (MDA) were determined to examine the effects of RJX on the inflammatory process caused by LPS-GaN. interleukin 6 (IL-6) and tumor necrosis factor alpha (TNF-α) levels in mouse serum samples were measured by quantitative ELISA using the commercially available “Mouse IL-6 Quantikine ELISA Kit” (Sensitivity for mouse IL-6: 1.6 pg/ml, Assay Range: 7.8–500 pg/ml) and “Mouse TNF-α Quantikine ELISA Kit” (Sensitivity for mouse TNF-α: 7.21 pg/ml, Assay Range: 10.9–700 pg/ml), respectively, and an absorbance microplate reader (Bio-Tek Elx800 universal Microplate Reader, Bio-Tek Instruments, Inc., Winooski, USA) according to the manufacturer’s instructions (R&D Systems, Minneapolis, MN, USA). Samples and standards were analyzed in duplicate. The Kaplan-Meier method, log-rank chi-square test was used to analyze the 48 h survival outcomes of mice in the different treatment groups. At the time of death, lungs, heart, kidneys, liver, and brain from six mice per group were harvested, fixed in 10% buffered formalin, and processed for histopathologic examination. In another experiment, treatments with vehicle (NS) or RJX were delayed until 2 h post LPS-GalN. Groups of six mice were treated with i.p. injections of 4.2 ml/kg NS or 6-fold diluted RJX at 2 and 3 h (total volume: 500 µl/mouse). Mice were monitored for survival and serum cytokine levels as well as lung MDA levels were measured at the time of death or elective termination at 24 h post LPS-GalN injection. The Kaplan-Meier method, log-rank chi-square test was used to analyze the 24 h survival outcomes of mice in the two treatment groups. This experimental design was basd on a pilot experiment, in which serum cytokine levels and lung MDA levels were documented to be markedly elevated at 2 h after LPS-GalN injection in six of six mice, when compared to the levels measured in six control untreated mice.

### Statistical Analyses for Non-clinical Studies in the Lipopolysaccharide-Galactosamine Mouse Model of Cytokine Storm, Acute Respiratory Distress Syndrome and Multi-Organ Failure

The Decision statistical structure includes ANOVA using SPSS statistical program (IBM, SPPS Version 21) and/or covariance, nonparametric analysis of variance, simple t-tests, Tukey’s Test for homogeneity of variance, and pairwise tests by the Dunnett’s test for parametric and nonparametric data. The Tukey’s multiple comparisons detected alterations among groups. Furthermore, the Kaplan-Meier method, log-rank chi-square test, was used to investigate survival and fatality in each group.

## Results

### Clinical Safety, Tolerability, Pharmacokinetics, and Pharmacodynamics of Rejuveinix

A phase 1, double-blind, placebo-controlled, randomized, two-part, ascending dose-escalation study was performed to evaluate the safety, tolerability, and PK of RJX in healthy volunteers (Protocol No. RPI003; ClinicalTrials.gov Identifier: NCT03680105) (Supplementary Tables S2, S3). The dose levels ranged from 0.024 ml/kg to 0.759 ml/kg in Part 1 and from 0.240 ml/kg to 0.759 ml/kg in Part 2. The treatment-emergent AEs (TEAEs) are presented in Supplementary Tables S4–S7. In Part 1, no deaths or serious adverse event (SAEs) were reported, none of the 39 RJX-treated subjects experienced Grade 3 or 4 adverse events (AEs), and no AEs led to discontinuation of RJX (Supplementary Table S7A). In Part 2, no SAEs and no Grade 3 or 4 AEs were encountered by any of the 18 RJX-treated subjects. One subject in Cohort 1 developed a mild upper respiratory infection; three subjects in Cohort 2 had mild infusion site discomfort, pain or reaction; and three subjects on Cohort 3 experienced mild-moderate pain (mild headache = 1; mild pain in extremity = 1, moderate back pain = 1). The TEAEs in two of the subjects were considered possibly or probably related to RJX. Both patients were in Cohort 2: One patient had a mild infusion site discomfort on day 6 considered possibly related to RJX and another patient had a mild infusion site pain on days 6 and 7 considered probably related to RJX infusion (Supplementary Tables S6, S7). These TEAEs did not require additional treatment, and they did not cause discontinuation of the dosing or study. All AEs considered possibly or probably related to RJX infusions have recovered/resolved with no sequelae. There were no clinically significant abnormal findings in 12-lead safety ECGs, and no notable changes were detected compared with baseline. For both study parts, there were no clinically meaningful changes in laboratory values identified in observed values or mean changes from baseline compared with placebo or evaluated by increasing the RJX dose level. Based on its tolerability, the 0.500 ml/kg dose level was selected as the recommended phase 2 dose (RP2D) level for future studies.

**FIGURE 1:**
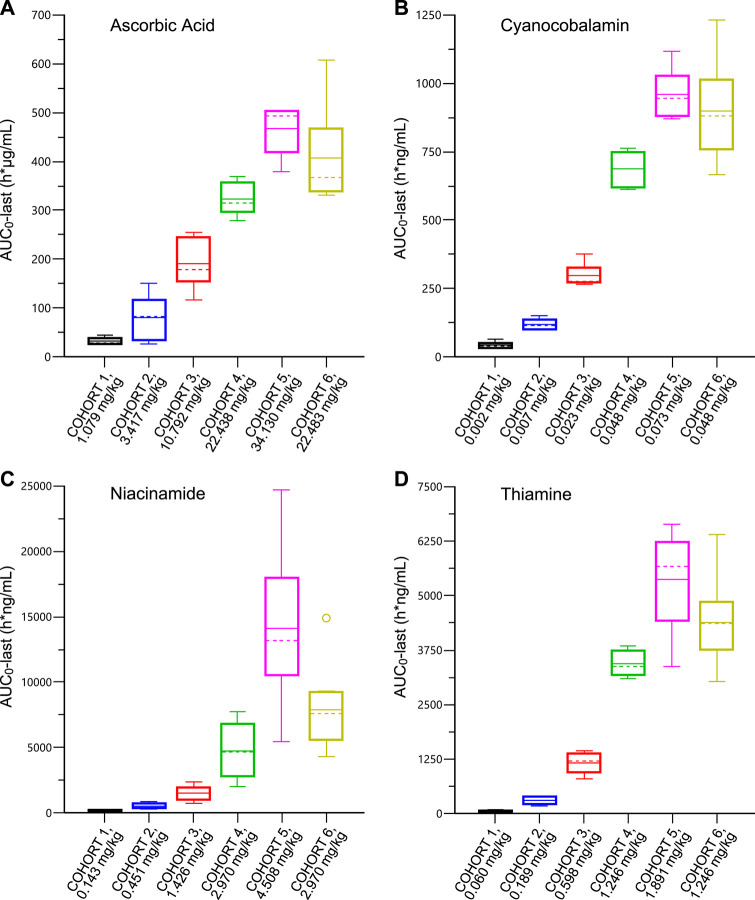
Plasma Pharmacokinetic Parameters for Ascorbic Acid **(Panel A)**; Cyanocobalamin **(Panel B)**; Niacinamide **(Panel C)**; Thiamine **(Panel D)** after Single Ascending Dose of IV Infusion of Rejuveinix in Healthy Adult and Elderly Volunteers. See text for discussion.

A summary of RJX plasma PK parameters by cohort following SAD IV infusion is presented in Supplementary Tables S8–S12. Plasma concentration-time profiles of baseline-adjusted ascorbic acid (A), cyanocobalamin (B), niacinamide (C) and thiamine (D) for the RJX dose level of 0.5 ml/kg (Cohort 6) following single-dose IV infusion of RJX are presented in Supplementary Figure S1 and box plots for AUC_0-last_ are shown in Figure 1. The mean baseline plasma concentrations of ascorbic acid ranged from 7.99 to 10.12 μg/ml for all cohorts. Following the SAD administration of RJX in healthy subjects, baseline-adjusted ascorbic acid C_max_ and AUC_0-last_ increased with increasing doses of RJX (Supplementary Table S8). The mean C_max_ ranged from 5.40 μg/ml [0.024 ml/kg (1.079 mg/kg of ascorbic acid)] to 152.69 μg/ml (0.759 ml/kg [34.130 mg/kg of ascorbic acid]), increasing 28-fold while the mean AUC_0-last_ ranged from 32.36 h*µg/ml to 467.10 h*µg/ml, increasing 14-fold for a 32-fold increase in dose. At the RJX dose level of 0.5 ml/kg (22.483 mg/kg of ascorbic acid), C_max_ and AUC_0-last_ increased by 1.2 to 1.3-fold in elderly subjects when compared to healthy subjects. The plasma RJX PK parameters for cyanocobalamin, magnesium sulfate, niacinamide, and thiamine by cohort following single-dose IV infusion are presented in Supplementary Tables S9–S12. At the dose level of 0.5 ml/kg (0.048 mg/kg of cyanocobalamin; 2.970 mg/kg of niacinamide; 1.246 mg/kg of thiamine), the C_max_ and the AUC_0-last_ of cyanocobalamin, niacinamide, and thiamine increased by 1.3‐fold, 1.7‐fold, and 1.3‐fold respectively, in elderly subjects in Cohort 6 when compared to younger healthy subjects in Cohort 4. At the same dose level, the C_max_ and the AUC_0-last_ of magnesium increased by 1.2 and 1.6-fold, respectively, in elderly subjects when compared to younger subjects.

The plasma concentration-time profiles of baseline-adjusted ascorbic acid, cyanocobalamin, niacinamide, and thiamine for the RJX dose level of 0.5 ml/kg (Cohort 6) following single-dose IV infusion of RJX are presented in Supplementary Figure S1. The terminal phase following single-dose administration of ascorbic acid and magnesium could not be adequately characterized. The half-life ranged from 5 to 28 h, 0.4–1.5 h, and 2–10 h, for cyanocobalamin, niacinamide, and thiamine, respectively. There was no clinically significant accumulation of these compounds following once-daily IV infusion for 7 days in the part 2 MAD cohorts, as demonstrated by the PK profiles on Day 7 that were overall similar to those observed on Day 1 (Supplementary Tables S13–S17).

In cardio-dynamic ECG asessments, we examined the potential effects of RJX on the QT interval corrected by the Fridericia correction formula (QTcF). RJX at the studied doses caused mild QTc prolongation but it did not have a clinically relevant effect on any other ECG parameter, i.e., heart rate, PR and QRS intervals. In the by-time point analysis of QTcF, mean ΔQTcF on RJX generally followed the placebo pattern across post-dose time points in both parts of the study. For the pooled data from both parts of the study (SAD plus MAD), a full model including ascorbic acid, cyanocobalamin, magnesium, niacinamide, thiamine and their 1-by-1 interaction terms was initially fitted and a model selection procedure was performed. The model with ascorbic acid and magnesium alone was selected as the primary model and represented the data reasonably well. The estimated slopes of the concentration-QTc relationship were 0.012 ms per mg/l (90% CI: −0.0114–0.0346) for ascorbic acid and 0.34 ms per mg/l (90% CI: 0.081–0.606) for magnesium, of which the slope of magnesium was statistically significant. The treatment effect-specific intercept was −5.06 ms (90% CI: −10.244 to 0.126) and not statistically significant. The predicted placebo-corrected QT effects (∆ΔQTcF) were predicted to be 7.12 ms (90% CI: 4.54–9.70) for the SAD 0.759 ml/kg group on Day 1 and 9.13 ms (90% CI: 6.08–12.19) for the MAD 0.759 ml/kg group on Day 7 where the highest levels of plasma concentrations were observed for each part (Supplementary Table S18). The prediction results from other models with each analyte alone were similar to the results from the primary model. The predicted, placebo-conrrected ΔΔQTcF values at the geometric mean peak concentration are provided for the concentration-QTc models with each analyte alone in Supplementary Tables S19–S23; Supplementary Figures S2–S6. Based on these concentration-QTc analyses, an effect on ΔΔQTcF exceeding 10 ms can be excluded within the observed ranges of plasma concentrations of ascorbic acid, cyanocobalamin, magnesium, niacinamide, and thiamine up to 166 mg/L, 362 μg/L, 4.0 mg/dl, 7570 μg/L, and 4890 μg/L, respectively. Such plasma concentrations were only observed at the 0.759 ml/kg dosing condition, thus an effect on the QTc interval >10 ms cannot be excluded at this dose level.

### Dose-dependent *in Vivo* Protective Activity of Prophylactic RJX Treatments in the Lipopolysaccharide-Galactosamine Model of Acute Respiratory Distress Syndrome and Multiorgan Failure

**FIGURE 2:**
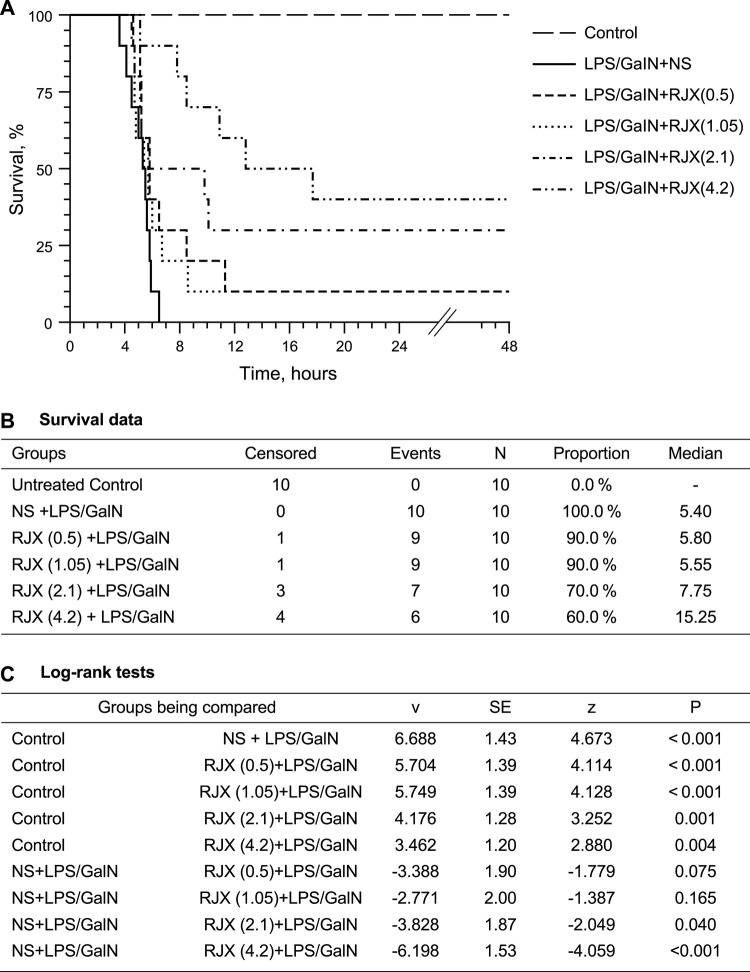
Dose-Dependent *In Vivo* Protective Activity of Prophylactic Rejuveinix (RJX) Treatments in the Lipopolysaccharide-Galactosamine (LPS-GalN) Model of acute respiratory distress syndrome (ARDS) and Multiorgan Failure. Groups of 10 BALB/C mice were treated with i.p injections of RJX (0.5, 1.05, 2.1, 4.2 ml/kg of 6‐fold diluted RJX, 0.5 ml/mouse) or vehicle (NS) 2 h before or 2 h post-injection of LPS-GalN. Except for untreated control mice (Control), each mouse received 0.5 ml of LPS-GalN (consisting of 100 ng of LPS plus 8 mg of D-galactosamine) i.p. The cumulative proportion of mice remaining alive (Survival, %) is shown as a function of time after the LPS-GalN challenge. Depicted are the Kaplan Meier survival curves **(Panel A)** and survival data with statistical analysis **(Panels B,C)** of the different treatment groups. The RJX dose levels (in ml/kg) are indicated in parentheses.

One hundred (100) percent (%) of untreated control mice remained alive throughout the experiment (Group 1). By comparison, 100% of LPS-GalN alone injected mice died at a median of 5.4 h (Group 2) (Figure 2). RJX-treated mice had an improved survival outcome after being injected with LPS-GalN. RJX reduced the incidence of fatal outcome and delayed the time to death in a dose-dependent manner. In contrast to the invariably fatal treatment outcome of vehicle-treated control mice, which had a median survival time of 5.4 h, 40% of mice treated with 4.2 ml/kg of 6-fold diluted RJX (N = 10) remained alive and the median survival time was prolonged to 15.25 h (Log-rank *p* = 0.004; Z-score: ‐4.059, *p* < 0.001) (Figure 2). Hence, RJX exhibited potent and dose-dependent activity in the LPS-GalN model of ARDS and multi-organ failure in mice. The most effective dose was the highest dose level, 4.2 ml/kg of a 6-fold diluted RJX, which corresponds to a human equivalent dose of 0.05 ml/kg (0.3 ml/kg:6), which is 10-times lower than the 0.5 ml/kg RP2D level of RJX (Nair and Jacob, 2016).

**FIGURE 3:**
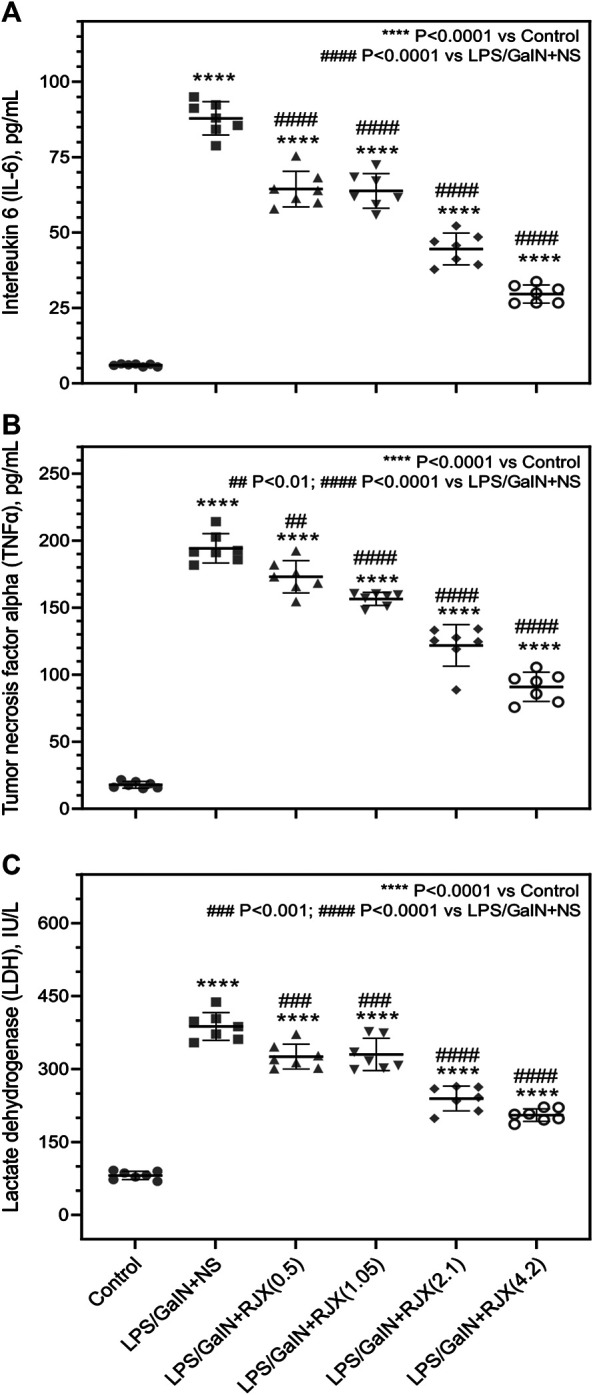
Rejuveinix (RJX) Prevents the Surge of Inflammatory Markers in the Blood Following Lipopolysaccharide-Galactosamine (LPS-GalN) Challenge in a Mouse Model of acute respiratory distress syndrome (ARDS) and Multi-organ Failure. Mice were treated with i.p injections of RJX (0.5, 1.05, 2.1, 4.2 ml/kg of 6‐fold diluted RJX, 0.5 ml/mouse) or normal saline (NS) 2 h before and 2 h post-injection of LPS-GalN. Except for untreated mice (Control), each mouse received 0.5 ml of LPS-GalN (consisting of 100 ng of LPS plus 8 mg of D-galactosamine) i.p. The RJX dose levels (in ml/kg) are indicated in parentheses. See text for discussion of results. The depicted lines represent the mean and standard deviation for the indicated inflammatory markers, namely serum IL‐6 **(Panel A)**, TNF‐a **(Panel B)**, and LDH levels **(Panel C)**. ANOVA and Tukey’s post-hoc test were used for comparing the results among different treatment groups. Statistical significance between groups is shown by: ****p < 0.0001 compared as control group and, #*p* < 0.05; ##*p* < 0.01; ####*p* < 0.0001 compared as LPS/GaIN + NS group).

In LPS-GalN challenged control mice not receiving any RJX treatments, serum IL-6, TNF-α and LDH levels were drastically increased at the time of death which is consistent with a cytokine storm and marked systemic inflammation (*p* < 0.0001 for IL-6, TNF-α, and LDH when compared to Group 1) (Figure 3). RJX treatments had a statistically significant and dose-dependent lowering effect on the serum levels of the inflammation markers (IL6, TNF-α, and LDH) (Figure 3). These results of mice that died within 24 h after the LPS-GalN challenge demonstrate that RJX decreased the inflammatory cytokine responses of mice injected with LPS-GalN, and thereby improved the survival time of mice in a dose-dependent fashion.

**FIGURE 4:**
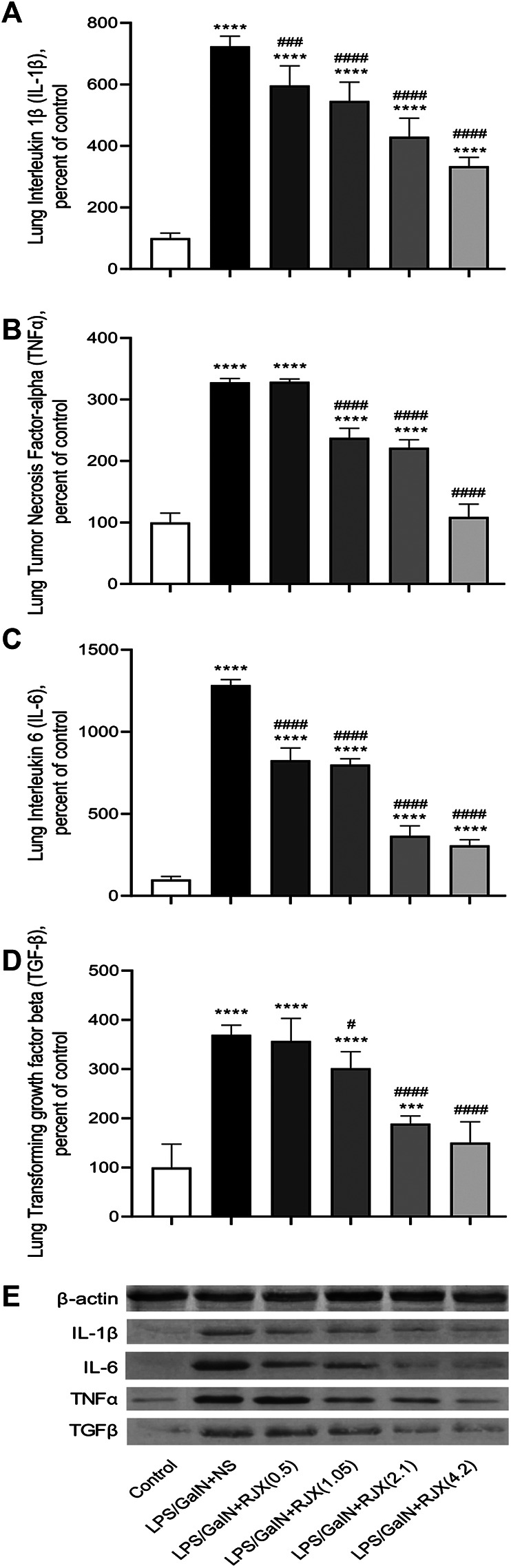
Rejuveinix (RJX) Prevents Pro-inflammatory Cytokine Response in the Lungs of Mice Challenged with Lipopolysaccharide-Galactosamine (LPS-GalN). Mice were treated with i.p injections of RJX (0.5, 1.05, 2.1, 4.2 ml/kg, 0.5 ml/mouse) or normal saline (NS) 2 h before and 2 h post-injection of LPS-GalN. Except for untreated mice (Control), each mouse received 0.5 ml of LPS-GalN (consisting of 100 ng of LPS plus 8 mg of d-galactosamine) i.p. The RJX dose levels (in ml/kg) are indicated in parentheses. See text for discussion of results. Depicted are the results of Western blot analyses of cytokine expression in the pooled lung tissue samples from mice in various treatment groups. Panel A: Lung IL‐1b levels; Panel B: Lung TNF‐a levels; Panel C: Lung IL‐6 levels; D: Lung TGF‐b levels; E: Immunoblot. Results are expressed as percent of control with the expression level in the lung tissue sample from untreated control mice taken as 100% for comparisons. The bar represents mean and standard deviation. Immunoblotting with an anti-actin antibody was used to ensure equal protein loading. (ANOVA and Tukey’s post-hoc test. Statistical significance between groups is shown by: ***p < 0.001; ****p < 0.0001 compared as control group and, #*p* < 0.05; ###*p* < 0.001; ####*p* < 0.0001 compared as LPS/GaIN + NS group).

**FIGURE 5:**
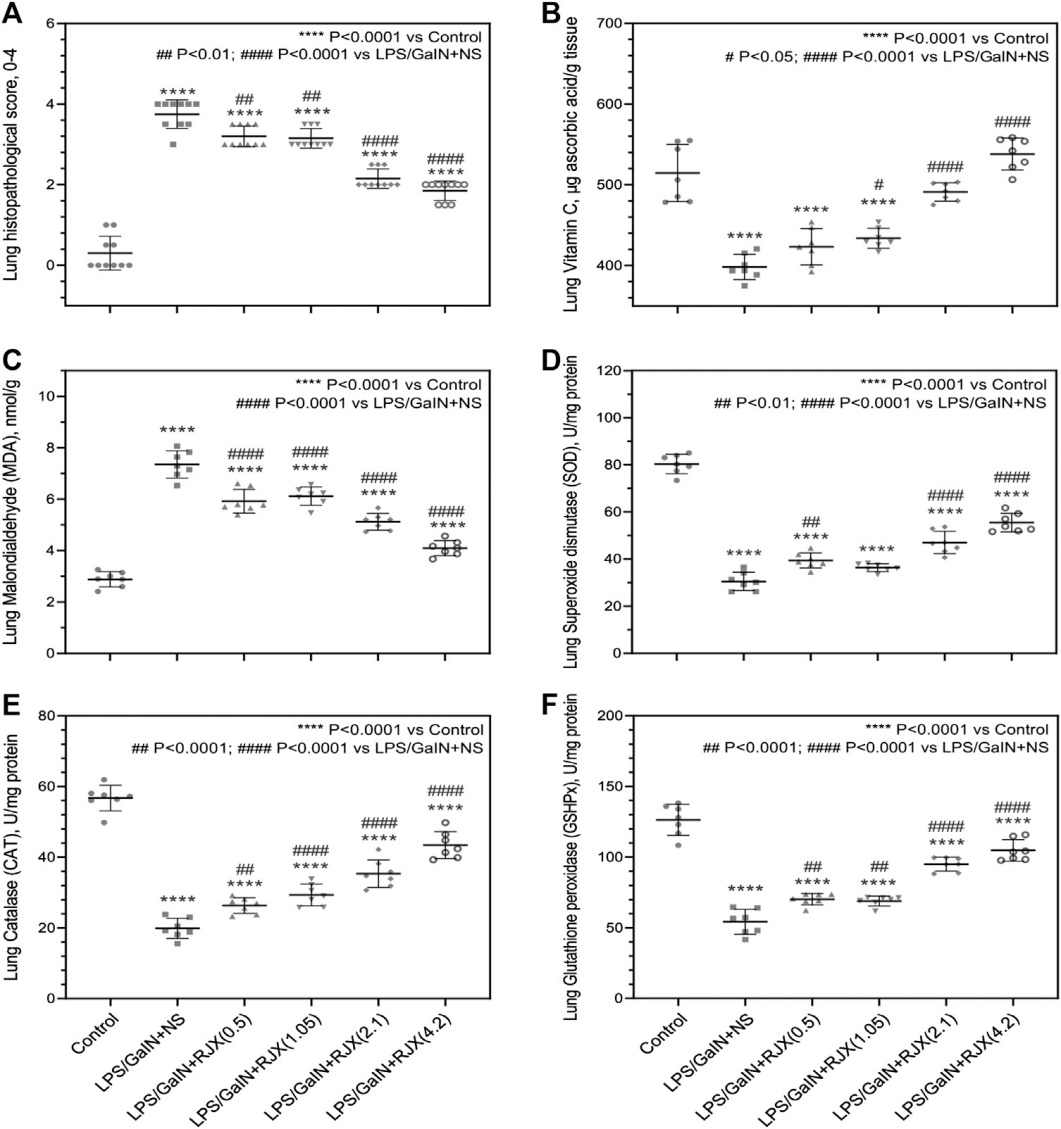
Tissue-Level *In Vivo* Anti-Inflammatory and Anti-Oxidant Activity of Rejuveinix (RJX) in the Lipopolysaccharide-Galactosamine (LPS-GalN) Mouse Model of acute respiratory distress syndrome (ARDS) and Multi-organ Failure. Mice were treated with i.p injections of RJX (0.5, 1.05, 2.1, 4.2 ml/kg, 0.5 ml/mouse) or normal saline (NS) 2 h before and 2 h post-injection of LPS-GalN. Except for untreated mice (Control), each mouse received 0.5 ml of LPS-GalN (consisting of 100 ng of LPS plus 8 mg of D-galactosamine) i.p. The RJX dose levels (in ml/kg) are indicated in parentheses. See text for discussion of results. These assays were performed on the lungs of mice that died after the LPS-GalN challenge. The lungs of the mice were harvested at the time of death which occurred within 24 h for all mice. The depicted lines represent the mean and standard deviation for the indicated parameters. In **(A)**, the score was graded according to a 5-point scale from 0 to 4 as follows: 0, l, 2, three and four represented no damage, mild damage, moderate damage, severe damage and very severe damage, respectively. Statistical significance between groups is shown by: ****p < 0.0001 compared as control group and, ##*p* < 0.01; ####*p* < 0.0001 compared as LPS/GaIN + NS group, Kruskal-Wallis test and Mann Whitney test. In **(B–F)**, ANOVA and Tukey’s post-hoc test were used for comparing the results among different treatment groups. Statistical significance between groups is shown by: ****p < 0.0001 compared as control group and, #*p* < 0.05; ##*p* < 0.01; ####*p* < 0.0001 compared as LPS/GaIN + NS group).

**FIGURE 6:**
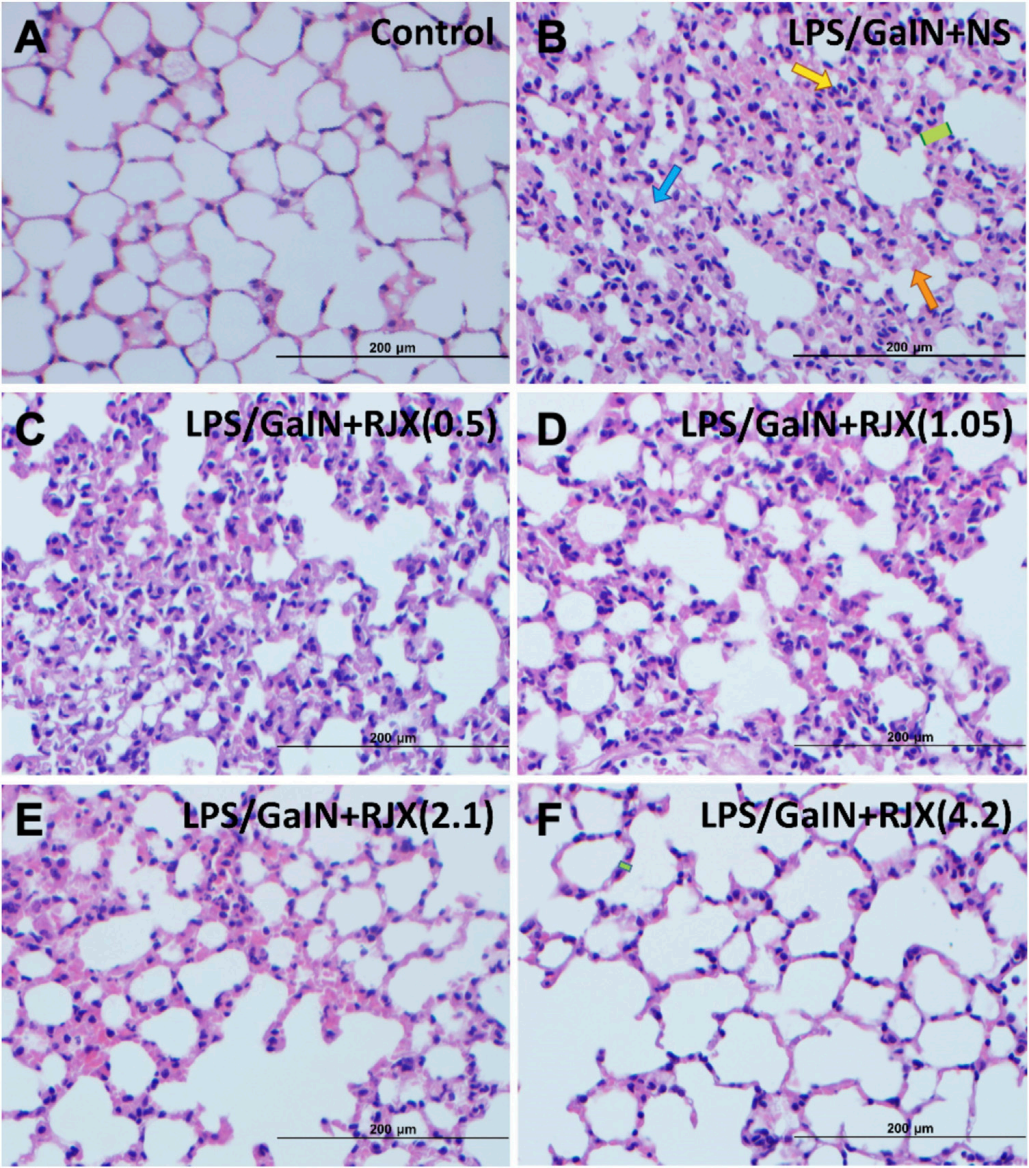
Rejuveinix (RJX) Prevents Acute Lung Injury and Inflammation in the Lipopolysaccharide-Galactosamine (LPS-GalN) Mouse Model of acute respiratory distress syndrome (ARDS) and Multi-organ Failure. Mice were treated with i.p injections of RJX (0.5, 1.05, 2.1, 4.2 ml/kg, 0.5 ml/mouse) or normal saline (NS) 2 h before and 2 h post-injection of LPS-GalN. Except for untreated mice (Control), each mouse received 0.5 ml of LPS-GalN (consisting of 100 ng of LPS plus 8 mg of D-galactosamine) i.p. The RJX dose levels (in ml/kg) are indicated in parentheses. Yellow arrow: inflammatory cell infiltration; Blue arrow: Exudate; Orange arrow: hemorrhage; Green block: thickness of alveolar wall. **Panel B**: Blue arrow: Exudate (see B1 of **Supplementary Figure S7** for an enlarged inset); Yellow arrow: inflammatory cell infiltration (see B2 of **Supplementary Figure S7** for an enlarged inset); Green block: thickness of alveolar wall (see B3 of **Supplementary Figure S7** for an enlarged inset). Orange arrow: hemorrhage (see B4 of **Supplementary Figure S7** for an enlarged inset); H&E ×400.

Western blot analysis of the lung tissue pro-inflammatory cytokine levels in LPS-GalN injected control mice (Group 2) vs. untreated healthy control mice not injected with LPS-GalN (Group 1) showed a highly statistically significant (*p* < 0.0001) 7.2 ± 0.1-fold elevation for interleukin 1 beta (IL-1β), 12.8 ± 0.1-fold elevation for IL-6, 3.3 ± 0.2-fold elevation for TNF-α, and 3.7 ± 0.8-fold elevation for transforming growth factor beta (TGF-β) (Figure 4). The lung MDA levels measuring lipid peroxidation were markedly elevated (6.5 ± 0.5 vs. 2.6 ± 0.4 nmol/g, *p* < 0.0001) and the levels of the antioxidant enzymes SOD (30.5 ± 1.2 U/mg vs. 80.4 ± 1.6 U/mg, *p* < 0.0001), CAT (19.9 ± 1.1 U/mg vs. 56.7 ± 1.4 U/mg, *p* < 0.0001), GSH-Px (54.2 ± 3.1 U/mg vs. 126.4 ± 4.1 U/mg, *p* < 0.0001), as well as ascorbic acid (54.5 ± 0.1 μg/g vs. 398.2 ± 0.1 μg/g, *p* < 0.0001) were markedly reduced in LPS-GalN treated mice (Group 2) when compared to healthy control mice (Group 1), consistent with severe oxidative stress in the lung tissue (Figure 5). Histopathological examination of H/E-stained lung tissues from LPS-GalN injected mice showed histological changes consistent with severe acute ALI, including alveolar hemorrhage, thickening of alveolar wall, edema/congestion, and leukocyte infiltration (Figures 5A, 6; Supplementary Figure S7). These changes were not observed in the lung tissues of mice in the control group that were not injected with LPS-GaIN.

RJX suppressed the levels of pro-inflammatory cytokines in the lung (Figure 4), decreased the lung MDA levels, and increased in a dose-dependent manner the reduced levels of the antioxidant enzymes SOD, CAT, GSH-Px and ascorbic acid (Figure 5). Notably, RJX attenuated the LPS-GalN induced ALI in a dose-dependent fashion, as evidenced by significantly less damage in the lungs of RJX-treated mice that were treated at the 2.1 and 4.2 ml/kg dose levels and were terminated healthy at 48 h. The ALI scores depicted in Figure 5A show a dose-dependent protective effect of RJX with a highly statistically significant reduction of the ALI scores at the highest two dose levels of RJX that were tested. The alveolar wall thickness as a measure of pulmonary edema was substantially decreased to near normal values at these dose levels of RJX (Group 5 and 6) (Figure 6; Supplementary Figure S7). Thus, RJX appeared to prevent the development of pulmonary edema in LPS-GalN challenged mice.

**FIGURE 7:**
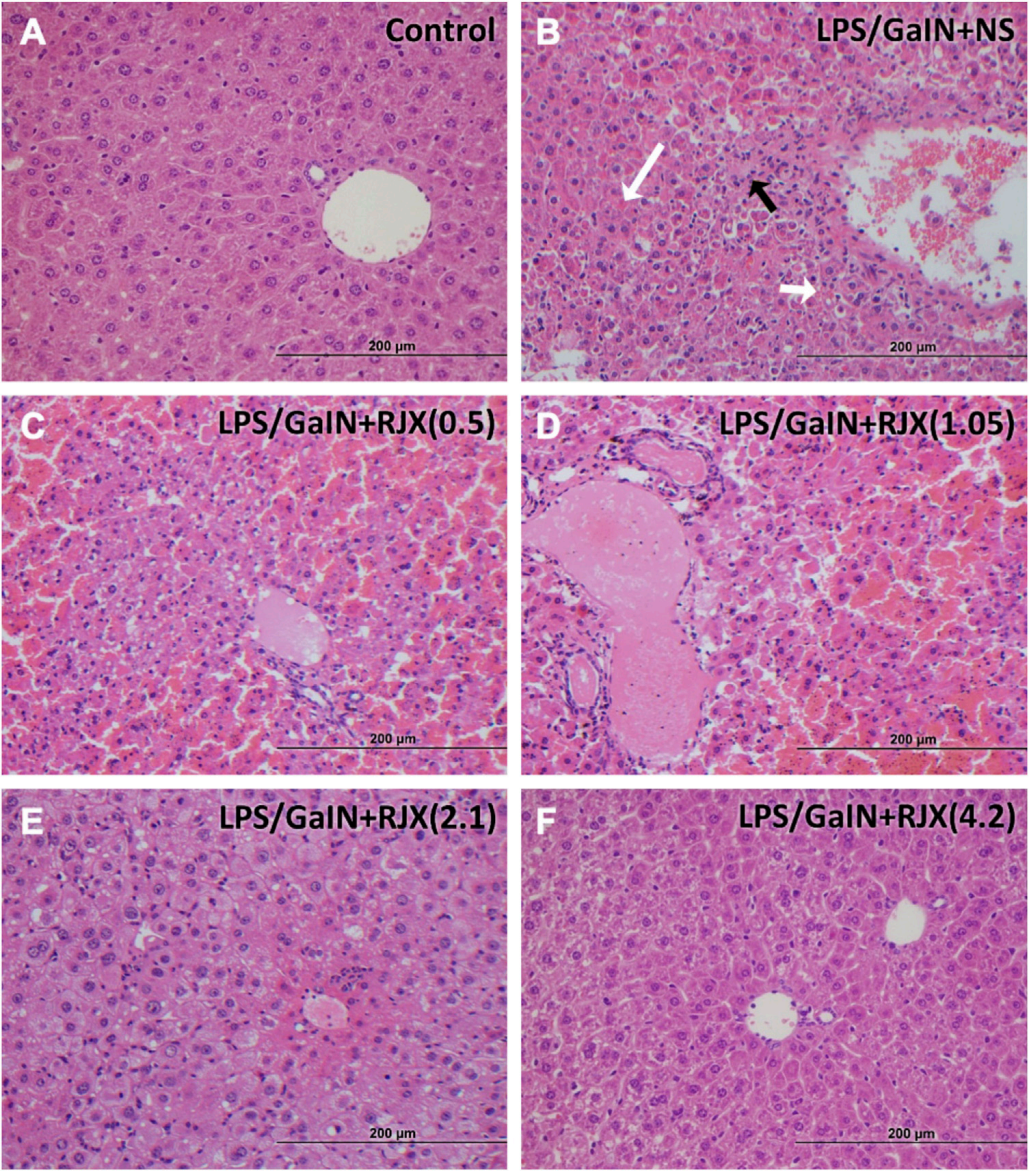
Rejuveinix (RJX) Prevents Acute Liver Injury and Inflammation in the Lipopolysaccharide-Galactosamine (LPS-GalN) Mouse Model of acute respiratory distress syndrome (ARDS) and Multi-organ Failure. Mice were treated with i.p injections of RJX (0.5, 1.05, 2.1, 4.2 ml/kg, 0.5 ml/mouse) or normal saline 2 h before and 2 h post-injection of LPS-GalN. Except for untreated mice (Control), each mouse received 0.5 ml of LPS-GalN (consisting of 100 ng of LPS plus 8 mg of D-galactosamine) i.p. The RJX dose levels (in ml/kg) are indicated in parentheses. **Panel A:** Treatment‐naive control mouse; **Panel B:** Mouse challenged with LPS‐GalN and treated with vehicle (placebo) (i.e., NS); **Panel C:** Mouse challenged with LPS‐GalN and treated with 0.5 ml/kg 6‐fold diluted RJX; **Panel D:** Mouse challenged with LPS‐GalN and treated with 1.05 ml/kg 6‐fold diluted RJX; **Panel E:** Mouse challenged with LPS‐GalN and treated with 2.1 ml/kg 6‐fold diluted RJX; **Panel D:** Mouse challenged with LPS‐GalN and treated with 4.2 ml/kg 6‐fold diluted RJX; **Panel B**: White arrow (long): necrosis (see B1 of **Supplementary Figure S11** for an enlarged inset); Black arrow: inflammatory cell infiltration (see B2 of **Supplementary Figure S11** for an enlarged inset); White arrow (short): hemorrhage (see B3 of **Supplementary Figure S11** for an enlarged inset). See text for a discussion of the findings. H&E ×400

LPS-GalN caused significant liver damage and hepatic dysfunction in mice with a marked elevation of the liver enzymes as well as total bilirubin when compared to the enzyme levels in untreated healthy control mice: alanine aminotransferase (1297.1 ± 106.8 vs. 28.3 ± 4.5; 46-fold elevation; *p* < 0.0001), aspartate aminotransferase (1756.0 ± 96.8 vs. 57.9 ± 10.3; 30-fold elevation; *p* < 0.0001), alkaline phosphatase (ALP) (491.2 ± 33.9 vs. 69.9 ± 6.7; 7-fold elevation; *p* < 0.0001), total bilirubin (TBIL) (1.9 ± 0.1 vs. 0.3 ± 0.1; six to three fold elevation; *p* < 0001) (Supplementary Figure S8). Western blot analysis of the liver tissue pro-inflammatory cytokine levels (IL-1β, TNF-α, IL-6, and TGF-β) in LPS-GalN injected control mice vs. untreated healthy control mice not injected with LPS-GalN showed a highly statistically significant (*p* < 0.0001) elevation for each cytokine (Supplementary Figure S9). Histopathologic examination of the livers from LPS-GalN treated mice showed massive generalized hepatocyte vacuolation and necrosis corresponding to >25% necrosis and destruction of the liver parenchyma, severe lobular inflammation involving >50% of the liver parenchyma, and severe portal inflammation involving more than >50% of portal tracts (Figure 7; Supplementary Figures S10, S11). The liver MDA levels measuring lipid peroxidation were markedly elevated and the levels of the antioxidant enzymes SOD, CAT, GSH-Px, as well as ascorbic acid were markedly reduced in LPS-GalN treated mice consistent with severe oxidative stress (Supplementary Figure S10). RJX suppressed the levels of pro-inflammatory cytokines in the liver (Supplementary Figure S9) as well as the LPS-GalN induced liver injury and inflammation noted in histological examination (Supplementary Figures S10A, S11; Figure 7) in a dose-dependent fashion. Furthermore, RJX decreased the liver MDA levels and normalized in a dose-dependent manner the reduced levels of the antioxidant enzymes SOD, CAT, and GSH-Px as well as ascorbic acid. Consequently, RJX reduced the mortality rate of LPS-GalN associated systemic inflammatory response, and RJX-treated mice that died following the LPS-GalN challenge had significantly lower, albeit still abnormal, levels for liver enzymes and TBIL (Supplementary Figure S8).

### *In Vivo* Protective Activity of Delayed-Onset Rejuveinix Treatments in the Lipopolysaccharide-Galactosamine Model of Acute Respiratory Distress Syndrome and Multiorgan Failure

**FIGURE 8:**
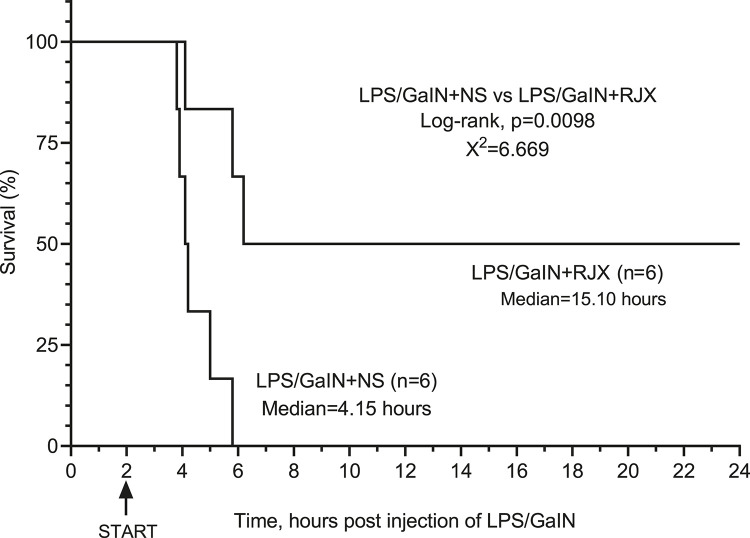
*In Vivo* Protective Activity of Delayed-Onset Rejuveinix (RJX) Treatments in the Lipopolysaccharide-Galactosamine (LPS-GalN) Model of acute respiratory distress syndrome (ARDS) and Multiorgan Failure. Groups of 6 BALB/C mice were treated with i.p. injections of 6-fold diluted RJX (4.2 ml/kg, 0.5 ml/mouse) or vehicle [normal saline (NS)] 2 and 3 h post-injection of LPS-GalN. Each mouse received 0.5 ml of LPS-GalN (consisting of 100 ng of LPS plus 8 mg of Dgalactosamine) i.p. Percent (%) survival for each treatment group is shown as a function of time after the LPS-GalN challenge. Depicted are the survival curves for each group along with the median survival times and the log-rank *p*-value for the comparison of LPS-GalN + RJX group vs. the LPS/GaIN + NS group.

We next sought to determine if RJX could also improve the survival outcome of LPS-GalN challenged mice if the treatment is delayed until after the onset of the inflammatory cytokine response. As shown in Supplementary Figure S12, the serum IL-6 and TNF-α levels were markedly elevated in 6 of 6 control mice that were terminated at 2 h after the i.p. LPS-GalN injection when compared to six untreated control mice. At the time of their termination, these LPS-GalN injected mice also had marked lipid peroxidation in the lungs as evidenced by dramatically elevated MDA levels in the lungs. Hence, at 2 h post LPS-GalN, mice had evidence of a significant inflammatory response and oxidative stress in the lungs. In our proof of concept experiment aimed at evaluating RJX as a potential treatment for sepsis, we started treatments of mice with NS vs. RJX at this time 2 h time point. Control mice (N = 6) treated with two injections of vehicle (NS) at 2 and 3 h, respectively, post LPS-GalN challenge all rapidly died within 4 h at a median survival time of 2.15 h after the first injection of NS and 4.15 h after the LPS-GalN challenge. At the time of death, their serum IL-6 and TNF-α levels as well lung tissue MDA levels were drastically elevated and even higher than the levels in untreated control mice that were terminated at 2 h post LPS-GalN injection (Supplementary Figure S12). Notably, treatment of mice with 6-fold diluted 4.2 ml/kg RJX at 2 and 3 h post LPS-GalN injection resulted in improved survival outcome with 3 of 6 mice remaining alive at 24 h post LPS-GalN injection (Figure 8). The median survival time of these mice was markedly longer than the median survival of NS-treated control mice (15.1 vs. 4.15 h, *p* = 0.0098) (Figure 8). The serum levels of inflammatory cytokines IL-6 and TNF-α as well as lung MDA levels at the time of death (N = 3) or lective termination at 24 h (N = 3) were lower than the baseline levels in mice sacrificed at the 2 h post LPS-GalN challenge time point when RJX and NS treatments were initiated (Supplementary Figure S12). These results provide direct evidence that low dose RJX is capable of preventing fatal cytokine storm and reducing the mortality rate of LPS/GalN-induced systemic inflammation when the treatment is delayed until after the onset of a systemic inflammatory cytokine response and oxidative stress in the lungs.

## Discussion

In the phase 1 clinical study of RJX in healthy volunteers, no participant developed SAEs or Grade 3-Grade 4 AEs or discontinued participation early. Thus, RJX showed a very favorable clinical safety profile. Following both single and multiple-dose IV infusion of RJX, all five API evaluated (ascorbic acid, cyanocobalamin, magnesium sulfate, niacinamide, and thiamine) rapidly appeared in the systemic circulation. There was no clinically significant accumulation of these compounds following once-daily IV infusion for 7 days, as demonstrated by the PK profiles on Day 7 that were overall similar to those observed on Day 1. Despite its tolerability, the 0.759 ml/kg dose level (viz.: dose level in Cohort 5 of Part 1 and Cohort 3 in Part 2 of the phase 1 study) was not selected for further evaluation in future studies, including the projected phase 1-2 COVID-19 study due to the model fitted projections in the cardiodynamic evaluations. Specifically, the results of the concentration-QTc analysis informed our decision to select the 0.5 ml/kg dose level and not the 0.759 ml/kg dose level for the projected phase 1-2 COVID-19 trial. In all concentration-QTc analyses, the upper bound of the 90% CI of the QTc effect was greater than 10 ms in the 0.759 ml/kg group for all component concentrations, except in the modeling of thiamine and magnesium alone for the SAD group. An effect exceeding 10 msec could be excluded at plasma concentrations of ascorbic acid, cyanocobalamin, magnesium, niacinamide, and thiamine up to 166 mg/L, 362 μg/L, 4.0 mg/dl, 7570 μg/L, and 4890 μg/L, respectively. Such plasma concentrations were only observed at the 0.759 ml/kg dose level and not at 0.5 ml/kg dose level. Therefore, the modeling study indicated that a potential QTc interval prolongation of >10 msec could not be excluded at the 0.759 ml/kg dose level. Based on these predictions, a dose level of 0.500 ml/kg for which the above mentioned concentrations were not observed or predicted was selected as the tentative dose level for further clinical development of RJX in future studies. While we do not anticipate any clinically meaningful effect on the QTc of the COVID-19 patients who will not receive RJX at a dose exceeding 0.500 ml/kg, risk mitigation measures will be used to monitor patients for QTc prolongation. Serum potassium and magnesium levels will be required to be within normal limits, prior to RJX infusion and the protocol advises that medications with potential for QTc prolongation should ideally not be started within 24 h of RJX administration. If a subject has a QTcF interval >500 msec on an ECG machine printout, it is recommended that the subject be placed on a cardiac monitor while cardiology consultation and review of the ECG is obtained. If the QTcF >500 msec is confirmed, the treating physician will follow the recommendations of the consulting cardiologist. Furthermore, the exclusion criteria include subjects with a history of congenital long QT syndrome or of Torsades de pointes; subjects with bradycardia (<60 bpm), heart block (excluding first degree block, being PR interval prolongation only); subjects with any of the following findings on ECG: QTc interval >470 msec in women OR >450 msec in men; subjects requiring any drugs known to prolong the QTc interval, including antiarrhythmic medications.

Carver and Jones, 2020; Kuderer et al., 2020; Lian et al., 2020; Uckun, 2020a; Uckun, 2020b; Wu et al., 2020; Zheng et al., 2020; Zhou et al., 2020. Notably, patients ≥65 years of age had a 3.3 to 3.6-fold increased risk of developing ARDS than patients <65 years of age (Bi et al., 2020; Uckun, 2020a; Wu et al., 2020). Importantly, once patients with ≥65 years of age develop ARDS, their risk of death is 6.2-fold higher than the risk of younger patients. Likewise, the risk of developing ARDS for patients ≥60 years of age was reportedly 3.1-fold higher than the risk for younger patients (Lian et al., 2020). Whereas the reported median age for non-survivors was 69 years, the median age for survivors was 52 years (*p* < 0.0001) (Zhou et al., 2020). A recent review of 17 clinical studies by Carver and Jones also confirmed that older age, diabetes, higher serum levels of inflammatory markers (LDH, CRP), and coagulopathy with higher serum D-dimer levels are associated with a higher risk for ARDS (Carver and Jones, 2020). Thus, an older patient with high-risk COVID-19 is in urgent need for treatment platforms capable of preventing the disease progression and/or reducing the case mortality rate in the high-risk COVID-19 patients by stopping or reversing the inflammatory process that causes ARDS and multi-organ failure after the development of pulmonary inflammation. Therefore, it will be important to evaluate the safety and tolerability profile of RJX in older patients with high-risk COVID-19 who are in urgent need for therapeutic innovations. In our projected COVID-19 study, Cohort 1 will include older patients with at least one additional comorbidity (diabetes or hypertension).

TGF-β genes are up-regulated in COVID-19 (Uckun, 2020c; Uckun and Trieu, 2020b; Xiong et al., 2020) and Stafford et al. postulated that TGF-β is a crucial molecule, along with IL-6 and TNF-α in the pathogenesis of severe COVID-19 (Stafford et al., 2020). TGF-β has been implicated as an important pro-inflammatory cytokine in the pathophysiology of acute lung injury and ARDS (Pittet et al., 2001; Fahy et al., 2003; Frank and et al., 2003; Willis and et al., 2003; Jenkins and et al., 2006; Frank and Matthay, 2014). A recent study by Peters et al. demonstrated that TGF-β profoundly impacts alveolar ion and fluid transport through regulating the epithelial sodium channel (ENaC) activity and trafficking via a TGF-βR1-dedicated signaling pathway (Peters et al., 2013). The upregulation of TGF-β in ARDS causes an ENaC trafficking defect with a marked reduction in the cell-surface abundance of ENaC on lung epithelial cells, thereby rapidly and substantially impairing the alveolar fluid reabsorption in ARDS patients and contributing to the persistence of their pulmonary edema (Peters et al., 2013). Notably, a significant negative correlation existed between TGF-β levels in bronchoalveolar lavage fluid samples and ventilator-free/ICU-free days in ARDS patients. Furthermore, lower TGF-β levels correlated with better survival outcomes indicating that patients with higher TGF-β levels may have higher and faster case mortality (Budinger et al., 2005). TGF-β also stimulates platelet activation and formation of thrombi (Woolum et al., 2018) and may contribute to the coagulopathy and pulmonary blood clot formation in COVID-19 associated ARDS (Fox et al., 2020). A soluble recombinant TGF-β receptor protein capable of sequestering TGF-β has effectively attenuated the severity of pulmonary edema in experimental models of ARDS (Uckun and Trieu, 2020a). Notably, our results demonstrate that RJX treatments result in a marked reduction of not only IL-6 and TNF-α, but also in TGF-β levels in the lungs as well as liver of mice challenged with LPS-GalN in a dose-dependent fashion. We postulate that the RJX-mediated suppression of TGF-β expression in the lungs will accelerate the resolution of pulmonary inflammation of COVID-19 patients by reducing the contributions of TGF-β to ARDS and its complications. TGF-β is also involved in the pathogenesis of lung tissue remodeling and lung fibrosis that follows ARDS. Specifically, TGF-β contributes to the development of lung fibrosis by stimulating the proliferation/differentiation of lung fibroblasts, accumulation of collagen and other extracellular matrix proteins in the pulmonary interstitial and alveolar space, leading to the occurrence and development of pulmonary fibrosis (Shimbori et al., 2016; Nithiananthan et al., 2017). Therefore, inhibition of the TGF-β signaling pathway by RJX similarly has the potential to prevent the development of pulmonary fibrosis following ARDS and improve the pulmonary healing process (Hu and Huang, 2019).

IL-6 is a pro-inflammatory cytokine that contributes to the development, progression, and severity of CRS as well as its complications, including disseminated intravascular coagulopathy (DIC) and multi-organ failure (Chen et al., 2020; Huang et al., 2020; Uckun, 2020b; Young et al., 2019; Zumla et al., 2020). It is the main signature cytokine implicated in COVID-19 associated CRS and ARDS (Uckun et al., 2020). Anti-IL6 receptor antibodies tocilizumab (ClinicalTrials.gov Identifiers: NCT04317092, NCT04306705, NCT04310228) and sarilumab (ClinicalTrials.gov Identifier: NCT04315298) are being assessed for their therapeutic activity in COVID-19 patients in both open-label as well as randomized clinical trials. TNF-α is another pro-inflammatory cytokine implicated in the pathophysiology of CRS and ARDS in COVID-19 (Chen et al., 2020; Huang et al., 2020; Uckun, 2020b; Young et al., 2019; Zumla et al., 2020). RJX treatments appeared to have a statistically significant and dose-dependent lowering effect on the levels of IL-6 as well as TNF-α in the blood, lung tissue, and liver. We believe the observed robust effect of RJX on serum and tissue cytokine levels significantly contributed to its ability to reduce the ALI, pulmonary inflammation and prevent the development of pulmonary edema, as well as its ability to reduce the liver damage that was accompanied by destruction of the liver parenchyma, severe hepatic inflammation, massive hepatic necrosis, and hepatic dysfunction in LPS-GalN challenged mice. Together these favorable effects ultimately translated into improved survival outcomes of RJX-treated mice.

Some pediatric patients with COVID-19 have developed Kawasaki disease (KD), a TGF-β mediated systemic immunoglobulin A (IgA) vasculitis causing cardiac complications, including acute myocardial infarction, myocarditis, left ventricular dysfunction, pericardial effusion and mitral valve insufficiency as well as a multi-system inflammatory syndrome (Jones et al., 2020; Harahsheh et al., 2020; Lee et al., 2015; McCrindle et al., 2017; Shimizu et al., 2011). Reminiscent of the cytokine profiles of COVID-19 patients with severe disease, acute KD is characterized by high levels of IL-6, TNF-α, and TGF-β (McCrindle et al., 2017). Based on the aforementioned role of TGF-β in KD and the ability of RJX to suppress the production of IL-6, TNF-α, and TGF-β, we hypothesize that RJX will emerge as a clinically useful adjunct to the IV immunoglobulin therapy and best supportive care in pediatric COVID-19 patients who develop KD.

Hydroxyl radicals are produced from superoxide anion radicals and/or hydrogen peroxide. Both superoxide radicals and hydroxyl radicals can individually act on lipid membranes to promote the formation of lipid radicals, which in the presence of oxygen are capable of generating lipid peroxy radicals that can cause damaging lipid peroxidation of cell membranes (Josephs et al., 2000; Dong and Yuan, 2018; Ighodaro and Akinloye, 2018). Lipid peroxides contribute to liver injury by increasing production of TNF-α, increasing the influx of inflammatory cells and neutrophils, impairing DNA and protein synthesis, and depleting protective cellular antioxidants such as glutathione. SOD, CAT, and GSH-Px are three pivotal anti-oxidant defense enzymes and their levels are altered by the level of oxidative stress (Michael et al., 2000; Dong and Yuan, 2018; Ighodaro and Akinloye, 2018). SOD converts superoxide anion radicals into hydrogen peroxide and oxygen, whereas CAT catalyzes the production of water and oxygen from hydrogen peroxide. CAT is absent in the mitochondria, hence the reduction of H_2_O_2_ to water the reduction of lipid peroxides to their corresponding alcohols is carried out by GSH-Px instead. GSH-Px plays a more crucial role in inhibiting the lipid peroxidation process and therefore is essential for protecting cells from oxidative stress (Dong and Yuan, 2018). In accordance with the published role of oxidative stress in LPS-GalN induced lung and liver injury (Dong and Yuan, 2018), the lung/liver MDA levels, reflecting increased lipid peroxidation, were markedly elevated, and the levels of the antioxidant enzymes SOD, CAT, GSH-Px, as well as ascorbic acid were reduced in LPS-GalN treated mice consistent with severe oxidative stress. RJX suppressed the levels of pro-inflammatory cytokines in the lung/liver, decreased the lung/liver MDA levels, and increased in a dose-dependent manner the reduced levels of the antioxidant enzymes SOD, CAT, GSH-Px and ascorbic acid. These results establish RJX as a potent antioxidant with strong anti-inflammatory activity. A Phase 1‐2 study is designed to obtain the clinical proof of concept that RJX could favorably alter the clinical course and reduce the mortality rate of high-risk COVID-19 (Cabala et al., 2020).

RJX (Van Wyk et al.) is a formulation of several vitamins, including ascorbic acid (Vitamin C), cyanocobalamin (Vitamin B12), thiamine hydrochloride (Vitamin B1), riboflavin 5′ phosphate (Vitamin B2), niacinamide (Vitamin B3), pyridoxine hydrochloride (Vitamin B6), calcium D-pantothenate, and magnesium sulfate representing components with reported, albeit controversial, protective activity in animal models of septic shock and ARDS as well as some of the clinical studies in septic patients (Uckun et al., 2020). Increased lactate levels contribute to the enhanced mortality of septic patients (Wang et al., 2014). Two of the RJX ingredients, thiamine (Woolum et al., 2018) and magnesium sulfate (Noormandi et al., 2020), accelerate the lactate clearance, which has been shown to improve survival outcomes (Pan et al., 2019). While some preclinical, translational, early-stage, as well as late-stage clinical studies, have yielded promising positive data regarding the clinical impact potential of ascorbic acid, thiamine, riboflavin, niacinamide, and pyridoxine (Vit. B6) in the prevention and treatment of CRS, coagulopathy, ALI, acute kidney injury (AKI), ARDS, and MODS in the context of sepsis (Li, 2018; Hager et al., 2019; Putzu et al., 2019; Iglesias et al., 2020; Obi et al., 2020; Noormandi et al., 2020; Shan et al., 2020), other studies have not shown any meaningful activity (Fujii et al., 2020 Jan 17; Moskowitz et al., 2020). For example, Moskowitz et al. recently reported the results of a 205-patient, randomized blinded, multicenter study of ascorbic acid, thiamine and steroids in patients with septic shock that was performed at 14 centers in the United States Patients were randomly assigned to receive parenteral ascorbic acid (1,500 mg), hydrocortisone (50 mg), and thiamine (100 mg) every 6 h for 4 days (n = 103) or placebo in matching volumes at the same time points (n = 102) (ClinicalTrials.gov Identifier: NCT03389555) (Moskowitz et al., 2020). The primary endpoint was change in the Sequential Organ Failure Assessment (SOFA) score (range, 0–24; 0 = best) between enrollment and 72 h. There was no statistically significant interaction between time and treatment group with regard to SOFA score over the 72 h after enrollment. Hence, the combination of ascorbic acid, corticosteroids, and thiamine, compared with placebo, did not result in a statistically significant reduction in SOFA score during the first 72 h after enrollment. Similarly, Fujii et al. reported that treatment with intravenous ascorbic acid hydrocortisone, and thiamine does not lead to a more rapid resolution of septic shock compared with intravenous hydrocortisone alone based on the results of a randomized trial in 216 patients with septic shock (ClinicalTrials.gov Identifier: NCT03333278) (Fujii et al., 2020). In contrast to these studies, Iglesias et al. reported that the combination of IV ascorbic acid, thiamine, and hydrocortisone significantly reduced the time to resolution of shock based on another randomized study (ClinicalTrials.gov Identifier: NCT03422159) (Iglesias et al., 2020). Likewise, Byerly et al. reported that ascorbic acid plus thiamine is associated with increased survival in sepsis based on the results of 11,330 patients with sepsis and elevated lactate levels (Byerly et al., 2020). After controlling for confounding factors, ascorbic acid [adjusted odds ratio (AOR), 0.69 (0.50–0.95)] and thiamine [AOR, 0.71 (0.55–0.93)] were independently associated with survival (Byerly et al., 2020). Additional studies are needed to confirm these findings and assess any potential benefit from this treatment. RJX has no steroids and it contains niacinamide, pyridoxine, cyanocobalamin and Mg-sulfate in addition to ascorbic acid and thiamine. The clinical potential of RJX will be examined in COVID-19 patients with an emphasis on prevention of ARDS and multiorgan failure in COVID-19 patients at high risk for fatal viral sepsis rather than treatment of septic shock.

The selection of the tentative phase 1-2 dose level for further evaluation was not based on a validated pharmacodynamic marker for the COVID-19 indication, but rather based on its safety profile in the phase 1 clinical study in healthy volunteers (Protocol No. RPI003; ClinicalTrials.gov Identifier: NCT03680105) with no evidence of Grade 3 or 4 AEs, no SAEs and no deaths observed at this dose level. The dose level selected for the COVID-19 study is > 1.6-fold lower than the NOAEL in rats and >5.4-fold lower than the NOAEL in dogs, and it is one dose level lower than the highest dose tested in the phase 1 study. In the projected phase 1 - 2 clinical study for COVID-19 patients, an open label Safety Lead in (Part 1) will be included aimed at further assessing its tolerability and safety in COVID-19 patients. The oxidative stress in peripheral blood mononuclear cells obtained from consenting COVID-19 patients before and after RJX infusion will be evaluated using flow cytometry with the standard DCF staining method. The effects of RJX on inflammatory biomarkers will also be evaluated within the confines of an open-label Part 1 portion of the phase 1-2 study. The efficacy of RJX will be determined in the randomized, double blind, placebo-controlled Part 2 of the study following the review of the Part 1 data by an independent Date and Safety Monitoring Board.

Disease progression from mild-moderate to severe-critical was systematically analyzed by Bi et al. (2020). Among the 417 patients who were classified as mild or moderate at the time of initial assessment, 21.6% (90/417) progressed to the severe stage. Those who progressed to the severe stage progressed within on average 9.5 days (95%CI: 8.7–10.3) after symptom onset. They developed ARDS on average 11.0 days (95% CI: 9.7–12.3) after symptom onset. Patients with certain co-morbidities such as diabetes and hypertension at baseline and/or laboratory parameters such as high concentration of CRP, LDH, and high concentration of D-dimer formed a higher risk subgroup with a higher likelihood of progression to ARDS. In this high-risk group, Bi et al. estimated that 43% (95%CI: 35–52%) cases (i.e., twice as many as the 21.6% for the total population of mild-moderate patients) became severe and 9% (95%CI: 4–14%) required ICU admission within 14 days from symptom onset (Bi et al., 2020). More information has recently become available regarding the patient characteristics, disease progression, and clinical outcomes for the COVID-19 patient population in the United States Gold et al. (2020) reported the results on 305 patients hospitalized in Georgia in the month of March 2020. 37.6% of the 117 patients in the high-risk age category ≥65 years required high-flow oxygen, or noninvasive ventilation (NIV), whereas 41.0% developed ARDS and required mechanical ventilation (MV). By comparison, 25.6% of the total population required high-flow oxygen or NIV, and 30.6% developed ARDS. In summary, approximately 20% of COVID-19 patients with mild-moderate disease progress to severe-critical disease and this percentage is approximately 40% for the high-risk subgroup ≥65 years of age with co-morbidities or laboratory parameters indicative of systemic inflammation such as high levels of CRP, LDH, and ferritin or a dysfunction of the coagulation system as evidenced by elevated D-dimer levels (Uckun, 2020a). We hypothesize that RJX treatments will reduce the progression for the high-risk subgroup in Cohort 1 from 40% down to 20%.

Our preliminary data in the mouse model of LPS-GalN induced sepsis, ARDS, and multi-organ failure indicate that RJX also shows potential for treatment of COVID-19 with evolving viral sepsis. Cohort 2 of the proposed COVID-19 study will be hospitalized COVID-19 patients with hypoxemia without ARDS receiving either NIPPV or high-flow oxygen. In the randomized Part 2 of the study, these Cohort 2 patients will be treated with Placebo + Standard of care or RJX + Standard of care. We hypothesize that RJX will contribute to a faster resolution of the respiratory failure and a reduced case mortality rate.

## Conclusion

RJX could favorably affect the clinical course of high-risk COVID-19 and reduce its case mortality rate. RJX is anticipated to shorten the time to resolution of ARDS by eliminating the contributions pro-inflammatory cytokines IL-6, TNF-α and TGF-β to the systemic and pulmonary inflammatory process.

## Data Availability Statement

All datasets presented in this study are included in the article/Supplemental Material.

## Ethics Statement

The studies involving human participants were reviewed and approved by IntegReview IRB (3815 S. Capital of Texas Hwy, Suite 320, Austin, TX 78704, IRB Registration Numbers: IRB IRB00008463, IRB00003657, IRB00004920, IRB00001035, IRB00006075). The patients/participants provided their written informed consent to participate in this study. The animal study was reviewed and approved by Animal Care and Use Committee of Firat University, Firat University.

## Author Contributions

Each author (FU, JC, CO, JP, NP, HW, IH, MV, KS) has made significant and substantive contributions to the study, reviewed and revised the manuscript, provided final approval for submission of the final version. No medical writer was involved. FU conceived the study, designed the evaluations reported in this paper, directed the data compilation and analysis, analyzed the data, and prepared the initial draft of the manuscript. Each author had access to the source data used in the analyses IO. performed the necropsies and histopathologic examinations on mice. KS and CO performed animal experiments and the statistical analysis of the laboratory data.

## Funding

This work was funded by institutional funds of Reven Pharmaceuticals.

## Conflicts of Interest

FU, JC, JP, NP, HW, and MV are employees and minority shareholders of Reven Pharmaceuticals. FU is an employee of Ares Pharmaceuticals.

The remaining authors declare that the research was conducted in the absence of any commercial or financial relationships that could be construed as a potential conflict of interest.
