# Supplementary material for: Rejuveinix Shows a Favorable Clinical Safety Profile in Human Subjects and Exhibits Potent Preclinical Protective Activity in the Lipopolysaccharide-Galactosamine Mouse Model of Acute Respiratory Distress Syndrome and Multi‐Organ Failure
Source: Front Pharmacol. 2020 Nov 10;11:594321. doi: 10.3389/fphar.2020.594321 (PMC7683794; doi:10.3389/fphar.2020.594321)
Supplement: Supplementary file 1 [file DataSheet1_v1.PDF]

# Supplemental Material

## REJUVEINIX SHOWS A FAVORABLE CLINICAL SAFETY PROFILE IN HUMAN SUBJECTS AND EXHIBITS POTENT PRECLINICAL PROTECTIVE ACTIVITY IN THE LPS-GALN MOUSE MODEL OF ACUTE RESPIRATORY DISTRESS SYNDROME (ARDS)

### Supplemental Methods

*Ethics Statement and Approval of the Clinical Study*

This study was performed by the clinical research organization ICON Clinical Research, LLC, Gaithersburg, MD 20878 USA in compliance with the ICH(E6) good clinical practice (GCP) guidelines. Healthy volunteer subjects were treated at the clinical facility ICON Early Phase Services, LLC, 8307 Gault Lane, San Antonio, TX 78209 USA and in adherence to the ethical principles based on the Declaration of Helsinki, GCPs, ICH guidelines, and the applicable national and local laws and regulatory requirements. (Date of first enrollment: 8/27/2018 – Date of last completed: 2/16/2019). The study was approved by the IntegReview IRB (3815 S. Capital of Texas Hwy, Suite 320, Austin, TX 78704, IRB Registration Numbers: IRB IRB00008463, IRB00003657, IRB00004920, IRB00001035, IRB00006075). Each patient provided written informed consent. A safety review committee (SRC) reviewed the safety data for each cohort prior to escalation to the next cohort. The SRC was composed of the investigator, ICON's medical monitor, an independent medical monitor (neurologist), and other members of the investigational team as deemed appropriate. All members of the SRC remained blinded for review of the safety and available PK data, as there was no need to unblind due to safety concerns. Adverse events were closely monitored for possible neurological adverse reactions. A qualified neurologist was available at the SRC meetings to adjudicate any observed neurological events and subsequent actions associated with further dosing if required. The SRC reviewed the safety data for a cohort prior to escalation to the next cohort.

*Study Design, Execution of the Clinical Trial, and Patient Characteristics*

Part 1 was a SAD escalation study in 52 participants as 6 cohorts. The dose levels of RJX and treatments are detailed in **Table S2**. Part 2 was a MAD escalation study in 24 participants as 3 cohorts of 8 participants (6 RJX:2 placebo). The dose levels of RJX and treatments are detailed in **Table S2**. 52 participants in Part 1 and 24 participants in Part 2 were planned and analyzed. The study design of the Phase 1 clinical trial was as follows:

Part 1: Part 1 was a single ascending dose escalation study in 52 participants, 6 cohorts in total. Cohorts 1 to 5 included 8 participants per cohort (6 RJX: 2 placebo) aged 18 to 50 years, inclusive. Cohort 6 investigated an older population of 12 participants (9 RJX: 3 placebo) aged 51 to 70 years, inclusive. The assignment to either RJX or placebo was blinded to the participants, investigators, and staff at the study site. Part 1 consisted of screening (Days -21 to -1), treatment, and follow-up periods. Participants who met eligibility criteria were admitted to the study site on Day -1, when continued eligibility was assessed. Cohort 1 included the initial dosing of a sentinel group (1 RJX and 1 placebo). The remaining 6 participants in Cohort 1 (5 RJX: 1 placebo) were dosed when, in the opinion of the investigator, there were no significant safety concerns identified in the sentinel participants within the first 24 hours after administration of the dose (RJX or placebo). Participants received a single dose of RJX as an IV infusion on a single occasion on Day 1 as follows:

Cohort 1: 0.024 mL/kg RJX (number [n] = 6)/placebo (n =
2) Cohort 2: 0.076 mL/kg RJX (n = 6)/placebo (n = 2)
Cohort 3: 0.240 mL/kg RJX (n = 6)/placebo (n = 2)
Cohort 4: 0.500 mL/kg RJX (n = 6)/placebo (n = 2)
Cohort 5: 0.759 mL/kg RJX (n = 6)/placebo (n = 2)
Cohort 6: 0.500 mL/kg RJX (n = 9)/placebo (n = 3)

Part 2: Part 2 of the study was a multiple ascending dose (MAD) escalation study in 24 participants, 3 cohorts of 8 participants (6 RJX: 2 placebo). The MAD arm of the study commenced in parallel with Cohort 6 of Part 1 following completion and review of the safety findings for Cohorts 1 to 5 in Part 1. Participants were randomly assigned to receive 1 of 3 doses of RJX or placebo (6 RJX: 2 placebo) every day for 7 days as follows:

Cohort 1: 0.240 mL/kg RJX (n = 6)/placebo (n = 2)
Cohort 2: 0.500 mL/kg RJX (n = 6)/placebo (n = 2)
Cohort 3: 0.759 mL/kg RJX (n = 6)/placebo (n = 2)

Dose Escalation in Parts 1 and 2: A safety review committee (SRC) reviewed the safety data for each cohort prior to escalation to the next cohort. In Part 1, the SRC review of data from Cohorts 1 and 2 consisted of review of blinded safety data (adverse events [AEs], vital signs, ECGs, and laboratory values) from a completed cohort after the completion of Day 2 and prior to dose escalation. The SRC review prior to dosing of Cohorts 3, 4, and 5 included review of both blinded safety data and blinded cumulative PK data prior to dose escalation.

Part 2, Cohort 1 commenced upon SRC cumulative review of data from Part 1, Cohort 1 to Cohort 5. Part 2, Cohort 1, ran concurrently with Part 1, Cohort 6. In Part 2, the SRC reviewed blinded safety and PK data for the 7 days of treatment for each cohort prior to dose escalation. To attempt to efficiently identify the optimal therapeutic doses for use in future clinical trials, a flexible scheme of dose escalation based on ongoing study data was used.

Participants were randomly assigned to receive either RJX or placebo. A computer-generated randomization schedule and kit list were prepared prior to the start of the study. The investigational product RJX was prepared and dispensed in accordance with the randomization schedule and kit list. Detailed drug preparation and administration instructions were provided to the clinic. The master randomization schedule and kit list were made of randomly permuted blocks of appropriate sizes, as determined by the unblinded study team member producing the schedules. The schedules were generated through the Statistical Analysis System (SAS) software, version 9.3. After signing the informed consent form (ICF), each participant was given a unique screening number according to the screening order. Prior to dosing, each participant was allocated a randomization number according to the chronological order of inclusion in the study. This number corresponded to a treatment (RJX or placebo) as specified on the predetermined randomization schedule. Confirmation of the allocated randomization number was documented in the drug accountability records and recorded in the eCRF. Both the screening and randomization numbers were used to identify the participant throughout the study and on all study-related documentation. If a participant was replaced, the replacement would have taken the same treatment assignment as the original participant to ensure that the treatment groups stayed balanced.

Serial ECGs were recorded and paired with PK determinations to allow for concentration-QT analysis. Safety parameters included AEs, physical and neurological examinations, clinical laboratory evaluations, vital sign measurements, 12-lead safety ECGs, concomitant medications, and an evaluation of cardiac endpoints. Adverse events (AEs) were listed and summarized by treatment and visit/timepoint using descriptive methods. Clinical laboratory safety parameters, vital sign measurements, and 12-lead safety ECG parameters were listed and summarized using descriptive statistics for each treatment, timepoint, and changes from baseline. The AEs of special interest included neurological and infusion-site-related AEs.The PK parameters of the RJX components were calculated, whenever possible, from the concentrations versus time profiles using non-compartmental analysis (NCA), with Phoenix® WinNonlin® version 8.0. For a cardio-dynamic ECG assessment, continuous recordings were performed on Day 1 in Part 1 and on Days 1 and 7 in Part 2. All Holter/ECG data were collected using M12R continuous 12-lead digital recorders and the M12A Enterprise Holter System Client (Global Instrumentation, LLC, Manlius, New York, USA). The equipment was supplied and supported by ERT. A cardiac report was prepared by ERT for the evaluation of cardiac endpoints from the cardiodynamic analysis.

*Demographic and Other Baseline Patient Characteristics*

Baseline patient characteristics are summarized in **Table S3**. In Part 1, participant ages were similar for younger Cohorts 1 through 5 (range 19 to 44 years of age) while the older Cohort 6 had ages ranging from 51 to 61 years of age (**Table S3**). The majority of participants in most cohorts and overall were generally White, while the majority of participants who received placebo were Black or African American (**Table S3**). In Part 2, participant ages were similar for participants in all RJX cohorts (Cohorts 1 through 3, range 20 to 50 years of age). The ages of participants who received placebo (2 per cohort in Cohorts 1 through 3) ranged from 22 to 48 years of age. Gender for participants who received placebo and Cohorts 2 and 3 was split so that there was a minimum of 33.3% of each gender in each cohort; all participants in Cohort 1 were male. Ethnicity within cohorts and for participants who received placebo generally slightly favored not Hispanic or Latino participants. The majority of participants who received placebo and in Cohorts 1 and 2 were White, while the majority of participants in Cohort 3 were Black or African American.

*Evaluation of the protective role of RJX in the lipopolysaccharide-galactosamine (LPS-GalN) model of fatal cytokine storm, ARDS and multi-organ failure*

The ability of RJX to prevent fatal shock, ARDS, and multi-organ failure was examined in the well-established LPS-GalN model [1]. In this model LPS is combined with GalN which further sensitizes mice to LPS-induced systemic inflammatory syndrome and multi-organ failure. 60 male BALB/c mice (6-8 weeks old) were purchased from the Firat University Experimental Animal Center. Mice had access to standard rodent chow and water throughout the study ad libitum. The care and treatment of the animals were in accordance with the guidelines established by the Use of Laboratory Animals and the research protocol was approved by the Animal Care and Use Committee of Firat University (Project No. 04052020-391-046). BALB/c mice were randomly divided into 6 treatment groups with 10 mice in each group. Untreated normal control mice (Group 1) did not receive any treatments. Vehicle control mice (Group 2) were treated with 500 μL (= 0.5 mL) normal saline (NS), i.e., an aqueous solution of 0.9% NaCl, instead of RJX. NS was administered intraperitoneally (*i.p*) 2 hours before and 2 hours after the *i.p* injection of LPS-GalN. Test mice (Groups 3-6) received the designated RJX dose (500 μL/mouse = 0.5 mL/mouse) at the indicated dose levels (0.5, 1.05, 2.1, 4.2 mL/kg) 2 hours before and 2 hours after the *i.p* injection of LPS-GalN. For all groups of test mice, RJX doses were prepared as a 1:1:10 mixture of A+B+NS. 1:1:10 refers to a volume:volume:volume mixture of RJX used in these experiments (i.e., Vial A volume : Vial B Volume : Normal saline diluent volume = 1:1:10). Hence, 1 cc of Vial A content was mixed with 1 mL of Vial B content and then added to 10 cc of normal saline. In other words, RJX (Vial A + Vial B in equal amounts) was diluted 6-fold (2 mL in 12 mL total volume).

Groups were as follows:

1. Untreated normal control
2. LPS+ D-galactosamine+vehicle (NS)
3. LPS+ D-galactosamine+RJX 0.5 mL/kg (1:1:10 mixture of A + B + NS)
4. LPS+ D-galactosamine+RJX 1.05 ml/kg (1:1:10 mixture of A + B + NS)
5. LPS+ D-galactosamine+RJX 2.1 mL/kg (1:1:10 mixture of A + B + NS)
6. LPS+ D-galactosamine+RJX 4.2 mL/kg (1:1:10 mixture of A + B + NS)

All mice except for the untreated normal control mice were challenged with an otherwise lethal dose of LPS mixed with GalN. Specifically, all mice in Groups 2-6 were challenged with an *i.p.* injection of LPS plus D-galactosamine (Sigma, St. Louis, MO). D-Galactosamine (Sigma Chemicals), which was dissolved at a 32 mg/ml final concentration in PBS, was mixed with an equal volume of diluted, sonicated LPS immediately before dosing. This freshly prepared LPS-galactosamine mixture (LPS-GalN) was used immediately after preparation. Each mouse received a 500 µL i.p. injection of LPS-GalN (consisting of 100 ng of LPS plus 8 mg of D-galactosamine). Mice were monitored for mortality for 48 h. For mice from different treatment groups (Groups 2-6) that died within 24 hours after the i.p injection of LPS-GalN, blood samples were collected and pooled for measurement of serum inflammatory markers (IL-6, TNF-$\alpha$, and LDH). Each test was repeated six times. IL-6 and TNFα levels in mouse serum samples were measured by quantitative enzyme-linked immunosorbent assays (ELISA) using the commercially available "Mouse IL-6 Quantikine ELISA Kit" (Sensitivity for mouse IL-6: 1.6 pg/mL, Assay Range: 7.8 - 500 pg/mL) and "Mouse TNFa Quantikine ELISA Kit" (Sensivity for mouse TNF$\alpha$: 7.21 pg/mL, Assay Range: 10.9 - 700 pg/mL), respectively, and an absorbance microplate reader (Bio-Tek Elx800 Universal Microplate Reader, Bio-Tek Instruments, Inc, Winooski, USA) according to the manufacturer's instructions (R&D Systems, Minneapolis, MN, USA). Samples and standards were analyzed in duplicate. The Kaplan-Meier method, log-rank chi-square test was used to analyze the 48 h survival outcomes of mice in the different treatment groups. At the time of death, lungs, heart, kidneys, liver, and brain from 6 mice per group were harvested, fixed in 10% buffered formalin, and processed for histopathologic examination. 3 µm sections were cut, deparaffinized, dehydrated and stained with hematoxylin and eosin (H & E) and examined with light microscopy.

Lipid peroxidation was determined as thiobarbituric acid-reactive substances (TBARS) in serum and tissue samples and the values of TBARS material were expressed in terms of malondialdehyde (MDA, nmol/ml serum or nmol/g tissue) as a biomarker of oxidative stress were also measured in the liver, lungs, and brain, as previously described [2, 3]. Western blot analysis of tissue cytokine expression levels was done as described previously [4]. Briefly, tissue lysates were prepared in ice-cold RIPA buffer mixed with protease inhibitors. Protein content was measured Qubit 2.0 Fluorometer according to the manufacturer's protocol (Invitrogen, Life Technologies

Corporation, Carlsbad, CA, USA). Tissue proteins (30 μg protein/lane) were resolved by 12% Sodium dodecyl sulfate-polyacrylamide gel electrophoresis (SDS–PAGE), then transferred into a 0.45 um nitrocellulose membrane. The transferred proteins were blocked with bovine serum albumin to prevent non-specific interactions, will be incubated overnight with the diluted (1:1000) primary antibodies from Santa Cruz Bio. for IL-1β, IL-6, TNF-α, and TGF-β. The blotted membranes were washed and incubated with the secondary antibody. The mouse monoclonal β-actin antibody was used (Sigma–Aldrich) to control the protein loading. Specific binding between primary and secondary antibodies was visualized using diaminobenzidine and $H_2O_2$ as substrates. The membranes were scanned and bands were analyzed densitometrically using an image analysis system (Image J; National Institute of Health, Bethesda, USA). Concentrations of ascorbic acid in the liver and lung were measured by HPLC [5]. Safety pharmacology labs, serum and tissue cytokine levels, tissue activity levels of superoxide dismutase (SOD), catalase (CAT) and glutathione peroxidase (GSH-Px), serum and tissue ascorbic acid levels, as well as tissue concentrations of malondialdehyde (MDA), were determined using samples obtained at the time of death or termination to examine the effects of RJX on the inflammatory process caused by LPS-GalN. Serum concentrations of, aspartate aminotransferase (AST), alanine aminotransferase (ALT), urea, creatinine, LDH, ALP were assayed using an automated chemistry analyzer (Samsung LABGEO PT10, Samsung Electronics Co., Suwon, Korea). The enzymatic activities of SOD, CAT and GSH-Px in the lung and liver specimens were determined using the commercially available kits (Cayman Chemical, Ann Arbor, MI, USA) according to the manufacturer's procedures.

**References**

1. Uckun FM, Tibbles H, Ozer Z, Qazi S, Vassilev A. Anti-inflammatory activity profile of JANEX-1 in preclinical animal models. Bioorg Med Chem. 2008 Feb 1;16(3):1287-98. doi: 10.1016/j.bmc.2007.10.066.

2. Sahin K, Orhan C, Kucuk O, Sahin N, Tuzcu M, Er B, Durkee S, Bellamine A. A Dose-Dependent Effect of Carnipure® Tartrate Supplementation on Endurance Capacity, Recovery, and Body Composition in an Exercise Rat Model.Nutrients. 2020 May 23;12(5):E1519. doi: 10.3390/nu12051519.

3. Karatas A, Dagli AF, Orhan C, Gencoglu H, Ozgen M, Sahin N, Sahin K, Koca SS. Epigallocatechin 3-gallate attenuates arthritis by regulating Nrf2, HO-1, and **cytokine** levels in an experimental arthritis model. Biotechnol Appl Biochem. 2019 Nov 19. doi: 10.1002/bab.1860.

4. Sahin K, Yabas M, Orhan C, Tuzcu M, Sahin TK, Ozercan IH, Qazi S, Uckun FM. Prevention of DMBA-induced mammary gland tumors in mice by a dual-function inhibitor of JAK3 and EGF

receptor tyrosine kinases. Expert Opin Ther Targets. 2020 Apr;24(4):379-387. doi: 10.1080/14728222.2020.1737014. Epub 2020 Mar 2.
5. Barim O., Karatepe M The Effects of Pollution on the Vitamins A, E, C, Beta-Carotene Contents and Oxidative Stress f the Freshwater Crayfish, Astacus Leptodactylus. Ecotoxicol Environ Saf . 2010 Feb;73(2):138-42. doi: 10.1016/j.ecoenv.2009.08.002.

# SUPPLEMENTAL TABLES

**Table S1. Quantitative Composition of RJX**

| Vial | Component | % Content | Content mg/10 mL |
|---|---|---|---|
| Vial A | **API (designated by numbers)** | | |
| | 1) Ascorbic Acid USP | 8.9933 | 899.33 |
| | 2) Thiamine HCl USP | 0.6333 | 63.33 |
| | 3) Magnesium Sulfate Heptahydrate USP | 8.080 | 808.00 |
| | 4) Cyanocobalamin Crystalline USP | 0.0193 | 1.93 |
| | 5) Niacinamide USP | 1.188 | 118.80 |
| | 6) Pyridoxine HCl USP | 1.188 | 118.80 |
| | 7) Riboflavin 5'Phosphate USP | 0.0253 | 2.53 |
| | 8) Calcium D-Pantothenate USP | 0.0293 | 2.93 |
| | Water for Injection USP (diluent) | 79.8425 | 7984.25 |
| | Sodium Chloride USP | 0.001 | 0.1 |
| Vial B | Sodium Bicarbonate USP | 8.40 | 840.00 |
| | Water for Injection USP (diluent) | 91.599 | 9159.90 |
| | Sodium Chloride USP | 0.001 | 0.1 |

Abbreviations: USP = United States Pharmacopeia, API = Active Pharmaceutical Ingredient

**Table S2. Summary of Participant Disposition (ITT Population)**

| | Placebo (N = 13) | Part 1, Cohort 1 0.024 mL/kg (N = 6) | 2 0.076 mL/kg (N = 6) | 3 0.240 mL/kg (N = 6) | 4 0.500 mL/kg (N = 6) | 5 0.759 mL/kg (N = 6) | 6 0.500 mL/kg (N = 9) | Total (N = 52) |
|---|---|---|---|---|---|---|---|---|
| Randomized (ITT Population) | 13 | 6 | 6 | 6 | 6 | 6 | 9 | 52 |
| Dosed (Safety Analysis Population) | 13 | 6 | 6 | 6 | 6 | 6 | 9 | 52 |
| Dosed with Placebo | 13 | 0 | 0 | 0 | 0 | 0 | 0 | 13 |
| Dosed with RJX | 0 | 6 | 6 | 6 | 6 | 6 | 9 | 39 |
| Completed Study (N [%] of Participants) | 13 (100.0) | 6 (100.0) | 6 (100.0) | 6 (100.0) | 6 (100.0) | 6 (100.0) | 9 (100.0) | 52 (100.0) |
| Discontinued Early from the Study | 0 | 0 | 0 | 0 | 0 | 0 | 0 | 0 |

| | Placebo (N = 6) | Part 2, Cohort 1 0.240 mL/kg (N = 6) | 2 0.500 mL/kg (N = 6) | 3 0.759 mL/kg (N = 6) | Total (N = 24) |
|---|---|---|---|---|---|
| Randomized (ITT Population) | 6 | 6 | 6 | 6 | 24 |
| Dosed (Safety Analysis Population) | 6 | 6 | 6 | 6 | 24 |
| Dosed with Placebo | 6 | 0 | 0 | 0 | 6 |
| Dosed with RJX | 0 | 6 | 6 | 6 | 18 |
| Completed Study (N [%] of Participants) | 6 (100.0) | 6 (100.0) | 6 (100.0) | 6 (100.0) | 24 (100.0) |
| Discontinued Early from the Study | 0 | 0 | 0 | 0 | 0 |

Abbreviations: ITT = Intent to Treat; N = number; RJX = Rejuveinix.

## Table S3.Demographic and Baseline Characteristics of Subjects on Protocol No. RPI003; ClinicalTrials.gov Identifier: NCT03680105).

| | **Part 1: Single Ascending Dose (SAD)** | | | | | | | | **Part 2: Multiple Ascending Dose (MAD)** | | | | |
|---|---|---|---|---|---|---|---|---|---|---|---|---|---|
| | | **Cohort** | | | | | | | | **Cohort** | | | |
| **Parameter (units)** | **Placebo (N = 13)** | **1 0.024 mL/kg (N = 6)** | **2 0.076 mL/kg (N = 6)** | **3 0.240 mL/kg (N = 6)** | **4 0.500 mL/kg (N = 6)** | **5 0.759 mL/kg (N = 6)** | **6 0.500 mL/kg (N = 9)** | **Total (N = 52)** | **Placebo (N = 6)** | **1 0.240 mL/kg (N = 6)** | **2 0.500 mL/kg (N = 6)** | **3 0.759 mL/kg (N = 6)** | **Total (N = 24)** |
| **Age** (years), Mean (SD) | 34.8 (13.5) | 28.0 (7.0) | 32.3 (8.5) | 32.5 (9.6) | 33.7 (5.6) | 27.3 (8.8) | 54.6 (3.5) | 35.9 (12.6) | 36.2 (10.3) | 37.2 (8.1) | 37.3 (6.7) | 35.8 (12.8) | 36.6 (9.1) |
| Median | 29.0 | 27.5 | 35.5 | 34.5 | 35.0 | 24.0 | 54.0 | 34.0 | 36.0 | 38.5 | 40.0 | 34.5 | 39.0 |
| **Sex** (n [%]) Male | 6 (46.2) | 2 (33.3) | 3 (50.0) | 2 (33.3) | 2 (33.3) | 3 (50.0) | 4 (44.4) | 22 (42.3) | 4 (66.7) | 6 (100.0) | 3 (50.0) | 2 (33.3) | 15 (62.5) |
| Female | 7 (53.8) | 4 (66.7) | 3 (50.0) | 4 (66.7) | 4 (66.7) | 3 (50.0) | 5 (55.6) | 30 (57.7) | 2 (33.3) | 0 | 3 (50.0) | 4 (66.7) | 9 (37.5) |
| **Ethnicity** (n [%]) | | | | | | | | | | | | | |
| Hispanic or Latino | 5 (38.5) | 5 (83.3) | 2 (33.3) | 3 (50.0) | 2 (33.3) | 3 (50.0) | 6 (66.7) | 26 (50.0) | 2 (33.3) | 2 (33.3) | 3 (50.0) | 2 (33.3) | 9 (37.5) |
| Not Hispanic or Latino | 8 (61.5) | 1 (16.7) | 4 (66.7) | 3 (50.0) | 4 (66.7) | 3 (50.0) | 3 (33.3) | 26 (50.0) | 4 (66.7) | 4 (66.7) | 3 (50.0) | 4 (66.7) | 15 (62.5) |
| **Race** (n [%]) Black or African American | 8 (61.5) | 1 (16.7) | 1 (16.7) | 2 (33.3) | 3 (50.0) | 2 (33.3) | 2 (22.2) | 19 (36.5) | 2 (33.3) | 2 (33.3) | 0 | 5 (83.3) | 9 (37.5) |
| White | 5 (38.5) | 5 (83.3) | 5 (83.3) | 4 (66.7) | 3 (50.0) | 4 (66.7) | 7 (77.8) | 33 (63.5) | 4 (66.7) | 3 (50.0) | 6 (100.0) | 1 (16.7) | 14 (58.3) |
| Native Hawaiian or Other Pacific Islander | 0 | 0 | 0 | 0 | 0 | 0 | 0 | 0 | 0 | 1 (16.7) | 0 | 0 | 1 (4.2) |
| **Weight** (kg) Mean (SD) | 79.01 (16.95) | 66.48 (14.87) | 75.47 (11.25) | 72.48 (10.00) | 69.28 (4.67) | 72.25 (15.27) | 77.58 (13.11) | 74.25 (13.50) | 90.77 (19.17) | 83.52 (7.96) | 67.08 (7.47) | 72.60 (9.78) | 78.49 (14.68) |
| Median | 82.60 | 65.20 | 75.15 | 69.50 | 67.80 | 72.45 | 75.90 | 71.20 | 92.80 | 85.10 | 68.50 | 77.55 | 77.65 |
| **Height** (cm) Mean (SD) | 167.15 (9.30) | 161.40 (9.54) | 170.73 (7.51) | 166.33 (8.43) | 168.42 (9.45) | 167.28 (12.69) | 166.19 (10.21) | 166.80 (9.40) | 169.53 (13.25) | 175.00 (5.84) | 163.63 (10.56) | 164.70 (6.70) | 168.22 (10.04) |
| Median | 166.50 | 158.95 | 172.30 | 165.25 | 168.35 | 167.40 | 164.20 | 166.60 | 169.05 | 175.00 | 164.10 | 166.00 | 168.40 |
| **BMI** (kg/m$^2$) Mean (SD) | 27.79 (4.01) | 25.27 (3.46) | 25.87 (3.93) | 26.15 (4.27) | 24.47 (3.60) | 25.58 (4.15) | 27.81 (4.40) | 26.45 (3.98) | 31.07 (2.52) | 27.15 (2.34) | 24.88 (3.13) | 26.58 (2.42) | 27.42 (3.36) |
| Median | 28.60 | 23.90 | 27.40 | 26.20 | 24.25 | 27.15 | 27.20 | 26.25 | 31.50 | 27.10 | 24.05 | 26.70 | 27.05 |

Abbreviations: BMI = body mass index; ITT = Intent to Treat; N or n = number; Max = maximum; Min = minimum; RJX = Rejuveinix; SD = standard deviation

**Table S4. Part 1 Summary of Treatment-emergent Adverse Events by System Organ Class and Preferred Term (Safety Analysis Population)**

| | | | Cohort | | | | | | | | | | | | | |
|---|---|---|---|---|---|---|---|---|---|---|---|---|---|---|---|---|
| | Placebo (N = 13) | | 1 0.024 mL/kg (N = 6) | | 2 0.076 mL/kg (N = 6) | | 3 0.240 mL/kg (N = 6) | | 4 0.500 mL/kg (N = 6) | | 5 0.759 mL/kg (N = 6) | | 6 0.500 mL/kg (N = 9) | | Overall (N = 52) | |
| Category | No. Particip. (%) | No. Events (%) | No. Particip. (%) | No. Events (%) | No. Particip. (%) | No. Events (%) | No. Particip. (%) | No. Events (%) | No. Particip. (%) | No. Events (%) | No. Particip. (%) | No. Events (%) | No. Particip. (%) | No. Events (%) | No. Particip. (%) | No. Events (%) |
| Any TEAE | 2 (15.4) | 2 | 1 (16.7) | 2 | 2 (33.3) | 3 | 0 | 0 | 3 (50.0) | 3 | 2 (33.3) | 3 | 0 | 0 | 10 (19.2) | 13 |
| Nervous system disorders | 0 | 0 | 1 (16.7) | 1 | 1 (16.7) | 1 | 0 | 0 | 2 (33.3) | 2 | 2 (33.3) | 3 | 0 | 0 | 6 (11.5) | 7 |
| Headache | 0 | 0 | 1 (16.7) | 1 | 0 | 0 | 0 | 0 | 1 (16.7) | 1 | 2 (33.3) | 2 | 0 | 0 | 4 (7.7) | 4 |
| Dizziness | 0 | 0 | 0 | 0 | 1 (16.7) | 1 | 0 | 0 | 0 | 0 | 1 (16.7) | 1 | 0 | 0 | 2 (3.8) | 2 |
| Delayed sleep phase | 0 | 0 | 0 | 0 | 0 | 0 | 0 | 0 | 1 (16.7) | 1 | 0 | 0 | 0 | 0 | 1 (1.9) | 1 |
| Musculo-skeletal and connective tissue disorders | 1 (7.7) | 1 | 0 | 0 | 1 (16.7) | 2 | 0 | 0 | 0 | 0 | 0 | 0 | 0 | 0 | 2 (3.8) | 3 |
| Back pain | 1 (7.7) | 1 | 0 | 0 | 1 (16.7) | 1 | 0 | 0 | 0 | 0 | 0 | 0 | 0 | 0 | 2 (3.8) | 2 |
| Musculo-skeletal chest pain | 0 | 0 | 0 | 0 | 1 (16.7) | 1 | 0 | 0 | 0 | 0 | 0 | 0 | 0 | 0 | 1 (1.9) | 1 |
| Gastro-intestinal disorders | 0 | 0 | 0 | 0 | 0 | 0 | 0 | 0 | 1 (16.7) | 1 | 0 | 0 | 0 | 0 | 1 (1.9) | 1 |
| Nausea | 0 | 0 | 0 | 0 | 0 | 0 | 0 | 0 | 1 (16.7) | 1 | 0 | 0 | 0 | 0 | 1 (1.9) | 1 |
| Investigations | 0 | 0 | 1 (16.7) | 1 | 0 | 0 | 0 | 0 | 0 | 0 | 0 | 0 | 0 | 0 | 1 (1.9) | 1 |
| Blood pressure systolic decreased | 0 | 0 | 1 (16.7) | 1 | 0 | 0 | 0 | 0 | 0 | 0 | 0 | 0 | 0 | 0 | 1 (1.9) | 1 |
| Vascular disorders | 1 (7.7) | 1 | 0 | 0 | 0 | 0 | 0 | 0 | 0 | 0 | 0 | 0 | 0 | 0 | 1 (1.9) | 1 |
| Hypotension | 1 (7.7) | 1 | 0 | 0 | 0 | 0 | 0 | 0 | 0 | 0 | 0 | 0 | 0 | 0 | 1 (1.9) | 1 |

Abbreviations: No. = number; Particip. = participants; RJX = Rejuveinix; TEAE = treatment-emergent adverse event.
Note: Participants with multiple events within a system organ class/preferred term are counted only once in the system organ class/preferred term. For the number of events, participants are counted more than once.

**Table S5. Part 2 Summary of Treatment-emergent Adverse Events by System Organ Class and Preferred Term (Safety Analysis Population)**

| | | | Cohort | | | | | | | |
|---|---|---|---|---|---|---|---|---|---|---|
| | **Placebo** (N = 6) | | **1 0.240 mL/kg** (N = 6) | | **2 0.500 mL/kg** (N = 6) | | **3 0.759 mL/kg** (N = 6) | | **Total** (N = 24) | |
| **Category** | **No. Particip.** (%) | **No. Events** (%) | **No. Particip.** (%) | **No. Events** (%) | **No. Particip.** (%) | **No. Events** (%) | **No. Particip.** (%) | **No. Events** (%) | **No. Particip.** (%) | **No. Events** (%) |
| Any TEAE | 1 (16.7) | 4 | 1 (16.7) | 1 | 3 (50.0) | 5 | 3 (50.0) | 4 | 8 (33.3) | 14 |
| General disorders and administration site conditions | 0 | 0 | 0 | 0 | 3 (50.0) | 4 | 0 | 0 | 3 (12.5) | 4 |
| Infusion site discomfort | 0 | 0 | 0 | 0 | 1 (16.7) | 1 | 0 | 0 | 1 (4.2) | 1 |
| Infusion site pain | 0 | 0 | 0 | 0 | 1 (16.7) | 2 | 0 | 0 | 1 (4.2) | 2 |
| Infusion site reaction | 0 | 0 | 0 | 0 | 1 (16.7) | 1 | 0 | 0 | 1 (4.2) | 1 |
| Musculoskeletal and connective tissue disorders | 1 (16.7) | 1 | 0 | 0 | 0 | 0 | 2 (33.3) | 2 | 3 (12.5) | 3 |
| Back pain | 0 | 0 | 0 | 0 | 0 | 0 | 1 (16.7) | 1 | 1 (4.2) | 1 |
| Myalgia | 1 (16.7) | 1 | 0 | 0 | 0 | 0 | 0 | 0 | 1 (4.2) | 1 |
| Pain in extremity | 0 | 0 | 0 | 0 | 0 | 0 | 1 (16.7) | 1 | 1 (4.2) | 1 |
| Nervous system disorders | 1 (16.7) | 2 | 0 | 0 | 1 (16.7) | 1 | 1 (16.7) | 1 | 3 (12.5) | 4 |
| Headache | 1 (16.7) | 1 | 0 | 0 | 0 | 0 | 1 (16.7) | 1 | 2 (8.3) | 2 |
| Clonus | 0 | 0 | 0 | 0 | 1 (16.7) | 1 | 0 | 0 | 1 (4.2) | 1 |
| Dizziness | 1 (16.7) | 1 | 0 | 0 | 0 | 0 | 0 | 0 | 1 (4.2) | 1 |
| Blood and lymphatic system disorders | 0 | 0 | 0 | 0 | 0 | 0 | 1 (16.7) | 1 | 1 (4.2) | 1 |
| Anaemia | 0 | 0 | 0 | 0 | 0 | 0 | 1 (16.7) | 1 | 1 (4.2) | 1 |
| Infections and infestations | 0 | 0 | 1 (16.7) | 1 | 0 | 0 | 0 | 0 | 1 (4.2) | 1 |
| Upper respiratory tract infection | 0 | 0 | 1 (16.7) | 1 | 0 | 0 | 0 | 0 | 1 (4.2) | 1 |
| Investigations | 1 (16.7) | 1 | 0 | 0 | 0 | 0 | 0 | 0 | 1 (4.2) | 1 |
| C-reactive protein increased | 1 (16.7) | 1 | 0 | 0 | 0 | 0 | 0 | 0 | 1 (4.2) | 1 |

Abbreviations: No. = number; Particip. = participants; RJX = Rejuveinix; TEAE = treatment-emergent adverse event.
Note: Participants with multiple events within a system organ class/preferred term are counted only once in the system organ class/preferred term. For the number of events, participants are counted more than once.

## Table S6-A. Summary of Treatment-Emergent Adverse Events in Part 1 by Relationship to RJX (Safety Analysis Population)

| System Organ Class<br>Preferred Term | Relationship | Placebo (N=13) No. of Subjects (%) | Placebo (N=13) No. of Events | Cohort 1 0.024 mL/kg (N=6) No. of Subjects (%) | Cohort 1 0.024 mL/kg (N=6) No. of Events | Cohort 2 0.076 mL/kg (N=6) No. of Subjects (%) | Cohort 2 0.076 mL/kg (N=6) No. of Events | Cohort 3 0.240 mL/kg (N=6) No. of Subjects (%) | Cohort 3 0.240 mL/kg (N=6) No. of Events |
|---|---|---|---|---|---|---|---|---|---|
| Any TEAE | Unrelated | 0 | 0 | 0 | 0 | 0 | 0 | 0 | 0 |
| | Unlikely Related | 2 (15.4) | 2 | 1 (16.7) | 1 | 1 (16.7) | 2 | 0 | 0 |
| | Possibly Related | 0 | 0 | 1 (16.7) | 1 | 1 (16.7) | 1 | 0 | 0 |
| | Probably Related | 0 | 0 | 0 | 0 | 0 | 0 | 0 | 0 |
| | Highly Probably Related | 0 | 0 | 0 | 0 | 0 | 0 | 0 | 0 |
| Nervous system disorders | Unrelated | 0 | 0 | 0 | 0 | 0 | 0 | 0 | 0 |
| | Unlikely Related | 0 | 0 | 1 (16.7) | 1 | 0 | 0 | 0 | 0 |
| | Possibly Related | 0 | 0 | 0 | 0 | 1 (16.7) | 1 | 0 | 0 |
| | Probably Related | 0 | 0 | 0 | 0 | 0 | 0 | 0 | 0 |
| | Highly Probably Related | 0 | 0 | 0 | 0 | 0 | 0 | 0 | 0 |
| Headache | Unrelated | 0 | 0 | 0 | 0 | 0 | 0 | 0 | 0 |
| | Unlikely Related | 0 | 0 | 1 (16.7) | 1 | 0 | 0 | 0 | 0 |
| | Possibly Related | 0 | 0 | 0 | 0 | 0 | 0 | 0 | 0 |
| | Probably Related | 0 | 0 | 0 | 0 | 0 | 0 | 0 | 0 |
| | Highly Probably Related | 0 | 0 | 0 | 0 | 0 | 0 | 0 | 0 |
| Dizziness | Unrelated | 0 | 0 | 0 | 0 | 0 | 0 | 0 | 0 |
| | Unlikely Related | 0 | 0 | 0 | 0 | 0 | 0 | 0 | 0 |
| | Possibly Related | 0 | 0 | 0 | 0 | 1 (16.7) | 1 | 0 | 0 |
| | Probably Related | 0 | 0 | 0 | 0 | 0 | 0 | 0 | 0 |
| | Highly Probably Related | 0 | 0 | 0 | 0 | 0 | 0 | 0 | 0 |
| Delayed sleep phase | Unrelated | 0 | 0 | 0 | 0 | 0 | 0 | 0 | 0 |
| | Unlikely Related | 0 | 0 | 0 | 0 | 0 | 0 | 0 | 0 |
| | Possibly Related | 0 | 0 | 0 | 0 | 0 | 0 | 0 | 0 |
| | Probably Related | 0 | 0 | 0 | 0 | 0 | 0 | 0 | 0 |
| | Highly Probably Related | 0 | 0 | 0 | 0 | 0 | 0 | 0 | 0 |
| Musculoskeletal and connective tissue disorders | Unrelated | 0 | 0 | 0 | 0 | 0 | 0 | 0 | 0 |
| | Unlikely Related | 1 ( 7.7) | 1 | 0 | 0 | 1 (16.7) | 2 | 0 | 0 |
| | Possibly Related | 0 | 0 | 0 | 0 | 0 | 0 | 0 | 0 |
| | Probably Related | 0 | 0 | 0 | 0 | 0 | 0 | 0 | 0 |
| | Highly Probably Related | 0 | 0 | 0 | 0 | 0 | 0 | 0 | 0 |
| Investigations | Unrelated | 0 | 0 | 0 | 0 | 0 | 0 | 0 | 0 |
| | Unlikely Related | 0 | 0 | 0 | 0 | 0 | 0 | 0 | 0 |
| | Possibly Related | 0 | 0 | 1 (16.7) | 1 | 0 | 0 | 0 | 0 |

| | | | | | | | | | |
|---|---|---|---|---|---|---|---|---|---|
| | Probably Related | 0 | 0 | 0 | 0 | 0 | 0 | 0 | 0 |
| | Highly Probably Related | 0 | 0 | 0 | 0 | 0 | 0 | 0 | 0 |
| Syst. BP decreased | Unrelated | 0 | 0 | 0 | 0 | 0 | 0 | 0 | 0 |
| | Unlikely Related | 0 | 0 | 0 | 0 | 0 | 0 | 0 | 0 |
| | Possibly Related | 0 | 0 | 1 (16.7) | 1 | 0 | 0 | 0 | 0 |
| | Probably Related | 0 | 0 | 0 | 0 | 0 | 0 | 0 | 0 |
| | Highly Probably Related | 0 | 0 | 0 | 0 | 0 | 0 | 0 | 0 |

| | | Cohort 4 0.500 mL/kg (N=6) | | Cohort 5 0.759 mL/kg (N=6) | | Cohort 6 0.500 mL/kg (N=9) | | Overall (N=52) | |
|---|---|---|---|---|---|---|---|---|---|
| **System Organ Class Preferred Term** | **Relationship** | **No. of Subjects** (%) | **No. of Events** | **No. of Subjects** (%) | **No. of Events** | **No. of Subjects** (%) | **No. of Events** | **No. of Subjects** (%) | **No. of Events** |
| Any TEAE | Unrelated | 0 | 0 | 0 | 0 | 0 | 0 | 0 | 0 |
| | Unlikely Related | 1 (16.7) | 1 | 1 (16.7) | 1 | 0 | 0 | 6 (11.5) | 7 |
| | Possibly Related | 2 (33.3) | 2 | 2 (33.3) | 2 | 0 | 0 | 6 (11.5) | 6 |
| | Probably Related | 0 | 0 | 0 | 0 | 0 | 0 | 0 | 0 |
| | Highly Probably Related | 0 | 0 | 0 | 0 | 0 | 0 | 0 | 0 |
| Nervous system disorders | Unrelated | 0 | 0 | 0 | 0 | 0 | 0 | 0 | 0 |
| | Unlikely Related | 0 | 0 | 1 (16.7) | 1 | 0 | 0 | 2 ( 3.8) | 2 |
| | Possibly Related | 2 (33.3) | 2 | 2 (33.3) | 2 | 0 | 0 | 5 ( 9.6) | 5 |
| | Probably Related | 0 | 0 | 0 | 0 | 0 | 0 | 0 | 0 |
| | Highly Probably Related | 0 | 0 | 0 | 0 | 0 | 0 | 0 | 0 |
| Headache | Unrelated | 0 | 0 | 0 | 0 | 0 | 0 | 0 | 0 |
| | Unlikely Related | 0 | 0 | 1 (16.7) | 1 | 0 | 0 | 2 ( 3.8) | 2 |
| | Possibly Related | 1 (16.7) | 1 | 1 (16.7) | 1 | 0 | 0 | 2 ( 3.8) | 2 |
| | Probably Related | 0 | 0 | 0 | 0 | 0 | 0 | 0 | 0 |
| | Highly Probably Related | 0 | 0 | 0 | 0 | 0 | 0 | 0 | 0 |
| Dizziness | Unrelated | 0 | 0 | 0 | 0 | 0 | 0 | 0 | 0 |
| | Unlikely Related | 0 | 0 | 0 | 0 | 0 | 0 | 0 | 0 |
| | Possibly Related | 0 | 0 | 1 (16.7) | 1 | 0 | 0 | 2 ( 3.8) | 2 |
| | Probably Related | 0 | 0 | 0 | 0 | 0 | 0 | 0 | 0 |
| | Highly Probably Related | 0 | 0 | 0 | 0 | 0 | 0 | 0 | 0 |
| Delayed sleep phase | Unrelated | 0 | 0 | 0 | 0 | 0 | 0 | 0 | 0 |
| | Unlikely Related | 0 | 0 | 0 | 0 | 0 | 0 | 0 | 0 |
| | Possibly Related | 1 (16.7) | 1 | 0 | 0 | 0 | 0 | 1 ( 1.9) | 1 |
| | Probably Related | 0 | 0 | 0 | 0 | 0 | 0 | 0 | 0 |
| | Highly Probably Related | 0 | 0 | 0 | 0 | 0 | 0 | 0 | 0 |
| Musculoskeletal and connective tissue disorders | Unrelated | 0 | 0 | 0 | 0 | 0 | 0 | 0 | 0 |
| | Unlikely Related | 0 | 0 | 0 | 0 | 0 | 0 | 2 ( 3.8) | 3 |

| | | | | | | | | | |
|---|---|---|---|---|---|---|---|---|---|
| | Possibly Related | 0 | 0 | 0 | 0 | 0 | 0 | 0 | 0 |
| | Probably Related | 0 | 0 | 0 | 0 | 0 | 0 | 0 | 0 |
| | Highly Probably Related | 0 | 0 | 0 | 0 | 0 | 0 | 0 | 0 |
| Investigations | Unrelated | 0 | 0 | 0 | 0 | 0 | 0 | 0 | 0 |
| | Unlikely Related | 0 | 0 | 0 | 0 | 0 | 0 | 0 | 0 |
| | Possibly Related | 0 | 0 | 0 | 0 | 0 | 0 | 1 ( 1.9) | 1 |
| | Probably Related | 0 | 0 | 0 | 0 | 0 | 0 | 0 | 0 |
| | Highly Probably Related | 0 | 0 | 0 | 0 | 0 | 0 | 0 | 0 |
| BP, syst., decreased | Unrelated | 0 | 0 | 0 | 0 | 0 | 0 | 0 | 0 |
| | Unlikely Related | 0 | 0 | 0 | 0 | 0 | 0 | 0 | 0 |
| | Possibly Related | 0 | 0 | 0 | 0 | 0 | 0 | 1 ( 1.9) | 1 |
| | Probably Related | 0 | 0 | 0 | 0 | 0 | 0 | 0 | 0 |
| | Highly Probably Related | 0 | 0 | 0 | 0 | 0 | 0 | 0 | 0 |

## Table S6-B. Summary of Treatment-Emergent Adverse Events in Part 2 by Relationship to RJX (Safety Analysis Population)

| | | **Placebo** (N=6) | | **Cohort 1 0.240 mL/kg** (N=6) | | **Cohort 2 0.500 mL/kg** (N=6) | | **Cohort 3 0.759 mL/kg** (N=6) | | **Overall** (N=24) | |
|---|---|---|---|---|---|---|---|---|---|---|---|
| **System Organ Class Preferred Term** | **Relationship** | **No. of Subject** (%) | **No. of Events** | **No. of Subjects** (%) | **No. of Events** | **No. of Subjects** (%) | **No. of Events** | **No. of Subjects** (%) | **No. of Events** | **No. of Subject** (%) | **No. of Events** |
| Any TEAE | Unrelated | 0 | 0 | 1 (16.7) | 1 | 1 (16.7) | 1 | 1 (16.7) | 1 | 3 (12.5) | 3 |
| | Unlikely Related | 1 (16.7) | 1 | 0 | 0 | 1 (16.7) | 1 | 3 (50.0) | 3 | 5 (20.8) | 5 |
| | Possibly Related | 1 (16.7) | 3 | 0 | 0 | 1 (16.7) | 1 | 0 | 0 | 2 ( 8.3) | 4 |
| | Probably Related | 0 | 0 | 0 | 0 | 1 (16.7) | 2 | 0 | 0 | 1 ( 4.2) | 2 |
| | Highly Probably Related | 0 | 0 | 0 | 0 | 0 | 0 | 0 | 0 | 0 | 0 |
| General disorders and administration site conditions | Unrelated | 0 | 0 | 0 | 0 | 1 (16.7) | 1 | 0 | 0 | 1 ( 4.2) | 1 |
| | Unlikely Related | 0 | 0 | 0 | 0 | 0 | 0 | 0 | 0 | 0 | 0 |
| | Possibly Related | 0 | 0 | 0 | 0 | 1 (16.7) | 1 | 0 | 0 | 1 ( 4.2) | 1 |
| | Probably Related | 0 | 0 | 0 | 0 | 1 (16.7) | 2 | 0 | 0 | 1 ( 4.2) | 2 |
| | Highly Probably Related | 0 | 0 | 0 | 0 | 0 | 0 | 0 | 0 | 0 | 0 |
| Infusion site discomfort | Unrelated | 0 | 0 | 0 | 0 | 0 | 0 | 0 | 0 | 0 | 0 |
| | Unlikely Related | 0 | 0 | 0 | 0 | 0 | 0 | 0 | 0 | 0 | 0 |
| | Possibly Related | 0 | 0 | 0 | 0 | 1 (16.7) | 1 | 0 | 0 | 1 ( 4.2) | 1 |
| | Probably Related | 0 | 0 | 0 | 0 | 0 | 0 | 0 | 0 | 0 | 0 |
| | Highly Probably Related | 0 | 0 | 0 | 0 | 0 | 0 | 0 | 0 | 0 | 0 |
| Infusion site pain | Unrelated | 0 | 0 | 0 | 0 | 0 | 0 | 0 | 0 | 0 | 0 |
| | Unlikely Related | 0 | 0 | 0 | 0 | 0 | 0 | 0 | 0 | 0 | 0 |
| | Possibly Related | 0 | 0 | 0 | 0 | 0 | 0 | 0 | 0 | 0 | 0 |
| | Probably Related | 0 | 0 | 0 | 0 | 1 (16.7) | 2 | 0 | 0 | 1 ( 4.2) | 2 |

| | | | | | | | | | | | |
|---|---|---|---|---|---|---|---|---|---|---|---|
| | Highly Probably Related | 0 | 0 | 0 | 0 | 0 | 0 | 0 | 0 | 0 | 0 |
| Musculoskeletal and connective tissue disorders | Unrelated | 0 | 0 | 0 | 0 | 0 | 0 | 1 (16.7) | 1 | 1 ( 4.2) | 1 |
| | Unlikely Related | 0 | 0 | 0 | 0 | 0 | 0 | 1 (16.7) | 1 | 1 ( 4.2) | 1 |
| | Possibly Related | 1 (16.7) | 1 | 0 | 0 | 0 | 0 | 0 | 0 | 1 ( 4.2) | 1 |
| | Probably Related | 0 | 0 | 0 | 0 | 0 | 0 | 0 | 0 | 0 | 0 |
| | Highly Probably Related | 0 | 0 | 0 | 0 | 0 | 0 | 0 | 0 | 0 | 0 |
| Myalgia | Unrelated | 0 | 0 | 0 | 0 | 0 | 0 | 0 | 0 | 0 | 0 |
| | Unlikely Related | 0 | 0 | 0 | 0 | 0 | 0 | 0 | 0 | 0 | 0 |
| | Possibly Related | 1 (16.7) | 1 | 0 | 0 | 0 | 0 | 0 | 0 | 1 ( 4.2) | 1 |
| | Probably Related | 0 | 0 | 0 | 0 | 0 | 0 | 0 | 0 | 0 | 0 |
| | Highly Probably Related | 0 | 0 | 0 | 0 | 0 | 0 | 0 | 0 | 0 | 0 |
| Nervous system disorders | Unrelated | 0 | 0 | 0 | 0 | 0 | 0 | 0 | 0 | 0 | 0 |
| | Unlikely Related | 0 | 0 | 0 | 0 | 1 (16.7) | 1 | 1 (16.7) | 1 | 2 ( 8.3) | 2 |
| | Possibly Related | 1 (16.7) | 2 | 0 | 0 | 0 | 0 | 0 | 0 | 1 ( 4.2) | 2 |
| | Probably Related | 0 | 0 | 0 | 0 | 0 | 0 | 0 | 0 | 0 | 0 |
| | Highly Probably Related | 0 | 0 | 0 | 0 | 0 | 0 | 0 | 0 | 0 | 0 |
| Headache | Unrelated | 0 | 0 | 0 | 0 | 0 | 0 | 0 | 0 | 0 | 0 |
| | Unlikely Related | 0 | 0 | 0 | 0 | 0 | 0 | 1 (16.7) | 1 | 1 ( 4.2) | 1 |
| | Possibly Related | 1 (16.7) | 1 | 0 | 0 | 0 | 0 | 0 | 0 | 1 ( 4.2) | 1 |
| | Probably Related | 0 | 0 | 0 | 0 | 0 | 0 | 0 | 0 | 0 | 0 |
| | Highly Probably Related | 0 | 0 | 0 | 0 | 0 | 0 | 0 | 0 | 0 | 0 |

TEAE: Treatment-emergent adverse events; RJX: Rejuveinix. PT: Preferred term. AEs were coded using MeDRA Version 21.1. Subjects with multiple events within a system organ class/preferred term with the relationship to RJX. For the number of events, subjects were counted more than once.

**Table S7A. Summary of Treatment-Emergent Adverse Events in Part 1 by Severity Grade (Safety Analysis Population)**

| | | **Placebo** (N=13) | | **Cohort 1** 0.024 mL/kg (N=6) | | **Cohort 2** **0.076 mL/kg** (N=6) | | **Cohort 3** **0.240 mL/kg** (N=6) | |
|---|---|---|---|---|---|---|---|---|---|
| *System Organ Class Preferred Term [1]* | *Severity* | *No. of Subjects (%)* | *No. of Events* | *No. of Subjects (%)* | *No. of Events* | *No. of Subjects (%)* | *No. of Events* | *No. of Subjects (%)* | *No. of Events* |
| Any TEAE | Mild | 2 (15.4) | 2 | 1 (16.7) | 2 | 2 (33.3) | 3 | 0 | 0 |
| | Moderate | 0 | 0 | 0 | 0 | 0 | 0 | 0 | 0 |
| | Severe | 0 | 0 | 0 | 0 | 0 | 0 | 0 | 0 |
| | Life Threatening | 0 | 0 | 0 | 0 | 0 | 0 | 0 | 0 |
| Nervous system disorders | Mild | 0 | 0 | 1 (16.7) | 1 | 1 (16.7) | 1 | 0 | 0 |
| | Moderate | 0 | 0 | 0 | 0 | 0 | 0 | 0 | 0 |
| | Severe | 0 | 0 | 0 | 0 | 0 | 0 | 0 | 0 |
| | Life Threatening | 0 | 0 | 0 | 0 | 0 | 0 | 0 | 0 |
| Headache | Mild | 0 | 0 | 1 (16.7) | 1 | 0 | 0 | 0 | 0 |
| | Moderate | 0 | 0 | 0 | 0 | 0 | 0 | 0 | 0 |
| | Severe | 0 | 0 | 0 | 0 | 0 | 0 | 0 | 0 |
| | Life Threatening | 0 | 0 | 0 | 0 | 0 | 0 | 0 | 0 |
| Dizziness | Mild | 0 | 0 | 0 | 0 | 1 (16.7) | 1 | 0 | 0 |
| | Moderate | 0 | 0 | 0 | 0 | 0 | 0 | 0 | 0 |
| | Severe | 0 | 0 | 0 | 0 | 0 | 0 | 0 | 0 |
| | Life Threatening | 0 | 0 | 0 | 0 | 0 | 0 | 0 | 0 |
| Delayed sleep phase | Mild | 0 | 0 | 0 | 0 | 0 | 0 | 0 | 0 |
| | Moderate | 0 | 0 | 0 | 0 | 0 | 0 | 0 | 0 |
| | Severe | 0 | 0 | 0 | 0 | 0 | 0 | 0 | 0 |
| | Life Threatening | 0 | 0 | 0 | 0 | 0 | 0 | 0 | 0 |
| Musculoskeletal and connective tissue disorders | Mild | 1 ( 7.7) | 1 | 0 | 0 | 1 (16.7) | 2 | 0 | 0 |
| | Moderate | 0 | 0 | 0 | 0 | 0 | 0 | 0 | 0 |
| | Severe | 0 | 0 | 0 | 0 | 0 | 0 | 0 | 0 |
| | Life Threatening | 0 | 0 | 0 | 0 | 0 | 0 | 0 | 0 |
| Back pain | Mild | 1 ( 7.7) | 1 | 0 | 0 | 1 (16.7) | 1 | 0 | 0 |
| | Moderate | 0 | 0 | 0 | 0 | 0 | 0 | 0 | 0 |
| | Severe | 0 | 0 | 0 | 0 | 0 | 0 | 0 | 0 |
| | Life Threatening | 0 | 0 | 0 | 0 | 0 | 0 | 0 | 0 |
| Chest pain | Mild | 0 | 0 | 0 | 0 | 1 (16.7) | 1 | 0 | 0 |
| | Moderate | 0 | 0 | 0 | 0 | 0 | 0 | 0 | 0 |
| | Severe | 0 | 0 | 0 | 0 | 0 | 0 | 0 | 0 |
| | Life Threatening | 0 | 0 | 0 | 0 | 0 | 0 | 0 | 0 |
| Investigations | Mild | 0 | 0 | 1 (16.7) | 1 | 0 | 0 | 0 | 0 |
| | Moderate | 0 | 0 | 0 | 0 | 0 | 0 | 0 | 0 |
| | Severe | 0 | 0 | 0 | 0 | 0 | 0 | 0 | 0 |
| | Life Threatening | 0 | 0 | 0 | 0 | 0 | 0 | 0 | 0 |
| BP, syst., decreased | Mild | 0 | 0 | 1 (16.7) | 1 | 0 | 0 | 0 | 0 |
| | Moderate | 0 | 0 | 0 | 0 | 0 | 0 | 0 | 0 |
| | Severe | 0 | 0 | 0 | 0 | 0 | 0 | 0 | 0 |
| | Life Threatening | 0 | 0 | 0 | 0 | 0 | 0 | 0 | 0 |
| Vascular disorders | Mild | 1 ( 7.7) | 1 | 0 | 0 | 0 | 0 | 0 | 0 |

| | | | | | | | | | |
|---|---|---|---|---|---|---|---|---|---|
| | Moderate | 0 | 0 | 0 | 0 | 0 | 0 | 0 | 0 |
| | Severe | 0 | 0 | 0 | 0 | 0 | 0 | 0 | 0 |
| | Life Threatening | 0 | 0 | 0 | 0 | 0 | 0 | 0 | 0 |
| Hypotension | Mild | 1 ( 7.7) | 1 | 0 | 0 | 0 | 0 | 0 | 0 |
| | Moderate | 0 | 0 | 0 | 0 | 0 | 0 | 0 | 0 |
| | Severe | 0 | 0 | 0 | 0 | 0 | 0 | 0 | 0 |
| | Life Threatening | 0 | 0 | 0 | 0 | 0 | 0 | 0 | 0 |
| Gastrointestinal disorders | Mild | 0 | 0 | 0 | 0 | 0 | 0 | 0 | 0 |
| | Moderate | 0 | 0 | 0 | 0 | 0 | 0 | 0 | 0 |
| | Severe | 0 | 0 | 0 | 0 | 0 | 0 | 0 | 0 |
| | Life Threatening | 0 | 0 | 0 | 0 | 0 | 0 | 0 | 0 |
| Nausea | Mild | 0 | 0 | 0 | 0 | 0 | 0 | 0 | 0 |
| | Moderate | 0 | 0 | 0 | 0 | 0 | 0 | 0 | 0 |
| | Severe | 0 | 0 | 0 | 0 | 0 | 0 | 0 | 0 |
| | Life Threatening | 0 | 0 | 0 | 0 | 0 | 0 | 0 | 0 |

| | | **Cohort 4** 0.500 mL/kg (N=6) | | **Cohort 5** 0.759 mL/kg (N=6) | | **Cohort 6** **0.500 mL/kg** (N=9) | | **Overall** (N=52) | |
|---|---|---|---|---|---|---|---|---|---|
| *System Organ Class Preferred Term [1]* | *Severity* | *No. of Subjects (%)* | *No. of Events* | *No. of Subjects (%)* | *No. of Events* | *No. of Subjects (%)* | *No. of Events* | *No. of Subjects (%)* | *No. of Events* |
| Any TEAE | Mild | 2 (33.3) | 2 | 1 (16.7) | 2 | 0 | 0 | 8 (15.4) | 11 |
| | Moderate | 1 (16.7) | 1 | 1 (16.7) | 1 | 0 | 0 | 2 ( 3.8) | 2 |
| | Severe | 0 | 0 | 0 | 0 | 0 | 0 | 0 | 0 |
| | Life Threatening | 0 | 0 | 0 | 0 | 0 | 0 | 0 | 0 |
| Nervous system disorders | Mild | 1 (16.7) | 1 | 1 (16.7) | 2 | 0 | 0 | 4 ( 7.7) | 5 |
| | Moderate | 1 (16.7) | 1 | 1 (16.7) | 1 | 0 | 0 | 2 ( 3.8) | 2 |
| | Severe | 0 | 0 | 0 | 0 | 0 | 0 | 0 | 0 |
| | Life Threatening | 0 | 0 | 0 | 0 | 0 | 0 | 0 | 0 |
| Headache | Mild | 0 | 0 | 1 (16.7) | 1 | 0 | 0 | 2 ( 3.8) | 2 |
| | Moderate | 1 (16.7) | 1 | 1 (16.7) | 1 | 0 | 0 | 2 ( 3.8) | 2 |
| | Severe | 0 | 0 | 0 | 0 | 0 | 0 | 0 | 0 |
| | Life Threatening | 0 | 0 | 0 | 0 | 0 | 0 | 0 | 0 |
| Dizziness | Mild | 0 | 0 | 1 (16.7) | 1 | 0 | 0 | 2 ( 3.8) | 2 |
| | Moderate | 0 | 0 | 0 | 0 | 0 | 0 | 0 | 0 |
| | Severe | 0 | 0 | 0 | 0 | 0 | 0 | 0 | 0 |
| | Life Threatening | 0 | 0 | 0 | 0 | 0 | 0 | 0 | 0 |
| Delayed sleep phase | Mild | 1 (16.7) | 1 | 0 | 0 | 0 | 0 | 1 ( 1.9) | 1 |
| | Moderate | 0 | 0 | 0 | 0 | 0 | 0 | 0 | 0 |
| | Severe | 0 | 0 | 0 | 0 | 0 | 0 | 0 | 0 |
| | Life Threatening | 0 | 0 | 0 | 0 | 0 | 0 | 0 | 0 |
| Musculoskeletal and connective tissue disorders | Mild | 0 | 0 | 0 | 0 | 0 | 0 | 2 ( 3.8) | 3 |
| | Moderate | 0 | 0 | 0 | 0 | 0 | 0 | 0 | 0 |
| | Severe | 0 | 0 | 0 | 0 | 0 | 0 | 0 | 0 |
| | Life Threatening | 0 | 0 | 0 | 0 | 0 | 0 | 0 | 0 |
| Back pain | Mild | 0 | 0 | 0 | 0 | 0 | 0 | 2 ( 3.8) | 2 |
| | Moderate | 0 | 0 | 0 | 0 | 0 | 0 | 0 | 0 |
| | Severe | 0 | 0 | 0 | 0 | 0 | 0 | 0 | 0 |

| | | | | | | | | | |
|---|---|---|---|---|---|---|---|---|---|
| | Life Threatening | 0 | 0 | 0 | 0 | 0 | 0 | 0 | 0 |
| Chest pain | Mild | 0 | 0 | 0 | 0 | 0 | 0 | 1 ( 1.9) | 1 |
| | Moderate | 0 | 0 | 0 | 0 | 0 | 0 | 0 | 0 |
| | Severe | 0 | 0 | 0 | 0 | 0 | 0 | 0 | 0 |
| | Life Threatening | 0 | 0 | 0 | 0 | 0 | 0 | 0 | 0 |
| Investigations | Mild | 0 | 0 | 0 | 0 | 0 | 0 | 1 ( 1.9) | 1 |
| | Moderate | 0 | 0 | 0 | 0 | 0 | 0 | 0 | 0 |
| | Severe | 0 | 0 | 0 | 0 | 0 | 0 | 0 | 0 |
| | Life Threatening | 0 | 0 | 0 | 0 | 0 | 0 | 0 | 0 |
| BP, syst., decreased | Mild | 0 | 0 | 0 | 0 | 0 | 0 | 1 ( 1.9) | 1 |
| | Moderate | 0 | 0 | 0 | 0 | 0 | 0 | 0 | 0 |
| | Severe | 0 | 0 | 0 | 0 | 0 | 0 | 0 | 0 |
| | Life Threatening | 0 | 0 | 0 | 0 | 0 | 0 | 0 | 0 |
| Vascular disorders | Mild | 0 | 0 | 0 | 0 | 0 | 0 | 1 ( 1.9) | 1 |
| | Moderate | 0 | 0 | 0 | 0 | 0 | 0 | 0 | 0 |
| | Severe | 0 | 0 | 0 | 0 | 0 | 0 | 0 | 0 |
| | Life Threatening | 0 | 0 | 0 | 0 | 0 | 0 | 0 | 0 |
| Hypotension | Mild | 0 | 0 | 0 | 0 | 0 | 0 | 1 ( 1.9) | 1 |
| | Moderate | 0 | 0 | 0 | 0 | 0 | 0 | 0 | 0 |
| | Severe | 0 | 0 | 0 | 0 | 0 | 0 | 0 | 0 |
| | Life Threatening | 0 | 0 | 0 | 0 | 0 | 0 | 0 | 0 |
| Gastrointestinal disorders | Mild | 1 (16.7) | 1 | 0 | 0 | 0 | 0 | 1 ( 1.9) | 1 |
| | Moderate | 0 | 0 | 0 | 0 | 0 | 0 | 0 | 0 |
| | Severe | 0 | 0 | 0 | 0 | 0 | 0 | 0 | 0 |
| | Life Threatening | 0 | 0 | 0 | 0 | 0 | 0 | 0 | 0 |
| Nausea | Mild | 1 (16.7) | 1 | 0 | 0 | 0 | 0 | 1 ( 1.9) | 1 |
| | Moderate | 0 | 0 | 0 | 0 | 0 | 0 | 0 | 0 |
| | Severe | 0 | 0 | 0 | 0 | 0 | 0 | 0 | 0 |
| | Life Threatening | 0 | 0 | 0 | 0 | 0 | 0 | 0 | 0 |

### Table S7B. Summary of Treatment-Emergent Adverse Events in Part 2 by Severity Grade (Safety Analysis Population)

| | | **Placebo** (N=6) | | **Cohort 1 0.240 mL/kg** (N=6) | | **Cohort 2 0.500 mL/kg** (N=6) | | **Cohort 3 0.759 mL/kg** (N=6) | | **Overall** (N=24) | |
|---|---|---|---|---|---|---|---|---|---|---|---|
| *System Organ Class Preferred Term [1]* | *Severity* | *No. of Subjects (%)* | *No. of Events* | *No. of Subjects (%)* | *No. of Events* | *No. of Subjects (%)* | *No. of Events* | *No. of Subjects (%)* | *No. of Events* | *No. of Subjects (%)* | *No. of Events* |
| Any TEAE | Mild | 0 | 2 | 1 (16.7) | 1 | 3 (50.0) | 5 | 2 (33.3) | 3 | 6 (25.0) | 11 |
| | Moderate | 1 (16.7) | 2 | 0 | 0 | 0 | 0 | 1 (16.7) | 1 | 2 ( 8.3) | 3 |
| | Severe | 0 | 0 | 0 | 0 | 0 | 0 | 0 | 0 | 0 | 0 |
| | Life Threatening | 0 | 0 | 0 | 0 | 0 | 0 | 0 | 0 | 0 | 0 |
| General disordersand administration site conditions | Mild | 0 | 0 | 0 | 0 | 3 (50.0) | 4 | 0 | 0 | 3 (12.5) | 4 |
| | Moderate | 0 | 0 | 0 | 0 | 0 | 0 | 0 | 0 | 0 | 0 |
| | Severe | 0 | 0 | 0 | 0 | 0 | 0 | 0 | 0 | 0 | 0 |
| | Life Threatening | 0 | 0 | 0 | 0 | 0 | 0 | 0 | 0 | 0 | 0 |
| Infusion site discomfort | Mild | 0 | 0 | 0 | 0 | 1 (16.7) | 1 | 0 | 0 | 1 ( 4.2) | 1 |
| | Moderate | 0 | 0 | 0 | 0 | 0 | 0 | 0 | 0 | 0 | 0 |

| | | | | | | | | | | | |
|---|---|---|---|---|---|---|---|---|---|---|---|
| | Severe | 0 | 0 | 0 | 0 | 0 | 0 | 0 | 0 | 0 | 0 |
| | Life Threatening | 0 | 0 | 0 | 0 | 0 | 0 | 0 | 0 | 0 | 0 |
| Infusion site pain | Mild | 0 | 0 | 0 | 0 | 1 (16.7) | 2 | 0 | 0 | 1 ( 4.2) | 2 |
| | Moderate | 0 | 0 | 0 | 0 | 0 | 0 | 0 | 0 | 0 | 0 |
| | Severe | 0 | 0 | 0 | 0 | 0 | 0 | 0 | 0 | 0 | 0 |
| | Life Threatening | 0 | 0 | 0 | 0 | 0 | 0 | 0 | 0 | 0 | 0 |
| Infusion site reaction | Mild | 0 | 0 | 0 | 0 | 1 (16.7) | 1 | 0 | 0 | 1 ( 4.2) | 1 |
| | Moderate | 0 | 0 | 0 | 0 | 0 | 0 | 0 | 0 | 0 | 0 |
| | Severe | 0 | 0 | 0 | 0 | 0 | 0 | 0 | 0 | 0 | 0 |
| | Life Threatening | 0 | 0 | 0 | 0 | 0 | 0 | 0 | 0 | 0 | 0 |
| Musculoskeletal and connective tissue disorders | Mild | 0 | 0 | 0 | 0 | 0 | 0 | 1 (16.7) | 1 | 1 ( 4.2) | 1 |
| | Moderate | 1 (16.7) | 1 | 0 | 0 | 0 | 0 | 1 (16.7) | 1 | 2 ( 8.3) | 2 |
| | Severe | 0 | 0 | 0 | 0 | 0 | 0 | 0 | 0 | 0 | 0 |
| | Life Threatening | 0 | 0 | 0 | 0 | 0 | 0 | 0 | 0 | 0 | 0 |
| Back pain | Mild | 0 | 0 | 0 | 0 | 0 | 0 | 0 | 0 | 0 | 0 |
| | Moderate | 0 | 0 | 0 | 0 | 0 | 0 | 1 (16.7) | 1 | 1 ( 4.2) | 1 |
| | Severe | 0 | 0 | 0 | 0 | 0 | 0 | 0 | 0 | 0 | 0 |
| | Life Threatening | 0 | 0 | 0 | 0 | 0 | 0 | 0 | 0 | 0 | 0 |
| Myalgia | Mild | 0 | 0 | 0 | 0 | 0 | 0 | 0 | 0 | 0 | 0 |
| | Moderate | 1 (16.7) | 1 | 0 | 0 | 0 | 0 | 0 | 0 | 1 ( 4.2) | 1 |
| | Severe | 0 | 0 | 0 | 0 | 0 | 0 | 0 | 0 | 0 | 0 |
| | Life Threatening | 0 | 0 | 0 | 0 | 0 | 0 | 0 | 0 | 0 | 0 |
| Pain in extremity | Mild | 0 | 0 | 0 | 0 | 0 | 0 | 1 (16.7) | 1 | 1 ( 4.2) | 1 |
| | Moderate | 0 | 0 | 0 | 0 | 0 | 0 | 0 | 0 | 0 | 0 |
| | Severe | 0 | 0 | 0 | 0 | 0 | 0 | 0 | 0 | 0 | 0 |
| | Life Threatening | 0 | 0 | 0 | 0 | 0 | 0 | 0 | 0 | 0 | 0 |
| Nervous system disorders | Mild | 0 | 1 | 0 | 0 | 1 (16.7) | 1 | 1 (16.7) | 1 | 2 ( 8.3) | 3 |
| | Moderate | 1 (16.7) | 1 | 0 | 0 | 0 | 0 | 0 | 0 | 1 ( 4.2) | 1 |
| | Severe | 0 | 0 | 0 | 0 | 0 | 0 | 0 | 0 | 0 | 0 |
| | Life Threatening | 0 | 0 | 0 | 0 | 0 | 0 | 0 | 0 | 0 | 0 |
| Headache | Mild | 0 | 0 | 0 | 0 | 0 | 0 | 1 (16.7) | 1 | 1 ( 4.2) | 1 |
| | Moderate | 1 (16.7) | 1 | 0 | 0 | 0 | 0 | 0 | 0 | 1 ( 4.2) | 1 |
| | Severe | 0 | 0 | 0 | 0 | 0 | 0 | 0 | 0 | 0 | 0 |
| | Life Threatening | 0 | 0 | 0 | 0 | 0 | 0 | 0 | 0 | 0 | 0 |
| Clonus | Mild | 0 | 0 | 0 | 0 | 1 (16.7) | 1 | 0 | 0 | 1 ( 4.2) | 1 |
| | Moderate | 0 | 0 | 0 | 0 | 0 | 0 | 0 | 0 | 0 | 0 |
| | Severe | 0 | 0 | 0 | 0 | 0 | 0 | 0 | 0 | 0 | 0 |
| | Life Threatening | 0 | 0 | 0 | 0 | 0 | 0 | 0 | 0 | 0 | 0 |
| Dizziness | Mild | 1 (16.7) | 1 | 0 | 0 | 0 | 0 | 0 | 0 | 1 ( 4.2) | 1 |
| | Moderate | 0 | 0 | 0 | 0 | 0 | 0 | 0 | 0 | 0 | 0 |
| | Severe | 0 | 0 | 0 | 0 | 0 | 0 | 0 | 0 | 0 | 0 |
| | Life Threatening | 0 | 0 | 0 | 0 | 0 | 0 | 0 | 0 | 0 | 0 |
| Blood and lymphatic system disorders | Mild | 0 | 0 | 0 | 0 | 0 | 0 | 1 (16.7) | 1 | 1 ( 4.2) | 1 |
| | Moderate | 0 | 0 | 0 | 0 | 0 | 0 | 0 | 0 | 0 | 0 |
| | Severe | 0 | 0 | 0 | 0 | 0 | 0 | 0 | 0 | 0 | 0 |
| | Life Threatening | 0 | 0 | 0 | 0 | 0 | 0 | 0 | 0 | 0 | 0 |
| Anemia | Mild | 0 | 0 | 0 | 0 | 0 | 0 | 1 (16.7) | 1 | 1 ( 4.2) | 1 |
| | Moderate | 0 | 0 | 0 | 0 | 0 | 0 | 0 | 0 | 0 | 0 |
| | Severe | 0 | 0 | 0 | 0 | 0 | 0 | 0 | 0 | 0 | 0 |

| | | | | | | | | | | | |
|---|---|---|---|---|---|---|---|---|---|---|---|
| | Life Threatening | 0 | 0 | 0 | 0 | 0 | 0 | 0 | 0 | 0 | 0 |
| Infections and infestations | Mild | 0 | 0 | 1 (16.7) | 1 | 0 | 0 | 0 | 0 | 1 ( 4.2) | 1 |
| | Moderate | 0 | 0 | 0 | 0 | 0 | 0 | 0 | 0 | 0 | 0 |
| | Severe | 0 | 0 | 0 | 0 | 0 | 0 | 0 | 0 | 0 | 0 |
| | Life Threatening | 0 | 0 | 0 | 0 | 0 | 0 | 0 | 0 | 0 | 0 |
| URI | Mild | 0 | 0 | 1 (16.7) | 1 | 0 | 0 | 0 | 0 | 1 ( 4.2) | 1 |
| | Moderate | 0 | 0 | 0 | 0 | 0 | 0 | 0 | 0 | 0 | 0 |
| | Severe | 0 | 0 | 0 | 0 | 0 | 0 | 0 | 0 | 0 | 0 |
| | Life Threatening | 0 | 0 | 0 | 0 | 0 | 0 | 0 | 0 | 0 | 0 |

**Table S8 Summary Statistics of Plasma Pharmacokinetic Parameters of Baseline-Adjusted Ascorbic Acid After Single Ascending Dose of IV Infusion of RJX in Healthy Adult and Elderly Volunteers**

| | | Day 1 (Single Ascending Dose of Rejuveinix) | | | | | |
|---|---|---|---|---|---|---|---|
| | | Cohort 1 | Cohort 2 | Cohort 3 | Cohort 4 | Cohort 5 | Cohort 6 |
| Parameter | | RJX 0.024 mL/kg (1.079 mg/kg of Ascorbic Acid) in Healthy Subjects (n=6)[a] | RJX 0.076 mL/kg (3.417 mg/kg of Ascorbic Acid) in Healthy Subjects (n=6)[a] | RJX 0.240 mL/kg (10.792 mg/kg of Ascorbic Acid) in Healthy Subjects (n=6)[b] | RJX 0.500 mL/kg (22.483 mg/kg of Ascorbic Acid) in Healthy Subjects (n=6)[b] | RJX 0.759 mL/kg (34.130 mg/kg of Ascorbic Acid) in Healthy Subjects (n=6)[c] | RJX 0.500 mL/kg (22.483 mg/kg of Ascorbic Acid) in Elderly (n=9)[d] |
| $C_{max}$ | Mean (SD) | 5.40 (2.02) | 17.45 (2.74) | 47.97 (8.56) | 108.63 (14.82) | 152.69 (30.17) | 132.95 (22.10) |
| (µg/mL) | CV (%) | 37.3 | 15.7 | 17.8 | 13.6 | 19.8 | 16.6 |
| $AUC_{0\text{-last}}$ | Mean (SD) | 32.36 (7.30) | 80.44 (45.90) | 190.01 (50.82) | 322.68 (33.45) | 467.10 (51.07) | 406.53 (97.07) |
| (h*µg/mL) | CV (%) | 22.6 | 57.1 | 26.7 | 10.4 | 10.9 | 23.9 |
| $t_{1/2}$ | Mean (SD) | 3.64 (NC) | 2.65 (NC) | NC (NC) | NC (NC) | 16.43 (NA) | 21.27 (10.65) |
| (h) | CV (%) | NC | NC | NC | NC | NA | 50.1 |
| Cl | Mean (SD) | 0.04 (NC) | 0.12 (NC) | NC (NC) | NC (NC) | 0.06 (NA) | 0.05 (0.01) |
| ((L/h)/kg) | CV (%) | NC | NC | NC | NC | NA | 19.2 |
| V | Mean (SD) | 0.19 (NC) | 0.45 (NC) | NC (NC) | NC (NC) | 1.38 (NA) | 1.42 (0.69) |
| (L/kg) | CV (%) | NC | NC | NC | NC | NA | 48.3 |
| $t_{max}$ | Min | 0.52 | 0.75 | 0.75 | 0.75 | 0.75 | 0.75 |
| (h) | Max | 1.25 | 0.75 | 0.80 | 0.80 | 1.10 | 0.90 |

NA : Not Applicable, NC : Not Calculated

[a] n=1 for $t_{1/2}$; [b] n=0 for $t_{1/2}$; [c] n=2 for $t_{1/2}$; [d] n=4 for $t_{1/2}$

**Table S9 Summary Statistics of Plasma Pharmacokinetic Parameters of Baseline-Adjusted Cyanocobalamin After Single Ascending Dose of IV Infusion of RJX in Healthy Adult and Elderly Volunteers**

| | | Day 1 (Single Ascending Dose of RJX) | | | | | |
|---|---|---|---|---|---|---|---|
| | | Cohort 1 | Cohort 2 | Cohort 3 | Cohort 4 | Cohort 5 | Cohort 6 |
| Parameter | | RJX 0.024 mL/kg (0.002 mg/kg of Cyanocobalamin) in Healthy Subjects (n=6)[a] | RJX 0.076 mL/kg (0.007 mg/kg of Cyanocobalamin) in Healthy Subjects (n=6)[a] | RJX 0.240 mL/kg (0.023 mg/kg of Cyanocobalamin) in Healthy Subjects (n=6) | RJX 0.500 mL/kg (0.048 mg/kg of Cyanocobalamin) in Healthy Subjects (n=6) | RJX 0.759 mL/kg (0.073 mg/kg of Cyanocobalamin) in Healthy Subjects (n=6) | RJX 0.500 mL/kg (0.048 mg/kg of Cyanocobalamin) in Elderly (n=9) |
| $C_{max}$ | Mean (SD) | 11.414 (1.151) | 42.549 (8.363) | 109.124 (20.470) | 242.030 (25.013) | 368.629 (75.827) | 326.587 (44.513) |
| (ng/mL) | CV (%) | 10.1 | 19.7 | 18.8 | 10.3 | 20.6 | 13.6 |
| $AUC_{0\text{-}last}$ | Mean (SD) | 42.636 (12.638) | 119.652 (20.671) | 296.432 (41.684) | 685.949 (68.733) | 959.464 (89.802) | 899.686 (174.992) |
| (h*ng/mL) | CV (%) | 29.6 | 17.3 | 14.1 | 10.0 | 9.4 | 19.5 |
| $t_{1/2}$ | Mean (SD) | 28.24 (11.22) | 24.35 (13.03) | 7.24 (3.31) | 5.25 (0.55) | 4.84 (0.84) | 5.14 (0.53) |
| (h) | CV (%) | 39.7 | 53.5 | 45.7 | 10.5 | 17.2 | 10.2 |
| Cl | Mean (SD) | 0.035 (0.015) | 0.044 (0.013) | 0.076 (0.009) | 0.070 (0.007) | 0.076 (0.007) | 0.055 (0.010) |
| ((L/h)/kg) | CV (%) | 44.0 | 28.6 | 12.2 | 10.5 | 9.0 | 18.6 |
| V | Mean (SD) | 1.252 (0.363) | 1.381 (0.484) | 0.791 (0.374) | 0.526 (0.044) | 0.528 (0.074) | 0.408 (0.099) |
| (L/kg) | CV (%) | 29.0 | 35.0 | 47.3 | 8.4 | 14.1 | 24.2 |
| $t_{max}$ | Min | 0.75 | 0.75 | 0.75 | 0.75 | 0.75 | 0.75 |
| (h) | Max | 0.77 | 0.75 | 0.80 | 0.80 | 1.10 | 0.90 |

[a] n=4 for $t_{1/2}$

**Table S10 Summary Statistics of Serum Pharmacokinetic Parameters of Baseline-Adjusted Magnesium After Single Ascending Dose of IV Infusion of RJX in Healthy Adult and Elderly Volunteers**

| | | Day 1 (Single Ascending Dose of RJX) | | | | | |
|---|---|---|---|---|---|---|---|
| | | **Cohort 1** | **Cohort 2** | **Cohort 3** | **Cohort 4** | **Cohort 5** | **Cohort 6** |
| **Parameter** | | **RJX 0.024 mL/kg (0.096 mg/kg of Magnesium) in Healthy Subjects (n=5)[a]** | **RJX 0.076 mL/kg (0.303 mg/kg of Magnesium) in Healthy Subjects (n=6)[b]** | **RJX 0.240 mL/kg (0.956 mg/kg of Magnesium) in Healthy Subjects (n=6)[a]** | **RJX 0.500 mL/kg (1.992 mg/kg of Magnesium) in Healthy Subjects (n=6)[a]** | **RJX 0.759 mL/kg (3.024 mg/kg of Magnesium) in Healthy Subjects (n=6)[c]** | **RJX 0.500 mL/kg (1.992 mg/kg of Magnesium) in Elderly (n=9)[a]** |
| $C_{max}$ | Mean (SD) | 0.171 (0.053) | 0.283 (0.044) | 0.443 (0.103) | 0.797 (0.241) | 1.316 (0.310) | 0.938 (0.207) |
| (mg/dL) | CV (%) | 30.9 | 15.6 | 23.2 | 30.2 | 23.6 | 22.1 |
| $AUC_{0-last}$ | Mean (SD) | 2.393 (1.142) | 3.688 (1.231) | 4.998 (2.313) | 4.694 (3.399) | 9.378 (3.589) | 7.541 (2.406) |
| (h*mg/dL) | CV (%) | 47.7 | 33.4 | 46.3 | 72.4 | 38.3 | 31.9 |
| $t_{1/2}$ | Mean (SD) | 8.86 (NC) | NC (NC) | 13.24 (NC) | 19.96 (NC) | 9.46 (5.78) | 4.50 (NC) |
| (h) | CV (%) | NC | NC | NC | NC | 61.1 | NC |
| Cl | Mean (SD) | 0.002 (NC) | NC (NC) | 0.008 (NC) | 0.028 (NC) | 0.034 (0.018) | 0.028 (NC) |
| ((L/h)/kg) | CV (%) | NC | NC | NC | NC | 52.2 | NC |
| V | Mean (SD) | 0.027 (NC) | NC (NC) | 0.150 (NC) | 0.820 (NC) | 0.361 (0.041) | 0.181 (NC) |
| (L/kg) | CV (%) | NC | NC | NC | NC | 11.4 | NC |
| $t_{max}$ | Min | 8.75 | 1.25 | 0.75 | 0.75 | 0.75 | 0.75 |
| (h) | Max | 12.75 | 12.75 | 4.73 | 1.82 | 1.27 | 0.90 |

NC : Not Calculated

[a] n=1 for $t_{1/2}$; [b] n=0 for $t_{1/2}$; [c] n=4 for $t_{1/2}$

**Table S11 Summary Statistics of Plasma Pharmacokinetic Parameters of Baseline-Adjusted Niacinamide After Single Ascending Dose of IV Infusion of RJX in Healthy Adult and Elderly Volunteers**

| | | Day 1 (Single Ascending Dose of RJX) | | | | | |
|---|---|---|---|---|---|---|---|
| | | **Cohort 1** | **Cohort 2** | **Cohort 3** | **Cohort 4** | **Cohort 5** | **Cohort 6** |
| **Parameter** | | **RJX 0.024 mL/kg (0.143 mg/kg of Niacinamide) in Healthy Subjects (n=6)[a]** | **RJX 0.076 mL/kg (0.451 mg/kg of Niacinamide) in Healthy Subjects (n=6)[b]** | **RJX 0.240 mL/kg (1.426 mg/kg of Niacinamide) in Healthy Subjects (n=6)[c]** | **RJX 0.500 mL/kg (2.970 mg/kg of Niacinamide) in Healthy Subjects (n=6)[d]** | **RJX 0.759 mL/kg (4.508 mg/kg of Niacinamide) in Healthy Subjects (n=6)[e]** | **RJX 0.500 mL/kg (2.970 mg/kg of Niacinamide) in Elderly (n=9)** |
| $C_{max}$ | Mean (SD) | 90.1 (12.8) | 495.6 (142.3) | 1290.8 (313.8) | 3472.2 (1038.5) | 7072.5 (2475.3) | 5862.9 (1529.2) |
| (ng/mL) | CV (%) | 14.2 | 28.7 | 24.3 | 29.9 | 35.0 | 26.1 |
| $AUC_{0\text{-}last}$ | Mean (SD) | 111.5 (72.1) | 542.2 (218.7) | 1507.8 (578.5) | 4770.6 (2159.0) | 14087.7 (6257.0) | 7910.5 (3108.3) |
| (h*ng/mL) | CV (%) | 64.7 | 40.3 | 38.4 | 45.3 | 44.4 | 39.3 |
| $t_{1/2}$ | Mean (SD) | NC (NC) | 0.50 (NA) | 0.41 (0.13) | 0.59 (0.38) | 1.51 (1.49) | 0.59 (0.26) |
| (h) | CV (%) | NC | NA | 32.9 | 63.8 | 99.1 | 43.3 |
| Cl | Mean (SD) | NC (NC) | 0.6 (NA) | 0.9 (0.3) | 0.7 (0.3) | 0.4 (0.2) | 0.4 (0.1) |
| ((L/h)/kg) | CV (%) | NC | NA | 36.2 | 41.1 | 49.1 | 34.0 |
| V | Mean (SD) | NC (NC) | 0.4 (NA) | 0.5 (0.1) | 0.5 (0.1) | 0.8 (0.7) | 0.3 (0.2) |
| (L/kg) | CV (%) | NC | NA | 11.6 | 15.0 | 92.2 | 43.9 |
| $t_{max}$ | Min | 0.25 | 0.50 | 0.75 | 0.75 | 0.57 | 0.75 |
| (h) | Max | 0.77 | 0.75 | 0.80 | 0.80 | 1.10 | 0.90 |

NA : Not Applicable, NC : Not Calculated

[a] n=0 for $t_{1/2}$; [b] n=2 for $t_{1/2}$; [c] n=4 for $t_{1/2}$; [d] n=3 for $t_{1/2}$; [e] n=5 for $t_{1/2}$

**Table S12 Summary Statistics of Plasma Pharmacokinetic Parameters of Baseline-Adjusted Thiamine After Single Ascending Dose of IV Infusion of RJX in Healthy Adult and Elderly Volunteers**

| | | Day 1 (Single Ascending Dose of RJX) | | | | | |
|---|---|---|---|---|---|---|---|
| | | Cohort 1 | Cohort 2 | Cohort 3 | Cohort 4 | Cohort 5 | Cohort 6 |
| Parameter | | RJX 0.024 mL/kg (0.060 mg/kg of Thiamine) in Healthy Subjects (n=6)[a] | RJX 0.076 mL/kg (0.189 mg/kg of Thiamine) in Healthy Subjects (n=6)[b] | RJX 0.240 mL/kg (0.598 mg/kg of Thiamine) in Healthy Subjects (n=6) | RJX 0.500 mL/kg (1.246 mg/kg of Thiamine) in Healthy Subjects (n=6)[b] | RJX 0.759 mL/kg (1.891 mg/kg of Thiamine) in Healthy Subjects (n=6) | RJX 0.500 mL/kg (1.246 mg/kg of Thiamine) in Elderly (n=9) |
| $C_{max}$ | Mean (SD) | 46.89 (15.72) | 280.79 (77.24) | 887.69 (150.60) | 2468.83 (352.15) | 3695.59 (958.75) | 3108.77 (378.29) |
| (ng/mL) | CV (%) | 33.5 | 27.5 | 17.0 | 14.3 | 25.9 | 12.2 |
| $AUC_{0\text{-last}}$ | Mean (SD) | 53.71 (23.41) | 300.06 (88.33) | 1164.07 (234.10) | 3432.48 (291.45) | 5367.91 (1154.18) | 4393.07 (961.61) |
| (h*ng/mL) | CV (%) | 43.6 | 29.4 | 20.1 | 8.5 | 21.5 | 21.9 |
| $t_{1/2}$ | Mean (SD) | 1.99 (2.46) | 2.59 (1.55) | 10.41 (6.00) | 7.54 (0.71) | 7.98 (2.44) | 8.40 (1.74) |
| (h) | CV (%) | 123.8 | 59.8 | 57.6 | 9.4 | 30.6 | 20.7 |
| Cl | Mean (SD) | 1.02 (0.33) | 0.69 (0.26) | 0.52 (0.11) | 0.35 (0.03) | 0.37 (0.10) | 0.29 (0.06) |
| ((L/h)/kg) | CV (%) | 32.5 | 37.6 | 21.2 | 7.7 | 27.1 | 20.7 |
| V | Mean (SD) | 2.12 (1.70) | 2.18 (0.68) | 8.06 (5.44) | 3.85 (0.45) | 4.07 (1.17) | 3.58 (1.20) |
| (L/kg) | CV (%) | 80.2 | 31.2 | 67.4 | 11.7 | 28.8 | 33.4 |
| $t_{max}$ | Min | 0.50 | 0.50 | 0.75 | 0.75 | 0.57 | 0.75 |
| (h) | Max | 0.77 | 0.75 | 0.80 | 0.80 | 1.10 | 0.90 |

[a] n=4 for $t_{1/2}$ ; [b] n=5 for $t_{1/2}$

**Table S13 Summary Statistics of Plasma Pharmacokinetic Parameters of Baseline-Adjusted Ascorbic Acid After Single and Multiple Ascending Dose of IV Infusion of RJX in Healthy Adult Volunteers**

| | | Day 1 (Single Ascending Dose of RJX) | | | Day 7 (Multiple Ascending Dose of RJX) | | |
|---|---|---|---|---|---|---|---|
| | | Cohort 1 | Cohort 2 | Cohort 3 | Cohort 1 | Cohort 2 | Cohort 3 |
| Parameter | | RJX 0.240 mL/kg (10.792mg/kg of Ascorbic Acid) (n=6) | RJX 0.500 mL/kg (22.483 mg/kg of Ascorbic Acid) (n=6) | RJX 0.759 mL/kg (34.130 mg/kg of Ascorbic Acid (n=6) | RJX 0.240 mL/kg (10.792 mg/kg of Ascorbic Acid) (n=6) | RJX 0.500 mL/kg (22.483 mg/kg of Ascorbic Acid) (n=6) | RJX 0.759 mL/kg (34.130 mg/kg of Ascorbic Acid) (n=6) |
| $C_{max}$ | Mean (SD) | 51.10 (7.51) | 112.70 (14.31) | 166.52 (19.41) | 53.32 (11.06) | 120.90 (12.61) | 204.96 (23.18) |
| (µg/mL) | CV (%) | 14.7 | 12.7 | 11.7 | 20.7 | 10.4 | 11.3 |
| $AUC_{0-12}$ | Mean (SD) | 156.54 (25.92) | 271.03 (27.08) | 436.27 (53.66) | 124.50 (13.87) | 331.03 (43.19) | 531.77 (88.53) |
| (h*µg/mL) | CV (%) | 16.6 | 10.0 | 12.3 | 11.1 | 13.0 | 16.6 |
| $AUC_{0-last}$ | Mean (SD) | 156.54 (25.92) | 324.23 (39.52) | 524.55 (86.68) | NA | NA | NA |
| (h*µg/mL) | CV (%) | 16.6 | 12.2 | 16.5 | NA | NA | NA |
| $t_{1/2}$ | Mean (SD) | 3.52 (1.42) | 34.66 (NC) | 29.85 (NC) | NA | NA | NA |
| (h) | CV (%) | 40.3 | NC | NC | NA | NA | NA |
| Cl | Mean (SD) | 0.06 (0.02) | 0.04 (NC) | 0.03 (NC) | 0.08 (0.01) | 0.06 (NA) | 0.05 (NA) |
| ((L/h)/kg) | CV (%) | 24.3 | NC | NC | 10.7 | NA | NA |
| V | Mean (SD) | 0.31 (0.08) | 1.94 (NC) | 1.43 (NC) | 0.29 (0.06) | 1.51 (NA) | 3.41 (NA) |
| (L/kg) | CV (%) | 25.7 | NC | NC | 21.8 | NA | NA |
| $t_{max}$ | Min | 0.75 | 0.75 | 0.50 | 0.75 | 0.75 | 0.75 |
| (h) | Max | 0.77 | 0.75 | 0.77 | 1.47 | 0.75 | 0.80 |

**Table S14 Summary Statistics of Plasma Pharmacokinetic Parameters of Baseline-Adjusted Cyanocobalamin After Single and Multiple Ascending Dose of IV Infusion of RJX in Healthy Adult Volunteers**

| | | Day 1 (Single Ascending Dose of RJX) | | | Day 7 (Multiple Ascending Dose of RJX) | | |
|---|---|---|---|---|---|---|---|
| | | Cohort 1 | Cohort 2 | Cohort 3 | Cohort 1 | Cohort 2 | Cohort 3 |
| Parameter | | RJX 0.240 mL/kg (0.023 mg/kg of Cyanocobalamin) in Healthy Subjects (n=6) | RJX 0.500 mL/kg (0.048 mg/kg of Cyanocobalamin) in Healthy Subjects (n=6) | RJX 0.759 mL/kg (0.073 mg/kg of Cyanocobalamin) in Healthy Subjects (n=6) | RJX 0.240 mL/kg (0.023 mg/kg of Cyanocobalamin) in Healthy Subjects (n=6) | RJX 0.500 mL/kg (0.048 mg/kg of Cyanocobalamin) in Healthy Subjects (n=6)[a] | RJX 0.759 mL/kg (0.073 mg/kg of Cyanocobalamin) in Healthy Subjects (n=6) |
| $C_{max}$ (ng/mL) | Mean (SD)<br>CV (%) | 114.572 (13.026)<br>11.4 | 278.088 (41.173)<br>14.8 | 379.213 (49.753)<br>13.1 | 133.060 (23.018)<br>17.3 | 264.051 (30.942)<br>11.7 | 395.782 (47.718)<br>12.1 |
| $AUC_{0-12}$ (h*ng/mL) | Mean (SD)<br>CV (%) | 313.084 (13.976)<br>4.5 | 605.606 (56.374)<br>9.3 | 913.259 (87.995)<br>9.6 | 347.938 (32.154)<br>9.2 | 622.783 (51.232)<br>8.2 | 965.143 (77.843)<br>8.1 |
| $AUC_{0-last}$ (h*ng/mL) | Mean (SD)<br>CV (%) | 313.094 (13.984)<br>4.5 | 632.991 (62.623)<br>9.9 | 957.728 (94.345)<br>9.9 | NA<br>NA | NA<br>NA | NA<br>NA |
| $t_{1/2}$ (h) | Mean (SD)<br>CV (%) | 3.26 (1.19)<br>36.6 | 5.76 (0.90)<br>15.6 | 5.51 (1.16)<br>21.1 | NA<br>NA | NA<br>NA | NA<br>NA |
| Cl ((L/h)/kg) | Mean (SD)<br>CV (%) | 0.070 (0.003)<br>3.9 | 0.076 (0.007)<br>9.5 | 0.076 (0.008)<br>10.7 | 0.061 (0.006)<br>9.6 | 0.073 (0.007)<br>8.9 | 0.072 (0.007)<br>9.5 |
| V (L/kg) | Mean (SD)<br>CV (%) | 0.331 (0.119)<br>36.1 | 0.629 (0.118)<br>18.7 | 0.612 (0.188)<br>30.8 | 0.735 (0.040)<br>5.5 | 0.582 (0.160)<br>27.5 | 0.583 (0.141)<br>24.1 |
| $t_{max}$ (h) | Min<br>Max | 0.75<br>0.77 | 3.082<br>5.403 | 3.554<br>8.570 | 0.75<br>1.47 | 0.75<br>0.75 | 0.50<br>0.80 |

**Table S15 Summary Statistics of Serum Pharmacokinetic Parameters of Baseline-Adjusted Magnesium After Single and Multiple Ascending Dose of IV Infusion of RJX in Healthy Adult Volunteers**

| | | Day 1 (Single Ascending Dose of RJX) | | | Day 7 (Multiple Ascending Dose of RJX) | | |
|---|---|---|---|---|---|---|---|
| | | Cohort 1 | Cohort 2 | Cohort 3 | Cohort 1 | Cohort 2 | Cohort 3 |
| Parameter | | RJX 0.240 mL/kg (0.956 mg/kg of Magnesium) in Healthy Subjects (n=6) | RJX 0.500 mL/kg (1.992 mg/kg of Magnesium) in Healthy Subjects (n=6) | RJX 0.759 mL/kg (3.024 mg/kg of Magnesium) in Healthy Subjects (n=6) | RJX 0.240 mL/kg (0.956 mg/kg of Magnesium) in Healthy Subjects (n=6) | RJX 0.500 mL/kg (1.992 mg/kg of Magnesium) in Healthy Subjects (n=6) | RJX 0.759 mL/kg (3.024 mg/kg of Magnesium) in Healthy Subjects (n=6) |
| $C_{max}$ | Mean (SD) | 0.455 (0.139) | 0.967 (0.174) | 1.543 (0.154) | 0.466 (0.063) | 1.127 (0.182) | 1.596 (0.159) |
| (mg/dL) | CV (%) | 30.6 | 18.0 | 10.0 | 13.6 | 16.1 | 10.0 |
| $AUC_{0-12}$ | Mean (SD) | 3.008 (1.163) | 4.634 (1.030) | 6.695 (0.985) | 1.964 (0.854) | 6.017 (1.239) | 5.076 (1.365) |
| (h*mg/dL) | CV (%) | 38.7 | 22.2 | 14.7 | 43.5 | 20.6 | 26.9 |
| $AUC_{0-last}$ | Mean (SD) | 3.009 (1.165) | 6.570 (2.744) | 9.324 (1.236) | NA | NA | NA |
| (h*mg/dL) | CV (%) | 38.7 | 41.8 | 13.3 | NA | NA | NA |
| $t_{1/2}$ | Mean (SD) | NC (NC) | 7.72 (NA) | 20.28 (18.26) | NA | NA | NA |
| (h) | CV (%) | NC | NA | 90.0 | NA | NA | NA |
| Cl | Mean (SD) | NC (NC) | 0.028 (NA) | 0.020 (0.005) | 0.034 (NC) | 0.024 (NA) | 0.046 (0.008) |
| ((L/h)/kg) | CV (%) | NC | NA | 23.4 | NC | NA | 18.2 |
| V | Mean (SD) | NC (NC) | 0.310 (NA) | 0.501 (0.267) | 0.137 (NC) | 0.294 (NA) | 0.265 (0.058) |
| (L/kg) | CV (%) | NC | NA | 53.2 | NC | NA | 21.8 |
| $t_{max}$ | Min | 0.062 | 0.75 | 0.133 | 0.50 | 0.75 | 0.75 |
| (h) | Max | 0.409 | 0.75 | 0.391 | 1.75 | 0.75 | 0.80 |

**Table S16 Summary Statistics of Plasma Pharmacokinetic Parameters of Baseline-Adjusted Niacinamide After Single and Multiple Ascending Dose of IV Infusion of RJX in Healthy Adult Volunteers**

| | | Day 1 (Single Ascending Dose of RJX) | | | Day 7 (Multiple Ascending Dose of RJX) | | |
|---|---|---|---|---|---|---|---|
| | | Cohort 1 | Cohort 2 | Cohort 3 | Cohort 1 | Cohort 2 | Cohort 3 |
| Parameter | | RJX 0.240 mL/kg (1.426 mg/kg of Niacinamide) in Healthy Subjects (n=6) | RJX 0.500 mL/kg (2.970 mg/kg of Niacinamide) in Healthy Subjects (n=6) | RJX 0.759 mL/kg (4.508 mg/kg of Niacinamide) in Healthy Subjects (n=6) | RJX 0.240 mL/kg (1.426 mg/kg of Niacinamide) in Healthy Subjects (n=6) | RJX 0.500 mL/kg (2.970 mg/kg of Niacinamide) in Healthy Subjects (n=6) | RJX 0.759 mL/kg (4.508 mg/kg of Niacinamide) in Healthy Subjects (n=6) |
| $C_{max}$ | Mean (SD) | 1554.5 (464.5) | 4891.6 (1014.6) | 7735.1 (811.0) | 1584.0 (417.7) | 4611.9 (1114.6) | 8145.5 (571.3) |
| (ng/mL) | CV (%) | 29.9 | 20.7 | 10.5 | 26.4 | 24.2 | 7.0 |
| $AUC_{0-12}$ | Mean (SD) | 1857.1 (558.8) | 6322.7 (1435.7) | 11245.1 (1876.6) | 1939.4 (542.6) | 6845.7 (1430.9) | 12349.8 (1686.3) |
| (h*ng/mL) | CV (%) | 30.1 | 22.7 | 16.7 | 28.0 | 20.9 | 13.7 |
| $AUC_{0-last}$ | Mean (SD) | 1774.3 (452.2) | 6305.0 (1418.2) | 11228.3 (1815.1) | NA | NA | NA |
| (h*ng/mL) | CV (%) | 25.5 | 22.5 | 16.2 | NA | NA | NA |
| $t_{1/2}$ | Mean (SD) | 0.57 (0.33) | 0.58 (0.27) | 0.70 (0.35) | NA | NA | NA |
| (h) | CV (%) | 58.1 | 46.3 | 50.7 | NA | NA | NA |
| Cl | Mean (SD) | 0.9 (0.4) | 0.5 (0.2) | 0.4 (0.1) | 0.8 (0.3) | 0.5 (0.1) | 0.3 (0.0) |
| ((L/h)/kg) | CV (%) | 42.3 | 32.0 | 16.1 | 39.6 | 28.5 | 11.0 |
| V | Mean (SD) | 0.6 (0.3) | 0.4 (0.3) | 0.4 (0.2) | 1.2 (1.4) | 0.3 (0.1) | 0.3 (0.1) |
| (L/kg) | CV (%) | 52.6 | 69.0 | 41.0 | 109.9 | 22.6 | 22.6 |
| $t_{max}$ | Min | 0.75 | 0.75 | 0.75 | 0.75 | 0.75 | 0.50 |
| (h) | Max | 0.77 | 0.75 | 0.77 | 1.47 | 0.75 | 0.80 |

**Table S17 Summary Statistics of Plasma Pharmacokinetic Parameters of Baseline-Adjusted Thiamine After Single and Multiple Ascending Dose of IV Infusion of RJX in Healthy Adult Volunteers**

| | | Day 1 (Single Ascending Dose of RJX) | | | Day 7 (Multiple Ascending Dose of RJX) | | |
|---|---|---|---|---|---|---|---|
| | | Cohort 1 | Cohort 2 | Cohort 3 | Cohort 1 | Cohort 2 | Cohort 3 |
| Parameter | | RJX 0.240 mL/kg (0.598 mg/kg of Thiamine) in Healthy Subjects (n=6) | RJX 0.500 mL/kg (1.246 mg/kg of Thiamine) in Healthy Subjects (n=6) | RJX 0.759 mL/kg (1.891 mg/kg of Thiamine) in Healthy Subjects (n=6) | RJX 0.240 mL/kg (0.598 mg/kg of Thiamine) in Healthy Subjects (n=6) | RJX 0.500 mL/kg (1.246 mg/kg of Thiamine) in Healthy Subjects (n=6)[a] | RJX 0.759 mL/kg (1.891 mg/kg of Thiamine) in Healthy Subjects (n=6)[b] |
| $C_{max}$ | Mean (SD) | 980.04 (206.67) | 2765.17 (409.76) | 4527.05 (646.70) | 1160.17 (262.00) | 2725.79 (495.77) | 4728.21 (759.21) |
| (ng/mL) | CV (%) | 21.1 | 14.8 | 14.3 | 22.6 | 18.2 | 16.1 |
| $AUC_{0-12}$ | Mean (SD) | 1266.44 (247.23) | 3608.88 (730.57) | 5681.88 (877.99) | 1591.73 (290.06) | 3802.97 (552.19) | 6086.58 (1034.46) |
| (h*ng/mL) | CV (%) | 19.5 | 20.2 | 15.5 | 18.2 | 14.5 | 17.0 |
| $AUC_{0-last}$ | Mean (SD) | 1266.46 (247.25) | 3683.37 (767.06) | 5759.70 (907.59) | NA | NA | NA |
| (h*ng/mL) | CV (%) | 19.5 | 20.8 | 15.8 | NA | NA | NA |
| $t_{1/2}$ | Mean (SD) | 3.46 (2.61) | 8.65 (1.82) | 8.48 (1.60) | NA | NA | NA |
| (h) | CV (%) | 75.3 | 21.0 | 18.9 | NA | NA | NA |
| Cl | Mean (SD) | 0.48 (0.11) | 0.34 (0.06) | 0.33 (0.05) | 0.32 (0.03) | 0.32 (0.04) | 0.31 (0.06) |
| ((L/h)/kg) | CV (%) | 22.2 | 17.2 | 14.0 | 10.4 | 14.1 | 30.1 |
| V | Mean (SD) | 2.16 (1.22) | 4.35 (1.38) | 4.09 (1.20) | 6.89 (0.65) | 5.55 (1.58) | 4.08 (1.40) |
| (L/kg) | CV (%) | 56.5 | 31.7 | 29.3 | 9.5 | 28.5 | 34.4 |
| $t_{max}$ | Min | 0.5 | 0.75 | 0.50 | 0.75 | 0.75 | 0.50 |
| (h) | Max | 0.77 | 0.75 | 0.75 | 1.47 | 0.75 | 0.80 |

**Table S18. Predicted ΔΔQTcF Interval At Geometric Mean Peak Concentration For Ascorbic Acid And Magnesium (SAD and MAD) (PK/QTc Analysis Set)**

| Treatment | Geometric Mean (mg/L) (90% CI) $C_{max}$ of ascorbic acid | Geometric Mean (mg/L) (90% CI) $C_{max}$ of magnesium | ΔΔQTcF Estimate (ms) (90% CI) |
|---|---|---|---|
| SAD 0.024 mL/kg | 13.0 (9.19; 18.27) | 18.0 (17.20; 18.87) | 1.28 (-0.84, 3.40) |
| SAD 0.076 mL/kg | 26.2 (23.98; 28.71) | 20.5 (18.90; 22.29) | 2.30 (0.21, 4.38) |
| SAD 0.240 mL/kg | 54.8 (45.18; 66.51) | 21.2 (20.55; 21.78) | 2.84 (0.76, 4.92) |
| SAD 0.5 mL/kg | 117.9 (105.54; 131.65) | 24.8 (23.24; 26.51) | 4.83 (2.50, 7.16) |
| SAD Elderly 0.5 mL/kg | 141.4 (129.32; 154.64) | 27.3 (25.25; 29.54) | 5.96 (3.51, 8.41) |
| SAD 0.759 mL/kg | 158.4 (134.35; 186.78) | 30.1 (27.78; 32.69) | 7.12 (4.54, 9.70) |
| MAD 0.240 mL/kg | 66.1 (57.71; 75.64) | 23.0 (21.48; 24.55) | 3.59 (1.50, 5.68) |
| MAD 0.5 mL/kg | 129.9 (116.18; 145.16) | 29.1 (28.23; 30.06) | 6.45 (4.03, 8.86) |
| MAD 0.759 mL/kg | 211.2 (193.85; 230.02) | 34.2 (32.71; 35.79) | 9.13 (6.08, 12.19) |

**Table S19. Predicted ΔΔQTcF Interval At Geometric Mean Peak Concentration For Ascorbic Acid (SAD And MAD) (PK/QTc Analysis Set)**

| Treatment | Geometric Mean (mg/L) (90% CI) $C_{max}$ of ascorbic acid | ΔΔQTcF Estimate (ms) (90% CI) |
|---|---|---|
| SAD 0.024 mL/kg | 13.0 (9.19; 18.27) | 1.51 (-0.90, 3.91) |
| SAD 0.076 mL/kg | 26.2 (23.98; 28.71) | 2.08 (-0.28, 4.44) |
| SAD 0.240 mL/kg | 54.8 (45.18; 66.51) | 3.31 (1.00, 5.62) |
| SAD 0.5 mL/kg | 117.9 (105.54; 131.65) | 6.03 (3.51, 8.55) |
| SAD Elderly 0.5 mL/k | 141.4 (129.32; 154.64) | 7.05 (4.35, 9.74) |
| SAD 0.759 mL/kg | 158.4 (134.35; 186.78) | 7.78 (4.93, 10.63) |
| MAD 0.240 mL/kg | 66.1 (57.71; 75.64) | 3.80 (1.48, 6.12) |
| MAD 0.5 mL/kg | 129.9 (116.18; 145.16) | 6.55 (3.94, 9.15) |
| MAD 0.759 mL/kg | 211.2 (193.85; 230.02) | 10.05 (6.63, 13.48) |
| 10 ms Threshold | 147 | 7.29 (4.54, 10.03) |

Based on a linear mixed-effects model with ΔQTcF as the dependent variable, time-matched ascorbic acid plasma concentration as an explanatory variate, centered baseline as an additional covariate, treatment (active = 1 or placebo = 0) and time as fixed effects, and a random intercept and slope per subject.

**Table S20. Predicted ΔΔQTcF Interval At Geometric Mean Peak Concentration For Cyanocobalamin (SAD AND MAD) (PK/QTc Analysis Set)**

| Treatment | Geometric Mean (µg/L) (90% CI) $C_{max}$ of cyanocobalamin | ΔΔQTcF Estimate (ms) (90% CI) |
|---|---|---|
| SAD 0.024 mL/kg | 10.6 (9.42; 11.96) | 1.64 (-0.73, 4.01) |
| SAD 0.076 mL/kg | 41.9 (35.78; 49.05) | 2.20 (-0.13, 4.52) |
| SAD 0.240 mL/kg | 107.5 (91.86; 125.79) | 3.36 (1.08, 5.64) |
| SAD 0.5 mL/kg | 241.0 (221.56; 262.08) | 5.72 (3.26, 8.19) |
| SAD Elderly 0.5 mL/k | 323.9 (297.62; 352.49) | 7.19 (4.45, 9.93) |
| SAD 0.759 mL/kg | 362.1 (305.31; 429.50) | 7.87 (4.97, 10.77) |
| MAD 0.240 mL/kg | 131.1 (111.82; 153.82) | 3.78 (1.49, 6.06) |
| MAD 0.5 mL/kg | 262.4 (236.25; 291.37) | 6.10 (3.58, 8.63) |
| MAD 0.759 mL/kg | 393.4 (356.26; 434.47) | 8.43 (5.39, 11.46) |
| 10 ms Threshold | 328 | 7.27 (4.51, 10.02) |

Based on a linear mixed-effects model with ΔQTcF as the dependent variable, time-matched cyanocobalamin plasma concentration as an explanatory variate, centered baseline as an additional covariate, treatment (active = 1 or placebo = 0) and time as fixed effects, and a random intercept and slope per subject.

**Table S21. Predicted ΔΔQTcF Interval At Geometric Mean Peak Concentration For Magnesium (SAD AND MAD) (PK/QTc Analysis Set)**

| Treatment | Geometric Mean (mg/dL) (90% CI) $C_{max}$ of magnesium | ΔΔQTcF Estimate (ms) (90% CI) |
|---|---|---|
| SAD 0.024 mL/kg | 1.8 (1.72; 1.89) | 1.30 (-0.83, 3.43) |
| SAD 0.076 mL/kg | 2.1 (1.89; 2.23) | 2.43 (0.35, 4.51) |
| SAD 0.240 mL/kg | 2.1 (2.05; 2.18) | 2.71 (0.64, 4.79) |
| SAD 0.5 mL/kg | 2.5 (2.32; 2.65) | 4.37 (2.21, 6.52) |
| SAD Elderly 0.5 mL/k | 2.7 (2.53; 2.95) | 5.49 (3.21, 7.77) |
| SAD 0.759 mL/kg | 3.0 (2.78; 3.27) | 6.76 (4.27, 9.25) |
| MAD 0.240 mL/kg | 2.3 (2.15; 2.45) | 3.53 (1.43, 5.63) |
| MAD 0.5 mL/kg | 2.9 (2.82; 3.01) | 6.31 (3.90, 8.71) |
| MAD 0.759 mL/kg | 3.4 (3.27; 3.58) | 8.59 (5.71, 11.47) |
| 10 ms Threshold | 3 | 7.42 (4.80, 10.04) |

Based on a linear mixed-effects model with ΔQTcF as the dependent variable, time-matched magnesium plasma concentration as an explanatory variate, centered baseline as an additional covariate, treatment (active = 1 or placebo = 0) and time as fixed effects, and a random intercept per subject.

**Table S22. Predicted ΔΔQTcF Interval At Geometric Mean Peak Concentration For Niacinamide (SAD AND MAD) (PK/QTc Analysis Set)**

| Treatment | Geometric Mean (µg/L) (90% CI) $C_{max}$ of niacinamide | ΔΔQTcF Estimate (ms) (90% CI) |
|---|---|---|
| SAD 0.024 mL/kg | 114.4 (94.27; 138.96) | 1.99 (-0.34, 4.32) |
| SAD 0.076 mL/kg | 511.7 (390.97; 669.70) | 2.30 (0.00, 4.60) |
| SAD 0.240 mL/kg | 1282.3 (1022.38; 1608.39) | 2.91 (0.65, 5.17) |
| SAD 0.5 mL/kg | 3383.9 (2657.47; 4309.03) | 4.56 (2.24, 6.89) |
| SAD Elderly 0.5 mL/kg | 5694.3 (4773.12; 6793.26) | 6.38 (3.74, 9.03) |
| SAD 0.759 mL/kg | 6748.1 (5059.28; 9000.60) | 7.21 (4.35, 10.07) |
| MAD 0.240 mL/kg | 1569.2 (1242.00; 1982.62) | 3.13 (0.88, 5.39) |
| MAD 0.5 mL/kg | 4528.8 (3706.58; 5533.52) | 5.46 (3.01, 7.92) |
| MAD 0.759 mL/kg | 8151.0 (7695.03; 8634.04) | 8.32 (5.12, 11.51) |
| 10 ms Threshold | 6680 | 7.16 (4.31, 10.00) |

Based on a linear mixed-effects model with ΔQTcF as the dependent variable, time-matched niacinamide plasma concentration as an explanatory variate, centered baseline as an additional covariate, treatment (active = 1 or placebo = 0) and time as fixed effects, and a random intercept and slope per subject.

**Table S23. Predicted ΔΔQTcF INTERVAL At Geometric Mean Peak Concentration For Thiamine (SAD AND MAD) (PK/QTc Analysis Set)**

| Treatment | Geometric Mean (µg/L) (90% CI) $C_{max}$ of thiamine | ΔΔQTcF Estimate (ms) (90% CI) |
|---|---|---|
| SAD 0.024 mL/kg | 42.2 (28.26; 63.09) | 2.17 (-0.16, 4.49) |
| SAD 0.076 mL/kg | 269.9 (206.38; 353.05) | 2.42 (0.12, 4.72) |
| SAD 0.240 mL/kg | 875.0 (745.24; 1027.24) | 3.10 (0.83, 5.36) |
| SAD 0.5 mL/kg | 2449.6 (2187.76; 2742.79) | 4.85 (2.39, 7.32) |
| SAD Elderly 0.5 mL/kg | 3088.5 (2865.16; 3329.36) | 5.57 (2.91, 8.22) |
| SAD 0.759 mL/kg | 3596.5 (2920.21; 4429.46) | 6.13 (3.30, 8.97) |
| MAD 0.240 mL/kg | 1132.5 (921.39; 1392.10) | 3.38 (1.11, 5.65) |
| MAD 0.5 mL/kg | 2688.3 (2313.14; 3124.24) | 5.12 (2.59, 7.65) |
| MAD 0.759 mL/kg | 4676.1 (4081.25; 5357.60) | 7.34 (4.04, 10.64) |
| 10 ms Threshold | 4270 | 6.89 (3.77, 10.00) |

Based on a linear mixed-effects model with ΔQTcF as the dependent variable, time-matched thiamine plasma concentration as an explanatory variate, centered baseline as an additional covariate, treatment (active = 1 or placebo = 0) and time as fixed effects, and a random intercept and slope per subject.

# SUPPLEMENTAL FIGURES

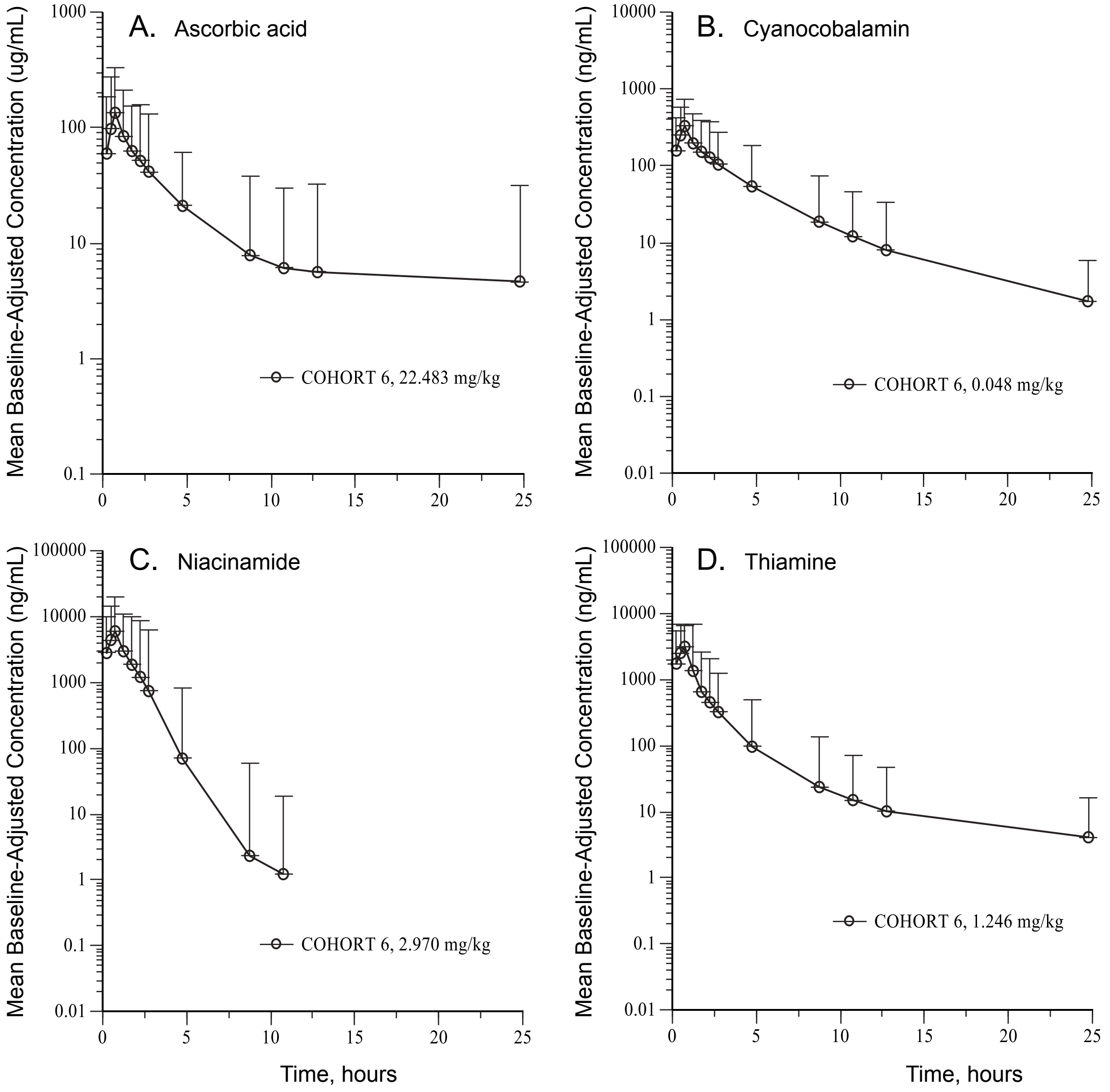


**Figure S1. Pharmacokinetics of the Vitamin Components of RJX.** Plasma concentration-time profiles of baseline-adjusted ascorbic acid (A), cyanocobalamin (B), niacinamide (C) and thiamine (D) for the RJX dose level of 0.5 mL/kg (Cohort 6) following single-dose IV infusion of RJX are presented on a semi-logarithmic scale

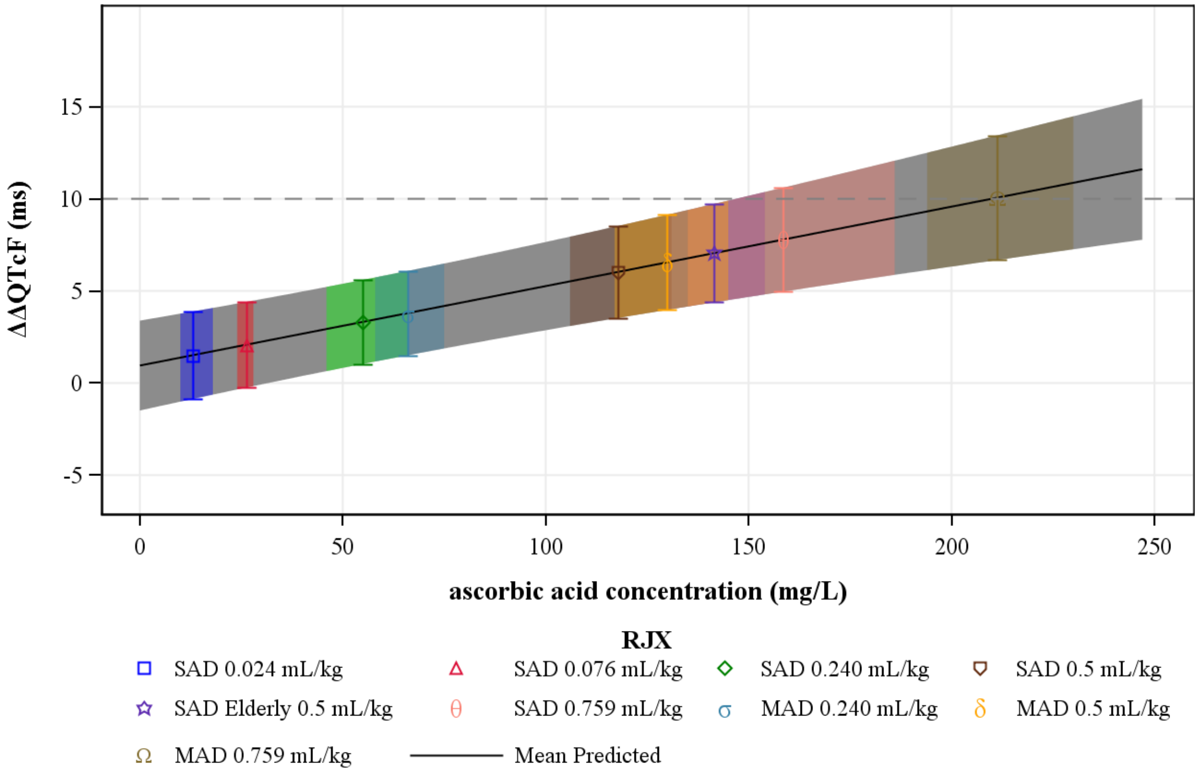


**Figure S2. Predicted ΔΔQTcF interval at geometric mean peak concentrations for ascorbic acid (SAD and MAD) (PK/QTc analysis set).** The solid black line with gray shaded area denotes the model-predicted mean (90% CI) ΔΔQTcF, which is calculated from the equation ΔΔQTcF = 0.95 + 0.043 × ascorbic acid. The non-gray shaded areas denote the estimated mean (90% CI) ΔΔQTcF with plotted points at the geometric mean (90% CI) $C_{max}$ of ascorbic acid.

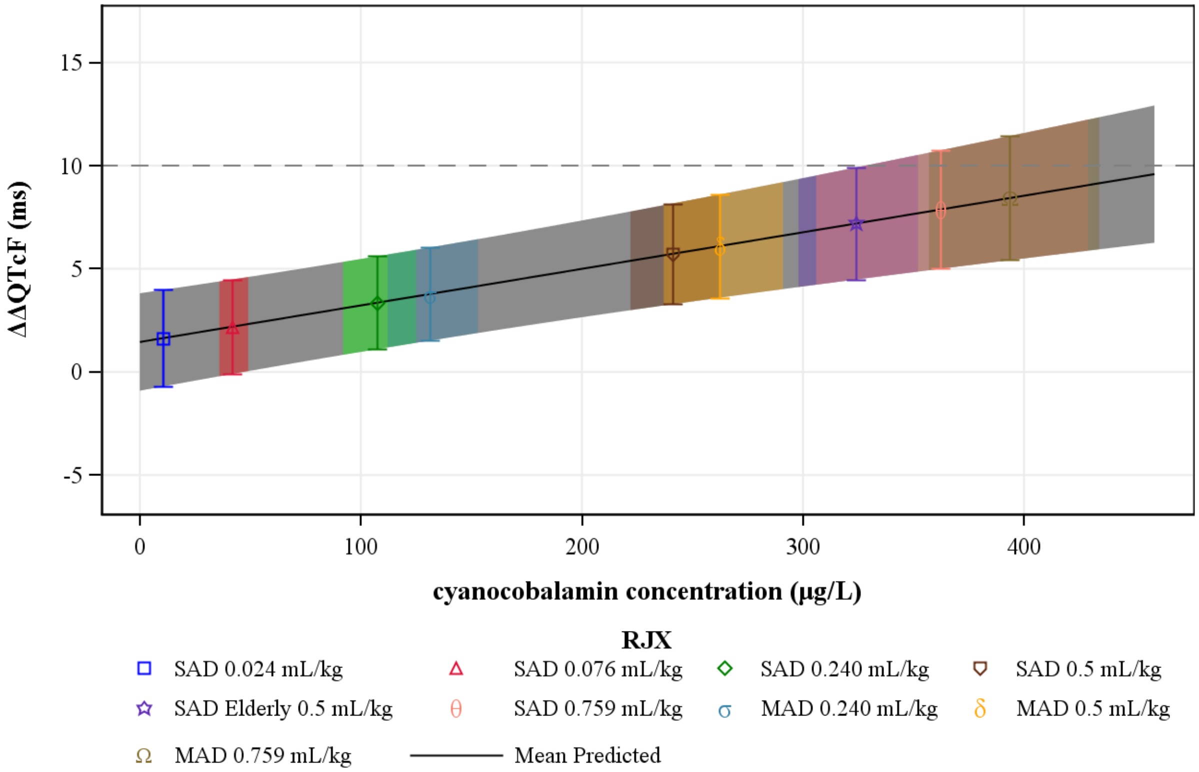


**Figure S3. Predicted ΔΔQTcF interval at geometric mean peak concentrations for cyanocobalamin (SAD and MAD) (PK/QTc analysis set).** The solid black line with gray shaded area denotes the model-predicted mean (90% CI) ΔΔQTcF, which is calculated from the equation ΔΔQTcF = 1.45 + 0.018 × cyanocobalamin. The non-gray shaded areas denote the estimated mean (90% CI) ΔΔQTcF with plotted points at the geometric mean (90% CI) $C_{max}$ of cyanocobalamin.

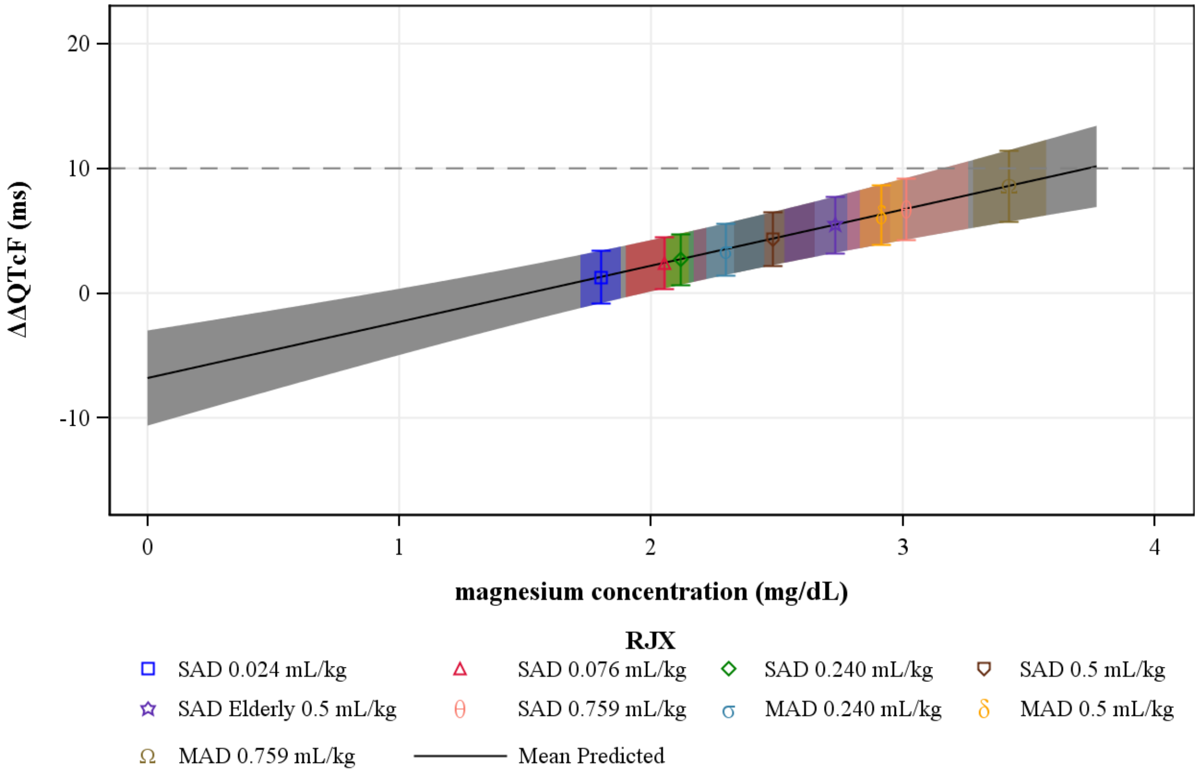


**Figure S4. Predicted ΔΔQTcF interval at geometric mean peak concentrations for magnesium (SAD and MAD) (PK/QTc analysis set).** The solid black line with gray shaded area denotes the model-predicted mean (90% CI) ΔΔQTcF, which is calculated from the equation ΔΔQTcF = −6.81 + 4.50 × magnesium. The non-gray shaded areas denote the estimated mean (90% CI) ΔΔQTcF with plotted points at the geometric mean (90% CI) $C_{max}$ of magnesium.

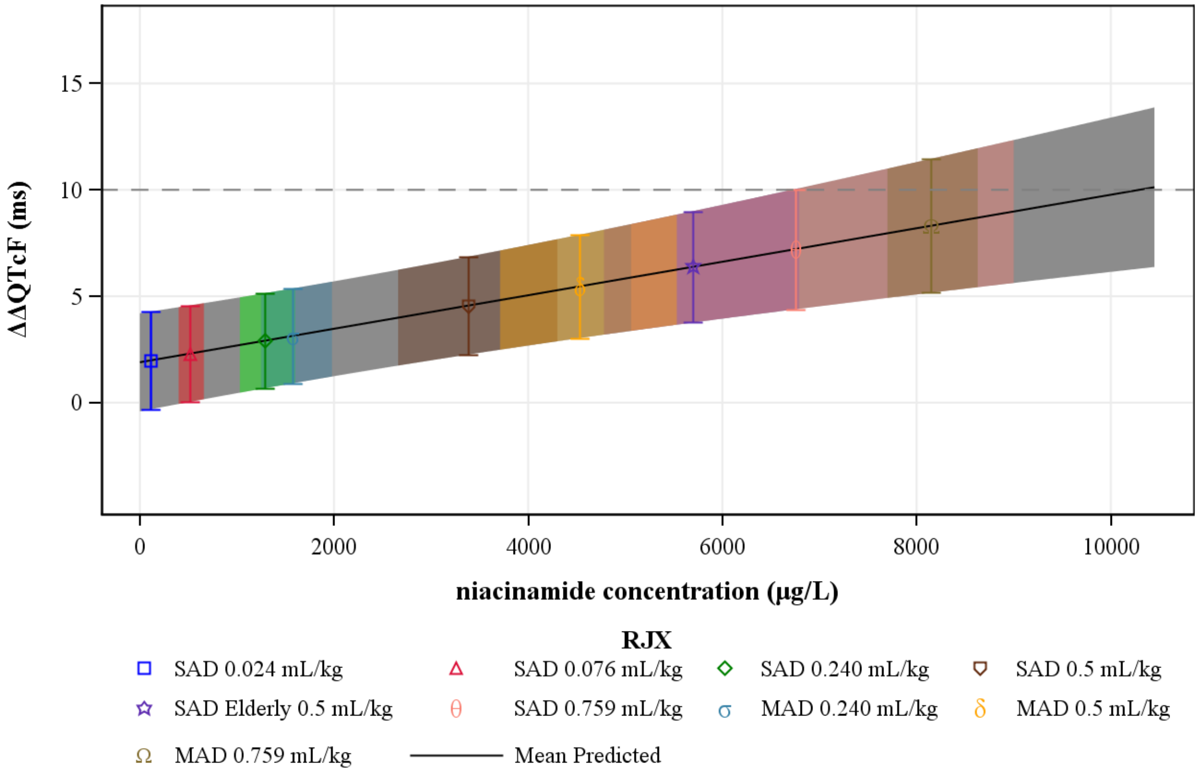


**Figure S5. Predicted ΔΔQTcF interval at geometric mean peak concentrations for niacinamide (SAD and MAD) (PK/QTc analysis set).** The solid black line with gray shaded area denotes the model-predicted mean (90% CI) ΔΔQTcF, which is calculated from the equation ΔΔQTcF = 1.90 + 0.00079 × niacinamide. The non-gray shaded areas denote the estimated mean (90% CI) ΔΔQTcF with plotted points at the geometric mean (90% CI) $C_{max}$ of niacinamide.

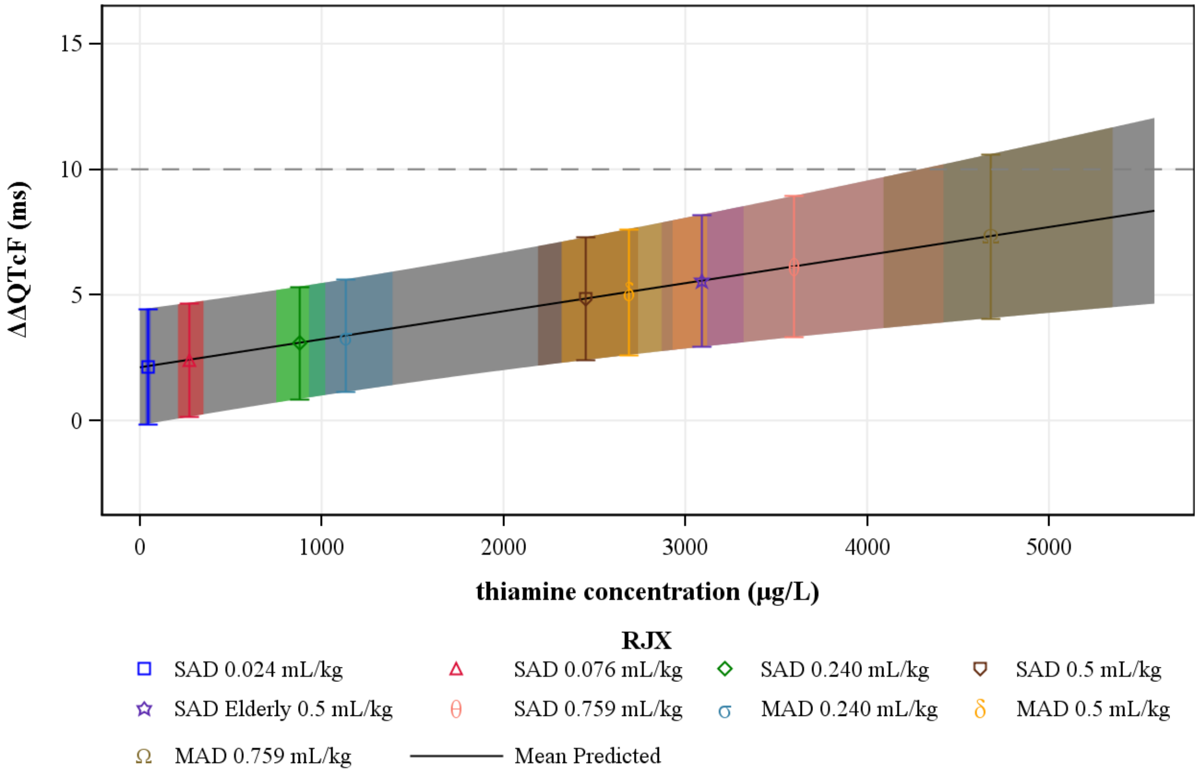


**Figure S6. Predicted ΔΔQTcF interval at geometric mean peak concentrations for thiamine (SAD and MAD) (PK/QTc analysis set.** The solid black line with gray shaded area denotes the model-predicted mean (90% CI) ΔΔQTcF, which is calculated from the equation ΔΔQTcF = 2.12 + 0.0011 × thiamine. The non-gray shaded areas denote the estimated mean (90% CI) ΔΔQTcF with plotted points at the geometric mean (90% CI) $C_{max}$ of thiamine.

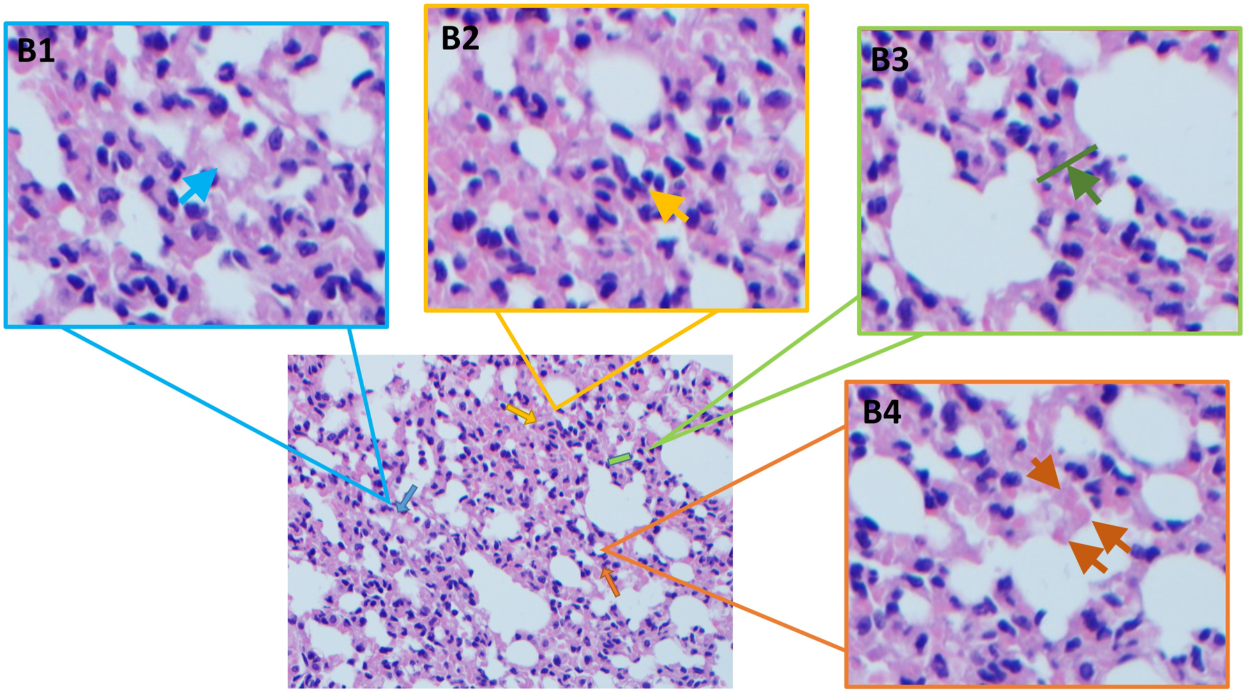


**Figure S7. Acute Lung Injury and Inflammation in the LPS-GalN Mouse Model of ARDS and Multi-organ Failure.** Depicted are enlarged insets from Figure 6B, which is shown in the left lower corner. Mice were challenged with 0.5 ml of LPS-GalN (consisting of 100 ng of LPS plus 8 mg of D-galactosamine) i.p. [Panel B1]. Blue arrow: Exudate; [Panel B2]. Yellow arrow: inflammatory cell infiltration; [Panel B3]. Green block: thickness of alveolar wall; [Panel B4]. Orange arrows: hemorrhage. H&E X400

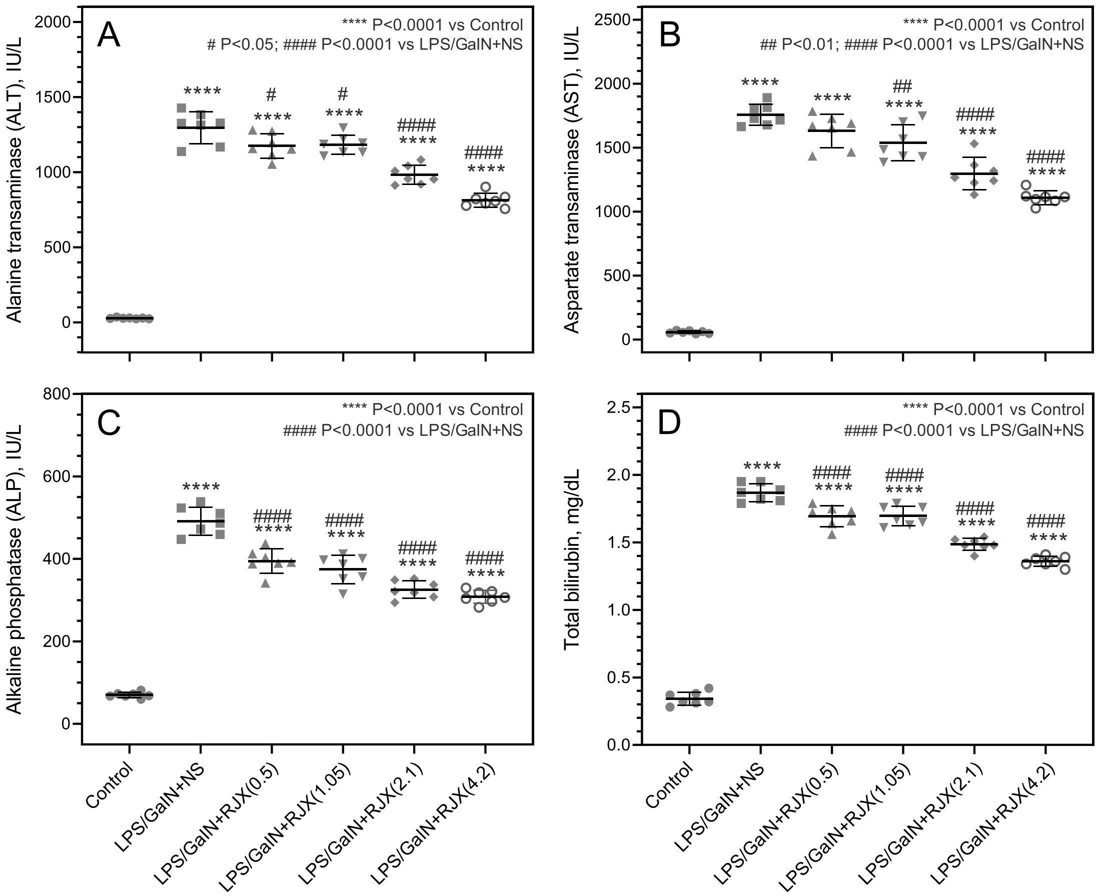


**Figure S8. Effect of Rejuveinix (RJX) on Hepatic Dysfunction in Mice Challenged with LPS-GalN.** Mice were treated with i.p injections of RJX (0.5, 1.05, 2.1, 4.2 mL/kg, 0.5 ml/mouse) or NS 2 hours before and 2 hours post-injection of LPS-GalN. Except for untreated mice (Control), each mouse received 0.5 ml of LPS-GalN (consisting of 100 ng of LPS plus 8 mg of D-galactosamine) i.p. The RJX dose levels (in ml/kg) are indicated in parentheses. See text for a discussion of results.  Results are expressed as the mean and standard deviation for the liver enzymes ALT, AST, Alk.Ptase and total bilirubin. Statistical significance between groups is shown by: *** P < 0.001; **** P < 0.0001 compared as control group and, # P < 0.05; ### P < 0.001; #### P < 0.0001 compared as LPS/GalN+NS group, ANOVA and Tukey's post-hoc test).

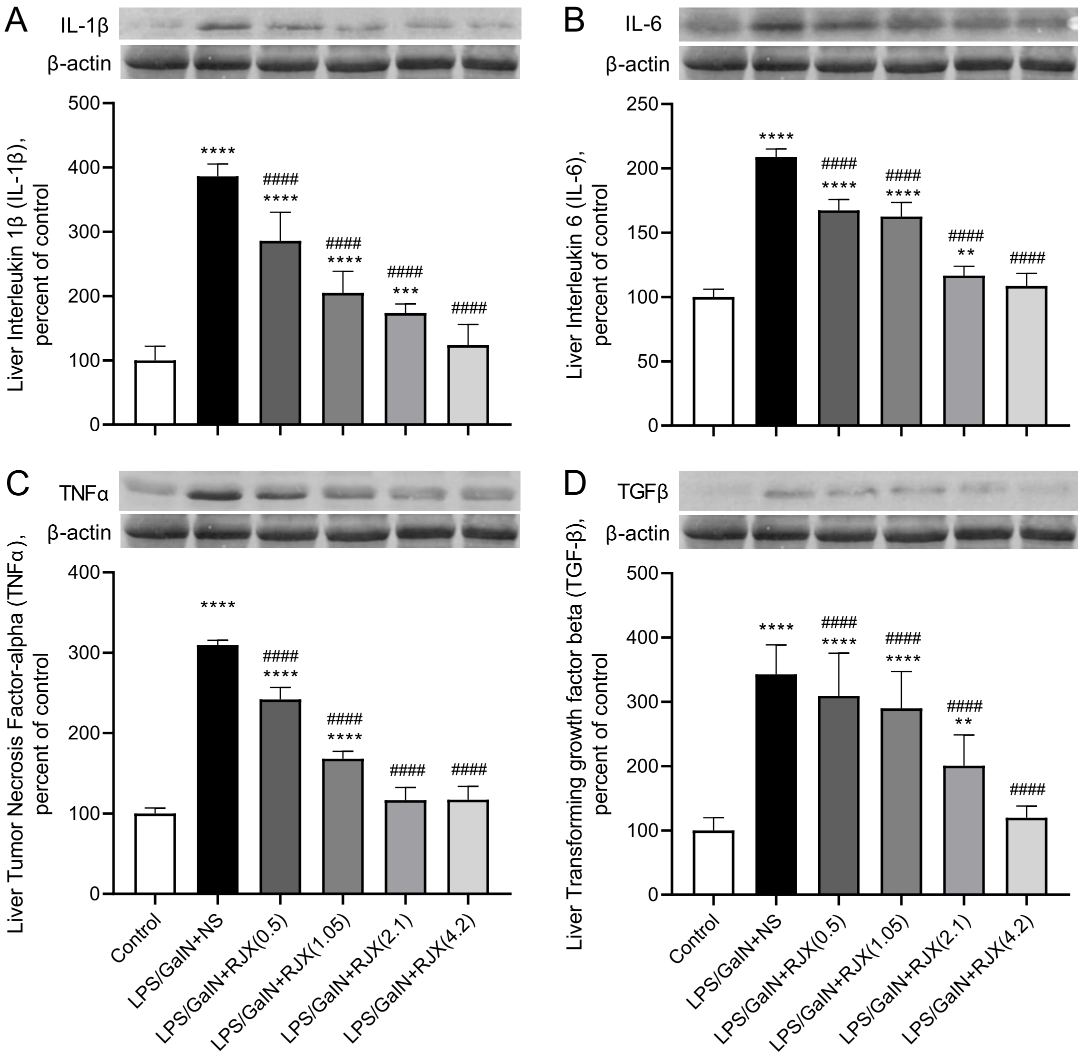


**Figure S9. Rejuveinix (RJX) Prevents Pro-inflammatory Cytokine Response in the Livers of Mice Challenged with LPS-GalN.** Mice were treated with i.p injections of RJX (0.5, 1.05, 2.1, 4.2 mL/kg, 0.5 ml/mouse) or NS 2 hours before and 2 hours post-injection of LPS-GalN. Except for untreated mice (Control), each mouse received 0.5 ml of LPS-GalN (consisting of 100 ng of LPS plus 8 mg of D-galactosamine) i.p. The RJX dose levels (in ml/kg) are indicated in parentheses. See text for discussion of results. Depicted are the results of Western blot analyses of cytokine expression in the pooled liver tissue samples from mice in various treatment groups. Results are expressed as percent of control with the expression level in the lung tissue sample from untreated control mice taken as 100% for comparisons. The bar represents mean and standard deviation. Immunoblotting with an anti-actin antibody was used to ensure equal protein loading. (ANOVA and Tukey's post-hoc test. Statistical significance between groups is shown by: *** P < 0.001; **** P < 0.0001 compared as control group and, # P < 0.05; ### P < 0.001; #### P < 0.0001 compared as LPS/GalN+NS group).

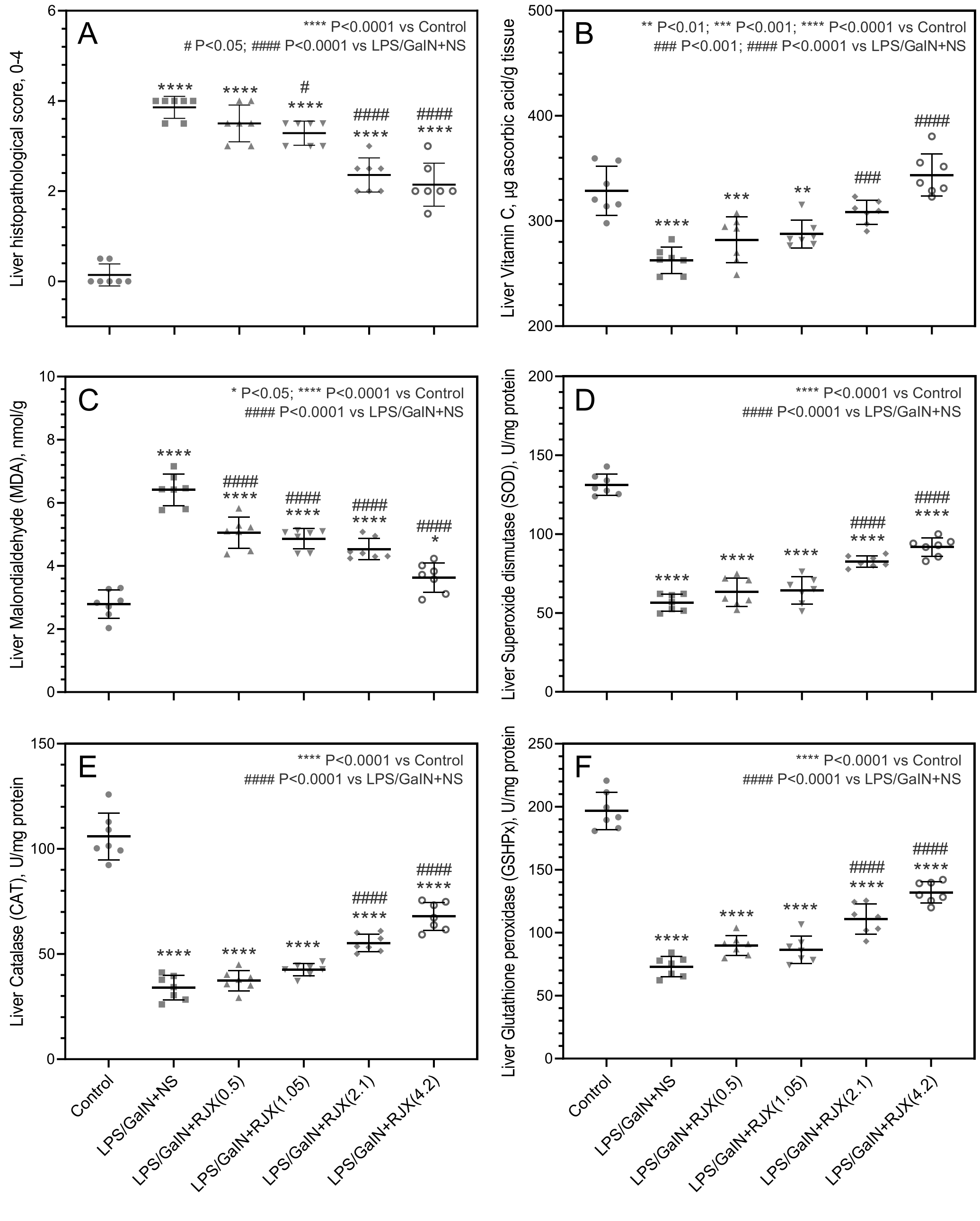


**Figure S10. Tissue-Level In Vivo Anti-Inflammatory and Anti-Oxidant Activity of Rejuveinix (RJX) in the LPS-GalN Mouse Model of ARDS and Multi-organ Failure.** Mice were treated with i.p injections of RJX (0.5, 1.05, 2.1, 4.2 mL/kg, 0.5 ml/mouse) or NS 2 hours before and 2 hours post-injection of LPS-GalN. Except for untreated mice (Control), each mouse received 0.5 ml of LPS-GalN (consisting of 100 ng of LPS plus 8 mg of D-galactosamine) i.p. The RJX dose levels (in ml/kg) are indicated in parentheses. See text for discussion of results. The depicted lines represent the mean and standard deviation for the indicated parameters. In [A], the liver histopathological score ("livery injury score") was graded according to a 5-point scale from 0 to 4 as follows: 0, l, 2, 3 and 4 represented no damage, mild damage, moderate damage, severe damage and very severe damage, respectively. Statistical significance between groups is shown by: **** P < 0.0001 compared as control group and, # P < 0.05; #### P < 0.0001 compared as LPS/GalN+NS group, Kruskal-Wallis test and Mann Whitney test. In [B]-[F], ANOVA and Tukey's post-hoc test were used for

comparing the results among different treatment groups. Statistical significance between groups is shown by: **** P < 0.0001 compared as control group and, # P < 0.05; ## P < 0.01; #### P < 0.0001 compared as LPS/GalN+NS group).

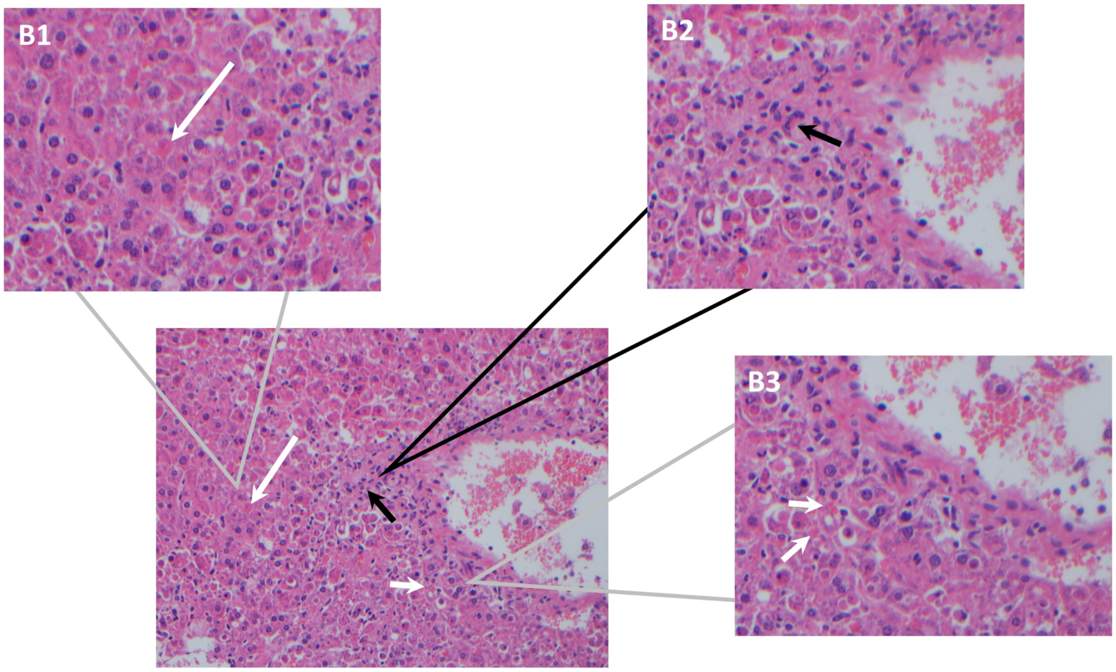


**Figure S11. Acute Liver Injury and Inflammation in the LPS-GalN Mouse Model of ARDS and Multi-organ Failure.** Depicted are enlarged insets from Figure 7B, which is shown in the left lower corner. Mice were challenged with 0.5 ml of LPS-GalN (consisting of 100 ng of LPS plus 8 mg of D-galactosamine) i.p. [Panel B1]. White arrow (long): necrosis; [Panel B2]. Black arrow: inflammatory cell infiltration; [Panel B3]. White arrow (short): hemorrhage. H&E X400

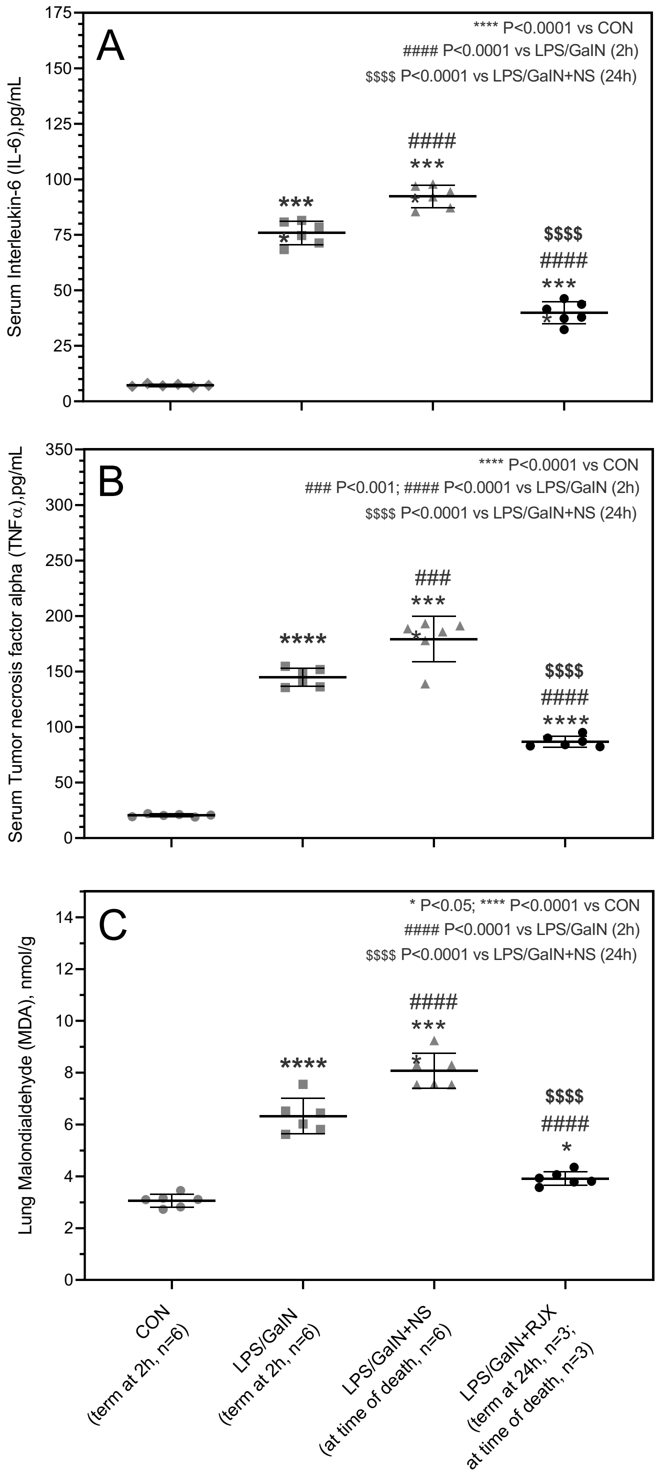


**Figure S12. Effect of Rejuveinix (RJX) on serum Interleukin-6 (IL-6; Panel A), Tumor necrosis factor alpha (TNFα; Panel B) and Lung malondialdehyde (MDA; Panel C) Mice Challenged with LPS-GalN.** Mice were treated with i.p injections of RJX (4.2 mL/kg, 0.5 ml/mouse) or NS 2 hours and 3 hours post-injection of LPS-GalN. Except for untreated mice (Control), each mouse received 0.5 ml of LPS-GalN (consisting of 100 ng of LPS plus 8 mg of D-galactosamine) i.p. The RJX dose levels (in ml/kg) are indicated in parentheses. Results are expressed as the mean and standard deviation Statistical significance between groups is shown by: ** P < 0.001; ***

P < 0.001; **** P < 0.0001 compared as LPS-GalN and #### P < 0.0001 compared as LPS/GaIN+NS group, ANOVA and Tukey's post-hoc test).
